# Metabolomics: a search for biomarkers of visceral fat and liver fat content

**DOI:** 10.1007/s11306-019-1599-x

**Published:** 2019-10-05

**Authors:** Sebastiaan Boone, Dennis Mook-Kanamori, Frits Rosendaal, Martin den Heijer, Hildo Lamb, Albert de Roos, Saskia le Cessie, Ko Willems van Dijk, Renée de Mutsert

**Affiliations:** 1 Department of Clinical Epidemiology, Leiden University Medical Center, Postal Zone C7-P, PO Box 9600, 2300 RC Leiden, The Netherlands; 2 Department of Public Health and Primary Care, Leiden University Medical Center, Leiden, The Netherlands; 3 Department of Radiology, Leiden University Medical Center, Leiden, The Netherlands; 4 Department of Biomedical Data Sciences, Section Medical Statistics and Bioinformatics, Leiden University Medical Center, Leiden, The Netherlands; 5 Department of Endocrinology, Leiden University Medical Center, Leiden, The Netherlands; 6 Einthoven Laboratory for Experimental Vascular Medicine, Leiden University Medical Center, Leiden, The Netherlands; 7 Department of Human Genetics, Leiden University Medical Center, Leiden, The Netherlands; 8 Department of Endocrinology, VU Medical Centre, Amsterdam, The Netherlands

**Keywords:** Visceral adipose tissue, Liver fat, Metabolomics, Biomarkers

## Abstract

**Intoduction:**

Excess visceral and liver fat are known risk factors for cardiometabolic disorders. Metabolomics might allow for easier quantification of these ectopic fat depots, instead of using invasive and costly tools such as MRI or approximations such as waist circumference.

**Objective:**

We explored the potential use of plasma metabolites as biomarkers of visceral adipose tissue (VAT) and hepatic triglyceride content (HTGC).

**Methods:**

We performed a cross-sectional analysis of a subset of the Netherlands Epidemiology of Obesity study. Plasma metabolite profiles were determined using the Biocrates Absolute*IDQ* p150 kit in 176 individuals with normal fasting plasma glucose. VAT was assessed with magnetic resonance imaging and HTGC with proton-MR spectroscopy. We used linear regression to investigate the associations of 190 metabolite variables with VAT and HTGC.

**Results:**

After adjustment for age, sex, total body fat, currently used approximations of visceral and liver fat, and multiple testing, three metabolite ratios were associated with VAT. The strongest association was the lysophosphatidylcholines to total phosphatidylcholines (PCs) ratio [− 14.1 (95% CI − 21.7; − 6.6) cm^2^ VAT per SD of metabolite concentration]. Four individual metabolites were associated with HTGC, especially the diacyl PCs of which C32:1 was the strongest at a 1.31 (95% CI 1.14; 1.51) fold increased HTGC per SD of metabolite concentration.

**Conclusion:**

Metabolomics may be a useful tool to identify biomarkers of visceral fat and liver fat content that have added diagnostic value over current approximations. Replication studies are required to validate the diagnostic value of these metabolites.

**Electronic supplementary material:**

The online version of this article (10.1007/s11306-019-1599-x) contains supplementary material, which is available to authorized users.

## Introduction

Abdominal obesity, in particular excess visceral adipose tissue and intra-hepatic fat, are well-established risk factors for cardiovascular disease and type 2 diabetes (Tchernof and Despres 2013). Measurements of visceral fat or liver fat could therefore improve the prediction of cardiometabolic disease. Both visceral and liver fat are located within the abdominal cavity and can be directly assessed using expensive imaging techniques or invasive biopsies which are not feasible to perform in routine care settings. As proxies, easy and inexpensive measurements such as waist circumference (Pouliot et al. 1994) or the fatty liver index (Bedogni et al. 2006) are often used. Unfortunately, these methods are vulnerable to misclassification and appear to mainly discriminate between individuals with high or low risk of excess visceral or liver fat (Cuthbertson et al. 2014; Tchernof and Despres 2013; Zelber-Sagi et al. 2013).

In the past decade, there has been an increasing interest in the application of metabolomics techniques in the study of cardiometabolic conditions (Abu Bakar et al. 2015). Because metabolites are affected by both endogenous regulatory mechanisms as well as by interactions with the environment, such as diet, they are measures that potentially lie close to an individuals’ phenotype which makes them interesting targets for biomarker research. Several metabolomics studies have previously been performed to investigate the relation between metabolites and body fat distribution (Bachlechner et al. 2016; Boulet et al. 2015; Feldman et al. 2017; Kalhan et al. 2011; Martin et al. 2013; Rauschert et al. 2016; Siegert et al. 2013; Szymanska et al. 2012). However, these studies were performed in selected populations such as bariatric surgery patients (Boulet et al. 2015) or trial participants (Szymanska et al. 2012), did not relate metabolites to quantified measures of visceral or liver fat but rather to the presence/absence of non-alcoholic fatty liver disease (Feldman et al. 2017; Kalhan et al. 2011) or approximate measures such as body mass index or waist circumference (Bachlechner et al. 2016; Rauschert et al. 2016). With some exceptions (Siegert et al. 2013; Szymanska et al. 2012) none of these studies examined the diagnostic potential of metabolites over current approximations such as waist circumference.

Given the difficulty of measuring intra-abdominal fat and limitations in previous studies, we aim here to explore the potential of metabolites as candidate biomarkers of visceral fat and liver fat in a sample of individuals from a population-based cohort. We examined the associations between metabolite concentrations, measured with a commercially available, targeted mass spectrometry-based metabolomics panel, and directly assessed visceral fat by magnetic resonance imaging (MRI) and hepatic triglyceride content measured by proton magnetic resonance spectroscopy (^1^H-MRS). In addition, we aimed to examine to what extent these metabolites provide additional information over conventional approximation methods of visceral fat volume and liver fat content.

## Methods

### Study design and study population

The Netherlands Epidemiology of Obesity (NEO) study is a prospective, population-based cohort study aimed at investigating the pathways leading to obesity-related conditions (de Mutsert et al. 2013). During the period between 2008 and 2012 a total of 6671 participants aged 45–65 years, with an oversampling of persons with overweight, from the Leiden greater area were included. Persons aged between 45 and 65 years with a self-reported body mass index (BMI) of ≥ 27 kg/m^2^ were invited to participate through letters from their general practitioner and municipalities, as well as through local advertisements. Additionally, all inhabitants aged 45–65 years from one municipality, Leiderdorp, were invited irrespective of their BMI, allowing a reference distribution of BMI.

Participants were invited for a baseline visit at the NEO study center of the Leiden University Medical Center (LUMC) after an overnight fast. Prior to this study visit, participants completed a general questionnaire at home to report demographic, lifestyle and clinical information. Participants were asked to bring all medication they were using in the month preceding the baseline study visit and research nurses recorded names and dosages of all medication. Participants came to the research site in the morning and completed a screening form, asking about anything that might create a health risk or interfere with MRI (most notably metallic devices, claustrophobia or a body circumference of more than 1.70 m). Approximately 35% of the participants without potential MRI contra-indications were randomly selected to undergo direct assessment of the amount of visceral adipose tissue (VAT) by MRI in combination with hepatic triglyceride content (HTGC) by ^1^H-MRS. All participants underwent an extensive physical examination, including anthropometry and blood sampling.

The present study is a cross-sectional analysis of baseline measurements of a subgroup of 176 participants with normal fasting plasma glucose concentrations (≤ 6.0 mmol/L) and without any lipid or glucose lowering medication who were randomly sampled from the NEO study for a previous nested case–control metabolomics study (Mook-Kanamori et al. 2016). The previous study also included individuals with elevated fasting glucose concentrations, which we excluded because of concerns over potential selection bias by including these individuals. Additionally, because elevated fasting glucose concentrations are reasonably rare in the general population, we assumed the estimates from individuals with a normal fasting glucose concentration to be similar to those of a random sample from the NEO study population. We excluded one participant without information on the amount of VAT or HTGC. Another 25 individuals had missing information on HTGC alone due to a failed liver scan and were excluded from the analyses involving HTGC. As an additional quality control step, we excluded one participant with a mismatch (> 1.5 standard deviation (SD)) between standard glucose measurements and hexose sugar concentrations (which consist of > 90% glucose) determined through the metabolomics assay. Our final sample therefore consisted of 174 participants for the analyses on VAT and 149 for the analyses on HTGC.

The study was approved by the medical ethics committee of the Leiden University Medical Centre (LUMC) and all participants gave written informed consent.

### Data collection

#### General

Participants reported information on ethnicity, education, smoking, menopause and hormone use on the questionnaires. Participants could choose from eight categories of ethnicity, which we subsequently grouped into ‘white’ and ‘other’. The highest level of education was reported from ten categories according to the Dutch educational system. We grouped these education levels into high (higher vocational school, university and post-graduate education) versus low education. Smoking was categorized as current, former or never smoker.

At the study center, body weight and total body fat were estimated using a Tanita bio impedance balance (TBF-310, Tanita, International division, UK) without shoes and 1 kg was subtracted from the body weight. BMI (kg/m^2^) was calculated by dividing the weight in kilograms by the height in meters squared. Waist circumference (cm) was measured mid-way between the border of the lower costal margin and the iliac crest.

#### Blood sampling and metabolomics measurements

A fasting blood sample was obtained from the antecubital vein and standard laboratory analyses such as glucose and lipid profile determination were performed at the clinical chemical laboratory of the LUMC, as described previously (de Mutsert et al. 2013). Metabolomics measurements were performed in fasting blood samples at the Genome Analysis Center at the Helmholtz Zentrum München, Germany using the Biocrates Absolute IDQ™ p150 kit (BIOCRATES Life Science AG, Innsbruck, Austria) and ESI-FIA-MS/MS measurements. The p150 assay covers a wide range of acylcarnitines (LIPID MAPS subclass: fatty acyl carnitines), sphingolipids (LIPID MAPS subclass: ceramide phosphocholines), lysophosphatidylcholines, diacyl and acyl-alkyl phosphatidlycholines (LIPID MAPS subclass: glycerophosphocholines), complemented by a set of amino acid measures and hexose sugar concentrations. In total, the assay measures 163 metabolites and additionally calculates 27 aggregate measures consisting of sums and ratios of the different metabolite classes for a total of 190 metabolite related variables (Online Resource 1). The assay was applied following the manufacturer’s instructions and has been described in detail before (Römisch-Margl et al. 2012). For the present study, all individual metabolites as well as the aggregate measures were used in the analyses.

#### Assessment of visceral adipose tissue and hepatic triglyceride content

Imaging was performed on a 1.5 Tesla MR system (Philips Medical Systems, Best, the Netherlands). Abdominal visceral fat depots were quantified by MRI using a turbo spin echo imaging protocol. At the level of the fifth lumbar vertebra three transverse images each with a slice thickness of 10 mm were obtained during a breath-hold. Abdominal visceral fat areas were converted from the number of pixels to centimeters squared, using in-house-developed software (MASS, Medis, Leiden, The Netherlands) and the average of the three slices was used in our analyses (Hammer et al. 2008).

Hepatic triglyceride content was quantified by ^1^H-MRS of the liver as described previously (Hammer et al. 2008). Briefly, an 8-mL voxel was positioned in the right lobe of the liver. Spectra were obtained with and without water suppression with free breathing and fitted using Java based MR user interface software (jMRUI version 3.0, Leuven, Belgium) (Naressi et al. 2001). Mean line widths were calculated. The resonances of methylene and methyl were fitted and used for calculation of triglycerides. HTGC relative to water was calculated as (signal amplitude of triglyceride)/(signal amplitude of water) × 100.

### Statistical analysis

In the NEO study, persons with a BMI of 27 kg/m^2^ or higher have been oversampled. To correctly represent associations for the general population (Korn and Graubard 1991), adjustments for the oversampling of participants with a BMI ≥ 27 kg/m^2^ were made. This was done by weighting individuals towards the BMI distribution of participants from the Leiderdorp municipality (Lumley 2004), whose BMI distribution was similar to the BMI distribution of the general Dutch population (VWS 2013). All results are based on weighted analyses. Consequently, the results apply to a population-based study without oversampling of participants with a BMI ≥ 27 kg/m^2^.

Baseline characteristics are expressed as mean (SD), median (25th–75th percentile) or proportion (%) and stratified by sex. We calculated z-scores and standardized all metabolites to a mean of zero and a standard deviation of one. Hepatic triglyceride content was ln-transformed because its distribution was strongly skewed.

For model 1, we performed crude linear regressions to calculate the regression coefficients of all 190 metabolite variables with VAT and HTGC. Subsequently, we adjusted for age, sex and total body fat in model 2 to investigate if the associations were specific for VAT or HTGC, regardless of total body fat. In model 3, we additionally adjusted for waist circumference, fasting triglycerides, high density lipoprotein cholesterol and total cholesterol concentrations. The measurements added in this final model are easily measured and frequently used in approximation methods for visceral (Amato et al. 2010; Lemieux et al. 2000) and liver fat (Bedogni et al. 2006). We adjusted for these approximations to determine if metabolites remained associated on top of them and thereby potentially provide additional information on VAT or liver fat. We compared the adjusted explained variance (adjusted R^2^) of the model with the covariates from model 3 without metabolites to the model where the metabolites that remained significantly associated with VAT or HTGC after adjusting for these covariates were added to assess if metabolites resulted in an increase of the adjusted R^2^. We added each metabolite individually, as well as all possible combinations (e.g. two, three or more metabolites) if multiple metabolites remained significantly associated with VAT or HTGC. We considered our sample size too small to make reliable statements about associations in men and women separately, however previous studies have delineated sex differences in intra-abdominal fat distribution (Tchernof and Despres 2013) and in associations of metabolites with intra-abdominal fat (Bachlechner et al. 2016; Szymanska et al. 2012). Therefore, we stratified our analyses by sex but did not perform significance tests.

The regression coefficients for visceral fat can be interpreted as the mean difference in VAT (cm^2^) per standard deviation (SD) of metabolite concentration. Because hepatic triglyceride content was ln-transformed, we back transformed the regression coefficients which therefore represent a ratio. This ratio can be interpreted as a relative increase in HGTC per SD of metabolite concentration. For example, a ratio of 1.5 indicates that an increase of one SD of metabolite concentration is associated with a 1.5-fold increased HTGC.

We corrected for multiple testing using the false discovery rate (FDR) method at 5% for each set of regressions that use the 190 metabolite variables, assuming they represent independent tests. All statistical analyses were performed in STATA 14.1 and heatmaps were generated in R 3.5.2 using the heatmap.2 function from the gplots package.

## Results

### Participant characteristics

Characteristics for the 174 individuals with VAT measurements are summarized in Table 1. Men and women were of similar age and ethnicity, more men had a higher education, men consumed more alcohol, and had a higher BMI, waist circumference, HTGC and VAT, while women had more total body fat than men.

**Table 1:** Characteristics of 174 participants of the NEO study with fasting glucose ≤ 6.0 mmol/L and with metabolomics and visceral adipose tissue measurements

	Men (n = 84)	Women (n = 90)
Demographic/anthropometric		
Age (years)	57.0 (46.0–65.0)	56.0 (47.0–65.0)
Ethnicity (% white)	93	95
Education level (% high)^a^	63	41
Smoking (%)		
Never	46	49
Former	42	41
Current	12	10
Alcohol consumption (g/day)	13.0 (0.4–52.5)	4.3 (0–21.3)
Peri- or postmenopausal (%)	–	78
Hormone use (% current)^b^	–	19
BMI (kg/m^2^)	25.5 (3.1)	24.0 (4.2)
Total body fat (%)	22.7 (5.0)	33.3 (7.1)
Waist circumference (cm)	93.0 (77.0–111.0)	80.0 (68.0–101.0)
Visceral adipose tissue, mean (cm^2^)	109.5 (60.9)	58.6 (38.4)
Hepatic triglyceride content (%)	3.4 (1.0–18.7)	1.4 (0.4–7.7)
Fasting blood concentrations		
Glucose (mmol/L)	5.3 (0.4)	5.0 (0.5)
Total cholesterol (mmol/L)	5.5 (0.9)	5.8 (1.2)
Triglycerides (mmol/L)	0.9 (0.4–2.3)	0.7 (0.3–1.8)
HDL-cholesterol (mmol/L)	1.4 (0.3)	1.8 (0.4)
LDL-cholesterol (mmol/L)	3.6 (0.7)	3.5 (1.1)

Results are based on analyses weighted towards the BMI distribution of the general population. Values represent means (SD), medians (90% range) or percentages. Measurements were available for all participants except for the following variables: education (men N = 84, women N = 89), hepatic triglyceride content (men N = 70, women N = 79)

*BMI* body mass index, *HDL* high-density lipoprotein, *LDL* low-density lipoprotein

^a^High education: higher vocational school, university and post-graduate education

^b^Use of contraceptives or hormone replacement therapy at the time of study visit

### Metabolites and visceral fat

In the total population using the crude model, shorter chained lysophosphatidylcholines (lysoPC), acyl-alkyl phosphatidylcholines (acyl-alkyl PCs) and sphingomyelins (SM) overall related negatively with VAT, while diacyl phosphatidylcholines (PC) were mostly positively related to VAT (Online Resource 2a). Thirty-nine individual metabolites and six of the aggregate measures were significantly associated with VAT after FDR correction (Online Resource 4). Adjusting for the factors age, sex and total body fat in model 2 (Online Resource 2b) and 3 (Online Resource 2c) diminished the strength of most relations. Only the ratio of lysophosphatidylcholines to total phosphatidylcholines (lysoPC/PC), sphingomyelins to total sphingomyelins and phosphatidylcholines (SM/(SM + PC)) and sphingomyelins to total phosphatidylcholines (SM/PC) were significantly associated (Online Resource 2b and Table 2). In model 3, the same measures remained associated at similar strengths (Online Resource 2c and Table 3). These three measures were strongly correlated at values from 0.32 to 1.00 (Online Resource 5). Association estimates were not always consistent amongst men and women and in all models were often larger in men (Online Resource 2). In model 3 for example, the largest inverse estimate in men was that of SM C16:0 at − 25.4 (− 41.5, − 9.2) cm^2^ per SD of metabolite concentration while in women the estimate was − 1.9 (− 10.3, 6.5) cm^2^ per SD of metabolite concentration.

**Table 2:** Metabolites associated with visceral adipose tissue (N = 174) or hepatic triglyceride content (N = 149) after false discovery rate correction in model 2, adjusted for age, sex and total body fat

	Visceral adipose tissue					
	Total (N = 174) Estimate (95% CI)	P value	Men (N = 84) Estimate (95% CI)	P value	Women (N = 90) Estimate (95% CI)	P value
Aggregate measures						
Total lysoPC/total PC	− 15.5 (− 23.0; − 8.0)*	6.85E−05	− 25.4 (− 41.4; − 9.5)	2.16E−03	− 6.2 (− 11.0; − 1.4)	1.18E−02
Total SM/(total SM + total PC)	− 12.1 (− 17.8; − 6.3)	5.01E−05	− 13.9 (− 24.1; − 3.7)	8.02E−03	− 7.2 (− 12.2; − 2.2)	4.96E−03
Total SM/total PC	− 12.1 (− 17.8; − 6.4)	4.89E−05	− 14.0 (− 24.2; − 3.8)	7.59E−03	− 7.2 (− 12.1; − 2.2)	5.03E−03

	Hepatic triglyceride content					
	Total (N = 149) Estimate (95% CI)	*P* value	Men (N = 70) Estimate (95% CI)	P value	Women (N = 79) Estimate (95% CI)	P value
Lysophosphatidylcholines						
Lyso PC a C14:0	1.30 (1.17; 1.45)	3.64E−06	1.33 (1.15; 1.54)	2.02E−04	1.20 (1.04; 1.39)	1.57E−02
Diacyl phosphatidylcholines						
PC aa C28:1	1.23 (1.07; 1.41)	4.50E−03	1.10 (0.87; 1.38)	4.20E−01	1.18 (0.98; 1.44)	8.59E−02
PC aa C30:0	1.30 (1.14; 1.47)	9.32E−05	1.34 (1.11; 1.62)	3.37E−03	1.19 (1.01; 1.40)	4.16E−02
PC aa C32:1	1.38 (1.23; 1.55)	9.12E−08	1.37 (1.12; 1.68)	2.82E−03	1.31 (1.13; 1.52)	4.03E−04
PC aa C32:2	1.32 (1.17; 1.49)	2.05E−05	1.41 (1.18; 1.68)	2.48E−04	1.23 (1.05; 1.44)	1.16E−02
PC aa C34:1	1.22 (1.08; 1.38)	1.26E−03	1.14 (0.92; 1.41)	2.24E−01	1.18 (1.00; 1.38)	4.54E−02
PC aa C34:3	1.33 (1.16; 1.52)	6.96E−05	1.33 (1.05; 1.68)	1.86E−02	1.25 (1.06; 1.47)	7.80E−03
PC aa C34:4	1.36 (1.20; 1.53)	1.84E−06	1.36 (1.09; 1.70)	7.56E−03	1.28 (1.10; 1.49)	1.42E−03
PC aa C36:1	1.35 (1.20; 1.52)	1.04E−06	1.29 (0.93; 1.78)	1.26E−01	1.30 (1.15; 1.46)	4.09E−05
PC aa C36:2	1.26 (1.11; 1.43)	5.55E−04	1.14 (0.88; 1.47)	3.27E−01	1.23 (1.05; 1.43)	9.10E−03
PC aa C36:3	1.21 (1.06; 1.37)	3.77E−03	1.11 (0.86; 1.42)	4.22E−01	1.16 (0.97; 1.38)	9.69E−02
PC aa C36:6	1.24 (1.06; 1.44)	6.68E−03	1.18 (0.92; 1.52)	1.95E−01	1.18 (0.96; 1.46)	1.13E−01
PC aa C38:3	1.41 (1.26; 1.59)	2.05E−08	1.43 (1.12; 1.82)	4.48E−03	1.32 (1.14; 1.54)	4.61E−04
PC aa C38:5	1.20 (1.06; 1.36)	5.36E−03	1.05 (0.84; 1.32)	6.46E−01	1.21 (1.04; 1.42)	1.74E−02
PC aa C40:4	1.23 (1.10; 1.38)	3.25E−04	1.13 (0.91; 1.40)	2.62E−01	1.19 (1.04; 1.38)	1.40E−02
PC aa C40:5	1.36 (1.21; 1.52)	6.78E−07	1.30 (1.04; 1.63)	2.40E−02	1.32 (1.14; 1.53)	3.65E−04
Sphingomyelins						
SM C22:3	0.84 (0.75; 0.94)	2.36E−03	0.93 (0.74; 1.18)	5.61E−01	0.85 (0.76; 0.97)	1.20E−02
Amino acids						
Tryptophan	1.20 (1.07; 1.35)	2.29E−03	1.28 (1.05; 1.55)	1.59E−02	1.05 (0.90; 1.23)	5.06E−01
Tyrosine	1.33 (1.10; 1.60)*	3.87E−03	1.53 (1.26; 1.86)	3.97E−05	1.16 (0.91; 1.48)	2.28E−01
Aggregate measures						
Aromatic amino acids (AAA)	1.29 (1.11; 1.50)	1.21E−03	1.41 (1.16; 1.72)	7.52E−04	1.13 (0.91; 1.40)	2.58E−01
MUFA(PC)	1.27 (1.13; 1.44)	1.06E−04	1.20 (0.94; 1.53)	1.44E−01	1.22 (1.05; 1.42)	9.05E−03
PUFA(PC)	1.24 (1.08; 1.42)	2.01E−03	1.11 (0.86; 1.43)	4.36E−01	1.19 (1.01; 1.42)	4.02E−02
Total PC + total SM	1.23 (1.07; 1.40)	2.84E−03	1.08 (0.84; 1.39)	5.44E−01	1.19 (1.02; 1.40)	3.25E−02
Total PC	1.26 (1.10; 1.43)	7.36E−04	1.14 (0.88; 1.48)	3.32E−01	1.21 (1.03; 1.43)	2.24E−02
Total diacyl PC	1.27 (1.12; 1.45)	3.21E−04	1.17 (0.90; 1.52)	2.34E−01	1.22 (1.04; 1.43)	1.82E−02
Total SM/(total SM + total PC)	0.80 (0.71; 0.89)	1.42E−04	0.76 (0.63; 0.92)	5.12E−03	0.87 (0.74; 1.02)	8.81E−02
Total SM/total PC	0.80 (0.71; 0.89)	1.66E−04	0.76 (0.63; 0.92)	5.68E−03	0.87 (0.74; 1.03)	9.48E−02
Tyrosine/phenylalanine	1.30 (1.11; 1.52)*	1.35E−03	1.54 (1.24; 1.92)	1.93E−04	1.15 (0.96; 1.39)	1.27E−01

Metabolites that reached the FDR adjusted P value in the total group are summarized. The reported numbers represent regression outcomes (95% CI) from model 2, correcting for age, sex and total body fat, expressed as the difference in VAT (cm^2^) per SD of metabolite concentration and the relative increase in HTGC per SD of metabolite concentration. *Indicates associations with a statistically significant interaction with sex

**Table 3:** Metabolites associated with visceral fat (N = 174) or hepatic triglyceride content (N = 149) after false discovery rate correction in model 3, adjusted for age, sex, total body fat, waist circumference and fasting serum concentrations of triglycerides, HDL cholesterol and total cholesterol

	Visceral adipose tissue					
	Total (N = 174) Estimate (95% CI)	P value	Men (N = 84) Estimate (95% CI)	P value	Women (N = 90) Estimate (95% CI)	P value
Aggregate measures						
Total lysoPC/total PC	− 14.1 (− 21.7; − 6.6)*	3.05E−04	− 20.4 (− 37.2; − 3.5)	1.82E−02	− 8.0 (− 13.1; − 2.9)	2.37E−03
Total SM/(total SM + total PC)	− 13.5 (− 20.3; − 6.8)*	1.05E−04	− 19.0 (− 31.4; − 6.6)	3.03E−03	− 9.4 (− 15.3; − 3.5)	2.10E−03
Total SM/total PC	− 13.5 (− 20.1; − 6.8)*	1.02E−04	− 19.0 (− 31.4; − 6.7)	2.87E−03	− 9.3 (− 15.2; − 3.5)	2.16E−03

	Hepatic triglyceride content					
	Total (N = 149) Estimate (95% CI)	P value	Men (N = 70) Estimate (95% CI)	P value	Women (N = 79) Estimate (95% CI)	P value
Lysophosphatidylcholines						
Lyso PC a C14:0	1.19 (1.08; 1.32)	7.79E−04	1.23 (1.09; 1.39)	8.94E−04	1.17 (1.01; 1.36)	3.78E−02
Diacyl phosphatidylcholines						
PC aa C32:1	1.31 (1.14; 1.51)	2.77E−04	1.37 (1.11; 1.70)	3.98E−03	1.26 (1.04; 1.52)	1.88E−02
PC aa C36:1	1.30 (1.13; 1.50)	3.77E−04	1.19 (0.90; 1.58)	2.15E−01	1.31 (1.11; 1.55)	1.59E−03
PC aa C40:5	1.27 (1.11; 1.44)	4.64E−04	1.14 (0.95; 1.35)	1.57E−01	1.28 (1.07; 1.53)	7.50E−03

Metabolites that reached the FDR adjusted P value in the total group are summarized. The reported numbers represent regression outcomes (95% CI) from model 3, correcting for age, sex, total body fat percentage, waist circumference and fasting concentrations of triglycerides, HDL and total cholesterol, expressed as the difference in VAT (cm^2^) per SD of metabolite concentration and the relative increase in HTGC per SD of metabolite concentration. *Indicates associations with a statistically significant interaction with sex

**Fig. 1:**
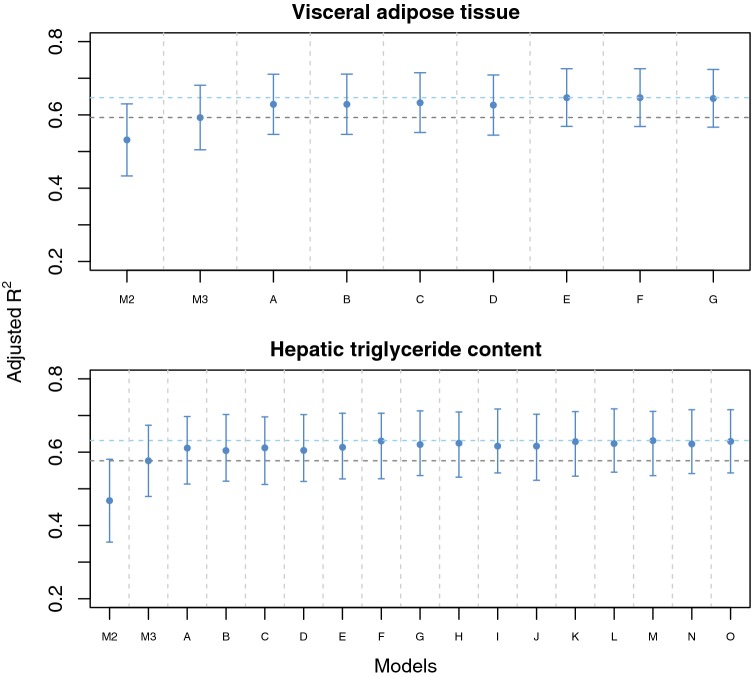
Adjusted explained variance (R^2^) before and after adding metabolites to conventional approximation measures of VAT and HTGC. Model 2 (M2, age, sex and total body fat) and model 3 (M3, model 2 + waist circumference and fasting concentrations of glucose, triglycerides, HDL-cholesterol and total cholesterol) are represented by the two leftmost columns. The other models consist of model 3 plus all possible combinations of metabolites significantly associated with either VAT (N = 3) or HTGC (N = 4) in the total sample under model 3. The lower dashed horizontal line indicates the variance explained by model 3, while the upper dashed horizontal line indicates the maximum adjusted R^2^ attained by adding metabolites Visceral fat models A–G contain the following metabolites: (A) total SM/total SM + PC, (B) total SM/total PC, (C) total lysoPC/total PC, (D) total SM/total PC and total SM/total SM + PC, (E) total lysoPC/total PC and total SM/total SM + PC, (F) total lysoPC/total PC and total SM/total PC, (G) total lysoPC/total PC, total SM/total SM + PC and total SM/PC. Hepatic triglyceride models A–O contain the following metabolites: (A) lysoPC a C14:0, (B) PC aa C32:1, (C) PC aa C36:1, (D) PC aa C40:5, (E) lysoPC a C14:0 and PC aa C32:1, (F) lysoPC a C14:0 and PC aa C36:1, (G) lysoPC a C14:0 and PC aa C40:5, (H) PC aa C32:1 and C36:1, (I) PC aa C32:1 and C40:5, (J) PC aa C36:1 and C40:5, (K) lysoPC a C14:0 and PC aa C32:1 and C36:1, (L) lysoPC a C14:0 and PC aa C32:1 and C40:5, (M) lysoPC a C14:0 and PC aa C36:1 and C40:5. (N) PC aa C32:1, C36:1 and C40:5, (O) lysoPC a C14:0 and PC aa C32:1, C36:1 and 40:5

The variance explained (adjusted R^2^) by the factors from model 3 (age, sex, total body fat, waist circumference, and fasting concentrations of triglycerides, HDL cholesterol and total cholesterol), in the total population was 0.59. Adding all three metabolites that were significantly associated after adjusting for the factors from model 3 resulted in an adjusted R^2^ of 0.65 in the total population, although a similar result was achieved after adjusting for two of the metabolites (the ratio of lysoPC/total PC and SMs to total SMs and PCs, Fig. 1).

### Metabolites and hepatic triglyceride content

In the total sample, most metabolites related positively with HTGC, except the acyl-alkyl PCs which were negatively related to HTGC in the crude model. After FDR correction, 33 individual and 8 aggregate metabolite variables were significantly associated (Online Resource 3a and Online Resource 4). The strength of all relations was diminished after the adjustments in model 2 (Online Resource 3b) and further in model 3 (Online Resource 3c). There remained 19 individual and 9 aggregate variables with significant associations in model 2 (Online Resource 3b and Table 2). In particular, 15 of these 19 individual metabolites were diacyl PCs which were all positively associated. The other significant associations included tryptophan, tyrosine, lysoPC C14:0, SM C22:3, the sums of aromatic amino acids, mono-unsaturated fatty acids (in PCs), poly-unsaturated fatty acids (in PCs), PCs and SMs, phosphatidylcholines (PCs), diacyl PCs, and the SM/(SM + PC), SM/PC, tyrosine/phenylalanine ratios. In model 3, only diacyl PCs C32:1, 36:1 and 40:5 and lysoPC14:0 remained associated (Online Resource 3c and Table 3). These metabolites were quite strongly correlated at values from 0.36 to 0.71 (Online Resource 5). Similar to visceral fat, association estimates were not always consistent between men and women (Online Resource 3). In some cases, the direction of the associations even appears to be reversed such as for histidine which in model 3 had an estimate of 1.28 (1.06, 1.53) change in HTGC per SD of metabolite concentration in men and 0.91 (0.80, 1.04) change in HTGC per SD of metabolite concentration in women.

The adjusted R^2^ for all variables from model 3 was 0.58 in the total sample. After adding all four significant metabolites the R^2^ increased to 0.63, although a similar result could be achieved only two metabolite variables (diacyl PC C32:1 and 40:5, Fig. 1).

## Discussion

In the present study we used a commercially available, targeted metabolomics assay to estimate the association between metabolites and directly assessed VAT and HTGC. We observed associations that were specific for VAT or HTGC rather than overall obesity as several metabolites variables remained associated, even after adjusting for total body fat. The metabolites that remained associated in the fully adjusted model were also specific for either VAT or HTGC, as the lysoPC/total PC, SM/(total SM + PC) and total SM/total PC ratios remained associated with VAT, whereas lysoPC C14:0 and the diacyl PCs C32:1, 36:1 and 40:5 remained associated with HTGC. Adding these metabolites to a model with currently used approximation measures modestly improved the explained variance of the model for both VAT and HTGC. Despite our small sample size we observed some evidence for differences in the metabolite associations between men and women, as the size of the estimates were frequently different between men and women and possibly even reversed for some metabolites. Alternative methods of selecting metabolites as potential biomarkers, such as LASSO regression can be performed. However, when we explored the use of LASSO regression in our limited sample size, a large number of metabolites was selected, and this selection was inconsistent over several LASSO repeats. Therefore, we advise such an approach to be applied in larger sample sizes.

Our findings are in line with some previous studies on body fat distribution and metabolite profiles. Several authors have reported associations of phosphocholine lipids, including phosphatidylcholines and lysophosphatidylcholine, as well as sphingomyelins with visceral fat (Boulet et al. 2015; Martin et al. 2013; Scherer et al. 2015; Syme et al. 2016; Szymanska et al. 2012) or proxies of visceral fat such as waist circumference (Bachlechner et al. 2016; Rauschert et al. 2016). In contrast to many of these studies, we did not identify individual metabolites that were specifically associated with visceral fat, but we did identify associations of aggregate measures of these metabolites. This discrepancy might be partially explained by the fact that we measured a different subset of lipids than some other studies. However, we also did not detect visceral fat specific amino acid associations even though these are measured in most metabolomics studies and have been frequently associated with visceral fat before (Boulet et al. 2015; Martin et al. 2013). The reason for this difference is unclear but could be related to the fact that various studies did not take overall obesity or fat mass into account. Because overall fat mass and visceral fat are highly correlated (Martin et al. 2003), some of the previously reported associations might reflect associations with overall fat mass rather than with visceral fat specifically. This hypothesis is partially supported by our findings, as several amino acids associated with VAT in the unadjusted model (Online Resource 2) but not in the model adjusting for sex, age and total body fat. The diagnostic use of metabolites for visceral fat accumulation has not been extensively explored yet, as most studies on visceral fat metabolomics have been focused on increasing insight into biological pathways related to visceral fat. One study tested the inclusion of metabolites on top of a large number of other phenotypes including, age, sex, total body fat and waist circumference and observed no improvement in the explained variance of visceral fat in women, while in men a variable set including phosphatidylcholine C32:0 and acetate improved the R^2^ from 0.485 to 0.784 (Szymanska et al. 2012). However, given that their sample size in which the R^2^ was calculated was only thirty-nine for women and fifteen for men, it is possible that their models were somewhat overfitted. Although our sample size was larger, overfitting to some extent could also explain our results as we lacked external validation and fitted models of eight to eleven variables in our sample of 174 individuals.

With regard to liver fat, previous studies have identified similar associations as we observed between acylcarnitines, phosphocholine containing lipids, sphingomyelins and aromatic amino acids and increased liver fat content (Feldman et al. 2017; Kaikkonen et al. 2017; Kalhan et al. 2011; Orešič et al. 2013; Siegert et al. 2013; Zhou et al. 2016). Interestingly, both mono- and polyunsaturated phosphatidylcholines associated positively with hepatic triglyceride content in our study, while increased liver fat content is generally associated with lower concentrations of PUFAs and increased concentrations of saturated or mono-unsaturated lipids (Kaikkonen et al. 2017; Puri et al. 2009). The reason for this difference however is unclear. Several of these studies have also explored the diagnostic use of metabolites for hepatic steatosis and found that metabolite-based models could reasonably discriminate between individuals with and without hepatic steatosis (Feldman et al. 2017; Orešič et al. 2013; Siegert et al. 2013; Zhou et al. 2016). However, with the exception of one study that also measured liver fat using ^1^H-MRS (Orešič et al. 2013) these studies only had data on hepatic fat content in the form of ultrasound or biopsy proven hepatic steatosis and so could not derive diagnostic models that made a quantitative estimation of liver fat content. We identified one previous study that also checked the performance of metabolite-based methods against current approximation methods (Siegert et al. 2013). The authors observed an improved diagnostic performance of models including metabolites such as lysoPCs, diacyl and acyl-alkyl PCs, acylcarnitines and amino acids including tyrosine, compared with models using only conventional measures from the fatty liver index (Bedogni et al. 2006).

Although the metabolites that remained associated within our sample were not identical for visceral and liver fat, it cannot be concluded that there is no overlap in the associations between visceral and liver fat based on our data alone. First, the metabolites that remained associated in the final sample, although not identical, are related to similar classes in both fat depots. For example, lysoPC a C14:0 which associated with liver fat and total lysoPC/total PC which associated with visceral fat are both related to the lysophoshatidylcholine class. Similarly the diacyl PCs C32:6, 36:1 and 40:5 which were associated with liver fat are components of the total SM/total SM + PC and total SM/total PC variables associated with visceral fat. This supports the presence of common mechanisms underlying both visceral fat and liver fat (Tchernof and Despres 2013).

The main value of our study lies in contributing further evidence for the use of metabolomics in diagnostic approaches of visceral and liver fat and to encourage further exploration of this topic. Currently, only imaging techniques such as computed tomography, magnetic resonance-based techniques and to a lesser extent ultrasonography can quantify lipid accumulation in visceral adipose tissue or the liver (Fang et al. 2018; Karlas et al. 2014; Koot et al. 2014; Schwimmer et al. 2015). Several approaches, such as the hypertriglyceridemic waist phenotype (Lemieux et al. 2000), exist that combine anthropometric measurements such as waist circumference and serum concentrations of parameters such as high-density lipoproteins to identify individuals who are likely to have excess liver (Bedogni et al. 2006) or visceral fat (Amato et al. 2010; Kahn 2005; Lemieux et al. 2000). However, these methods tend to be designed to make qualitative rather than quantitative predictions of whether individuals have excess lipid accumulation (Cuthbertson et al. 2014; Neamat-Allah et al. 2015; Vongsuvanh et al. 2012; Zelber-Sagi et al. 2013). Nevertheless, quantitative predictions could be useful for cardiovascular disease risk prediction or to monitor the progress of interventions aimed at reducing visceral or liver fat. Imaging modalities are not practical for this purpose because they are generally expensive and require specialized personnel and are therefore not suited for large scale use in general clinical practice. Because of the limitations of these currently available methods, we propose that metabolite-based methods warrant further investigation. Many health care laboratories are already equipped to perform metabolites measurements, so a panel of metabolites to diagnose lipid accumulation would be a comparatively quick and practical method that could be performed in tandem with blood testing that is already part of cardiovascular risk assessment.

The pathophysiological role of the metabolite associations we observed is not completely understood. Phosphatidylcholines are an important component of cell membranes and lipoproteins (Cole et al. 2012) and in addition to their structural role appear to be involved in for example the secretion of very large density lipoproteins by the liver as well as glucose regulation (Cole et al. 2012; Furse and Kroon 2015). Indeed, changes in the concentrations of phosphocholine containing lipids have been associated with cardiometabolic alterations associated with excess liver and visceral fat such as insulin resistance (Floegel et al. 2013) and atherosclerosis (Matsumoto et al. 2007). Similarly, sphingomyelins are an important component of cell membranes and also seem to be involved in the development of insulin resistance (Li et al. 2011). Tyrosine concentrations also associate with insulin resistance and type 2 diabetes (Wang et al. 2011), however it is unclear if tyrosine plays a mechanistic role in this process. To summarize, although the precise role of most metabolites from our study is unclear, they do seem to associate with the cardiometabolic sequelae of excess visceral and liver fat which makes their association with lipid accumulation in these locations plausible.

Our study was limited by a few factors. First, although our sample size was larger than some previous metabolomics studies on body fat distribution, we lacked the power to properly test for interaction by sex or to develop and validate diagnostic quantitative models for lipid accumulation. Because of this, the lack of statistically significant interactions by sex should not be interpreted as evidence of absence of differences in metabolite associations between men and women. Second, a large proportion of our female participants was peri- or postmenopausal. As menopause is associated with substantial metabolic changes including changes in visceral fat mass (Polotsky and Polotsky 2010), our results may not be extrapolated to a younger population. Third, the use of this specific metabolomics platform limited the selection of metabolites which we could investigate. However, an advantage of this platform is that it has been extensively validated and consists of a set of analytically and biologically well-defined metabolites. Fourth, we were not able to replicate our findings as there was no other readily available cohort with both Biocrates and ^1^H-MRS measurements of liver fat. Nevertheless, we showed that Biocrates metabolites have the potential to function as biomarkers of liver and visceral fat and encourage the use of replication cohorts in more extensive studies that aim to develop prediction models using metabolites. Strengths of our study are that we performed our analyses in a lipid or glucose lowering drug naive sample from a well phenotyped cohort with direct, quantitative measures of visceral and liver fat. Because of this, we could account for overall obesity by including total body fat in our models and assess which metabolites were specifically associated with quantitative measures of visceral fat or hepatic triglyceride content even after accounting for commonly used approximation methods.

In conclusion, we demonstrated specific associations of metabolites with visceral fat and hepatic triglyceride content that may be useful in diagnostic approaches of lipid accumulation in both locations. We encourage future studies to include enough participants to develop and validate diagnostic models containing metabolite data and to compare their diagnostic performance against currently used approximation methods. To determine if such approximation methods could also be useful for monitoring changes in visceral and liver fat content, we also recommend performing prospective studies with repeated measurements.

## Electronic supplementary material

Below is the link to the electronic supplementary material.

Supplementary material 1 (DOCX 19 kb)

Supplementary material 2 (PDF 382 kb)

Supplementary material 3 (PDF 382 kb)

Supplementary material 4 (DOCX 24 kb)

Supplementary material 5 (PDF 211 kb)

Supplementary material 6 (DOCX 89 kb)
