# Supplementary material for: Metabolomics: a search for biomarkers of visceral fat and liver fat content
Source: Metabolomics. 2019 Oct 5;15(10):139. doi: 10.1007/s11306-019-1599-x (PMC6778586; doi:10.1007/s11306-019-1599-x)
Supplement: Supplementary file 1 — Supplementary material 1 (DOCX 19 kb) [file 11306_2019_1599_MOESM1_ESM.docx]

**Online Resource 1**

**Article title:** Metabolomics: a search for biomarkers of visceral and liver fat content

**Journal name:** Metabolomics

**Author names:**

Sebastiaan Boone^1^, Dennis Mook-Kanamori^1,2^, Frits Rosendaal^1^, Martin den Heijer^1,8^, Hildo Lamb^3^, Albert de Roos^3^, Saskia le Cessie^1,4^, Ko Willems van Dijk^5,6,7^, Renée de Mutsert^1^

**Affiliations:**

1 Department of Clinical Epidemiology, Leiden University Medical Center, Leiden, the Netherlands

2 Department of Public Health and Primary Care, Leiden University Medical Center, Leiden, the Netherlands

3 Department of Radiology, Leiden University Medical Center, Leiden, the Netherlands

4 Department of Biomedical Data Sciences, section Medical Statistics and Bioinformatics, Leiden University Medical Center, Leiden, the Netherlands

5 Department of Endocrinology, Leiden University Medical Center, Leiden, the Netherlands

6 Einthoven Laboratory for Experimental Vascular Medicine, Leiden University Medical Center, Leiden, the Netherlands

7 Human Genetics, Leiden University Medical Center, Leiden, the Netherlands

8 Endocrinology, VU Medical Centre, Amsterdam, The Netherlands

**Corresponding author:**

S.C. Boone, MD, PhD candidate

Leiden University Medical Center (LUMC), Department of Clinical Epidemiology

PO Box 9600, 2300 RC Leiden

Department C7-P, Postal Zone C7-Q

Fax: +31 (0)71 526 6994

Tel: +31 (0)71 526 4037

Email: s.c.boone@lumc.nl

ORCID: 0000-0002-2411-0699

**Online Resource 1** Overview of measured metabolite variables

| **Acylcarnitines** | Lyso PC a C18:2 |
| --- | --- |
| C0 | Lyso PC a C20:3 |
| C2 | Lyso PC a C20:4 |
| C3 | Lyso PC a C24:0 |
| C3-DC (C4-OH) | Lyso PC a C26:0 |
| C3-OH | Lyso PC a C26:1 |
| C3:1 | Lyso PC a C28:0 |
| C4 | Lyso PC a C28:1 |
| C4:1 | **Diacyl phosphatidylcholines** |
| C5 | PC aa C24:0 |
| C5-DC (C6-OH) | PC aa C26:0 |
| C5-M-DC | PC aa C28:1 |
| C5-OH (C3-DC-M) | PC aa C30:0 |
| C5:1 | PC aa C30:2 |
| C5:1-DC | PC aa C32:0 |
| C6 (C4:1-DC) | PC aa C32:1 |
| C6:1 | PC aa C32:2 |
| C7-DC | PC aa C32:3 |
| C8 | PC aa C34:1 |
| C8:1 | PC aa C34:2 |
| C9 | PC aa C34:3 |
| C10 | PC aa C34:4 |
| C10:1 | PC aa C36:0 |
| C10:2 | PC aa C36:1 |
| C12 | PC aa C36:2 |
| C12-DC | PC aa C36:3 |
| C12:1 | PC aa C36:4 |
| C14 | PC aa C36:5 |
| C14:1 | PC aa C36:6 |
| C14:1-OH | PC aa C38:0 |
| C14:2 | PC aa C38:1 |
| C14:2-OH | PC aa C38:3 |
| C16 | PC aa C38:4 |
| C16-OH | PC aa C38:5 |
| C16:1 | PC aa C38:6 |
| C16:1-OH | PC aa C40:1 |
| C16:2 | PC aa C40:2 |
| C16:2-OH | PC aa C40:3 |
| C18 | PC aa C40:4 |
| C18:1 | PC aa C40:5 |
| C18:1-OH | PC aa C40:6 |
| C18:2 | PC aa C42:0 |
| **Lysophosphatidylcholines** | PC aa C42:1 |
| Lyso PC a C6:0 | PC aa C42:2 |
| Lyso PC a C14:0 | PC aa C42:4 |
| Lyso PC a C16:0 | PC aa C42:5 |
| Lyso PC a C16:1 | PC aa C42:6 |
| Lyso PC a C17:0 | **Acyl-alkyl phosphatidylcholines** |
| Lyso PC a C18:0 | PC ae C30:0 |
| Lyso PC a C18:1 | PC ae C30:1 |
| **Acyl-alkyl phosphatidylcholines (cont.)** | SM C24:0 |
| PC ae C30:2 | SM C24:1 |
| PC ae C32:1 | SM C26:0 |
| PC ae C32:2 | SM C26:1 |
| PC ae C34:0 | **Amino acids** |
| PC ae C34:1 | Arginine |
| PC ae C34:2 | Glutamine |
| PC ae C34:3 | Glycine |
| PC ae C36:0 | Histidine |
| PC ae C36:1 | Methionine |
| PC ae C36:2 | Ornithine |
| PC ae C36:3 | Phenylalanine |
| PC ae C36:4 | Proline |
| PC ae C36:5 | Serine |
| PC ae C38:0 | Threonine |
| PC ae C38:1 | Tryptophan |
| PC ae C38:2 | Tyrosine |
| PC ae C38:3 | Valine |
| PC ae C38:4 | (Iso)Leucine |
| PC ae C38:5 | **Hexoses** |
| PC ae C38:6 | Hexoses |
| PC ae C40:0 | **Aggregate measures** |
| PC ae C40:1 | (C2 + C3) / C0 |
| PC ae C40:2 | Aromatic amino acids (AAA) |
| PC ae C40:3 | C2 / C0 |
| PC ae C40:4 | (C16 + C18) / C0 |
| PC ae C40:5 | MUFA(PC) |
| PC ae C40:6 | MUFA(PC) / SFA(PC) |
| PC ae C42:0 | Ornithine / Arginine |
| PC ae C42:1 | PUFA(PC) |
| PC ae C42:2 | PUFA(PC) / MUFA(PC) |
| PC ae C42:3 | PUFA(PC) / SFA(PC) |
| PC ae C42:4 | SFA(PC) |
| PC ae C42:5 | Total PC + Total SM |
| PC ae C44:3 | Total AC / C0 |
| PC ae C44:4 | Total AC-DC / Total AC |
| PC ae C44:5 | Total AC-OH / Total AC |
| PC ae C44:6 | Total lysoPC |
| **Sphingomyelins** | Total lysoPC / Total PC |
| SM (OH) C14:1 | Total PC |
| SM (OH) C16:1 | Total diacyl PC |
| SM (OH) C22:1 | Total acyl-alkyl PC |
| SM (OH) C22:2 | Total SM |
| SM (OH) C24:1 | Total SM / (Total SM + Total PC) |
| SM C16:0 | Total SM / Total PC |
| SM C16:1 | Total SM-non OH |
| SM C18:0 | Total SM-OH |
| SM C18:1 | Total SM-OH / Total SM-non OH |
| SM C20:2 | Tyrosine / Phenylalanine |
| SM C22:3 |  |
