# Supplementary material for: Metabolomics: a search for biomarkers of visceral fat and liver fat content
Source: Metabolomics. 2019 Oct 5;15(10):139. doi: 10.1007/s11306-019-1599-x (PMC6778586; doi:10.1007/s11306-019-1599-x)

Online Resource 3

Article title: Metabolomics: a search for biomarkers of visceral and liver fat content

Journal name: Metabolomics

Author names:
Sebastiaan Boone[1], Dennis Mook-Kanamori[1,2], Frits Rosendaal[1], Martin den Heijer[1,8], Hildo Lamb[3], Albert de Roos[3], Saskia le Cessie[1,4], Ko Willems van Dijk[5,6,7], Renée de Mutsert[1]

Affiliations:
1 Department of Clinical Epidemiology, Leiden University Medical Center, Leiden, the Netherlands
2 Department of Public Health and Primary Care, Leiden University Medical Center, Leiden, the Netherlands
3 Department of Radiology, Leiden University Medical Center, Leiden, the Netherlands
4 Department of Biomedical Data Sciences, section Medical Statistics and Bioinformatics, Leiden University Medical Center, Leiden, the Netherlands
5 Department of Endocrinology, Leiden University Medical Center, Leiden, the Netherlands
6 Einthoven Laboratory for Experimental Vascular Medicine, Leiden University Medical Center, Leiden, the Netherlands
7 Human Genetics, Leiden University Medical Center, Leiden, the Netherlands
8 Endocrinology, VU Medical Centre, Amsterdam, The Netherlands

Corresponding author:
S.C. Boone, MD, PhD candidate
Leiden University Medical Center (LUMC), Department of Clinical Epidemiology
PO Box 9600, 2300 RC Leiden
Department C7-P, Postal Zone C7-Q
Fax: +31 (0)71 526 6994
Tel: +31 (0)71 526 4037
Email: s.c.boone@lumc.nl
ORCID: 0000-0002-2411-0699

Online Resource 3 Heatmaps of regression coefficients for the analyses on HTGC in (a) the crude model, (b) adjusted for age, sex and total body fat, (c) adjusted additionally for waist circumference and fasting concentrations of triglycerides, HDL and cholesterol. Outcomes represent a relative increase in HTGC per SD of metabolite concentration. Green: positive associations, red: negative associations. Hashtag symbols (#): significant after FDR adjustment

Online Resource 3a

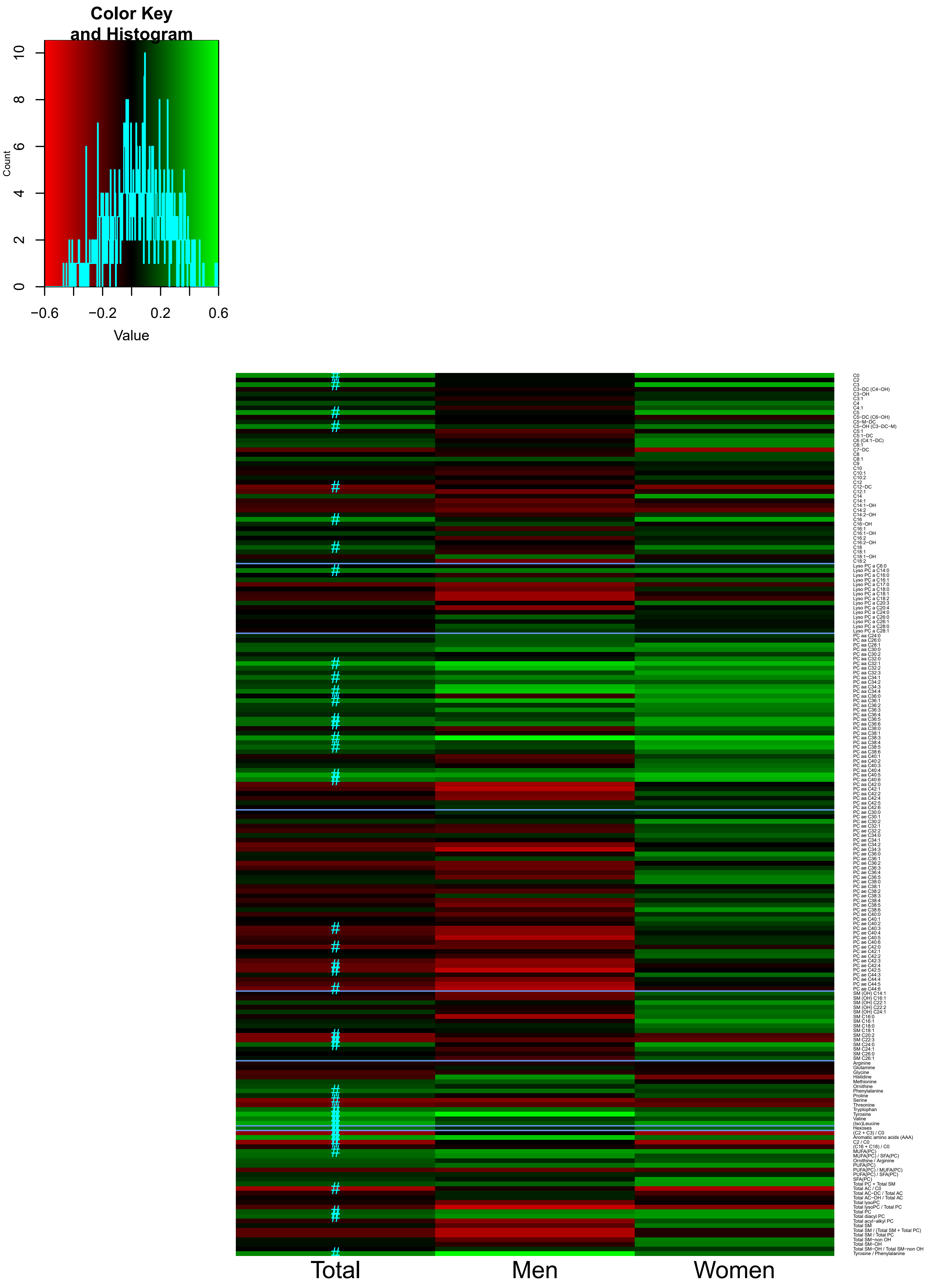

Online Resource 3b

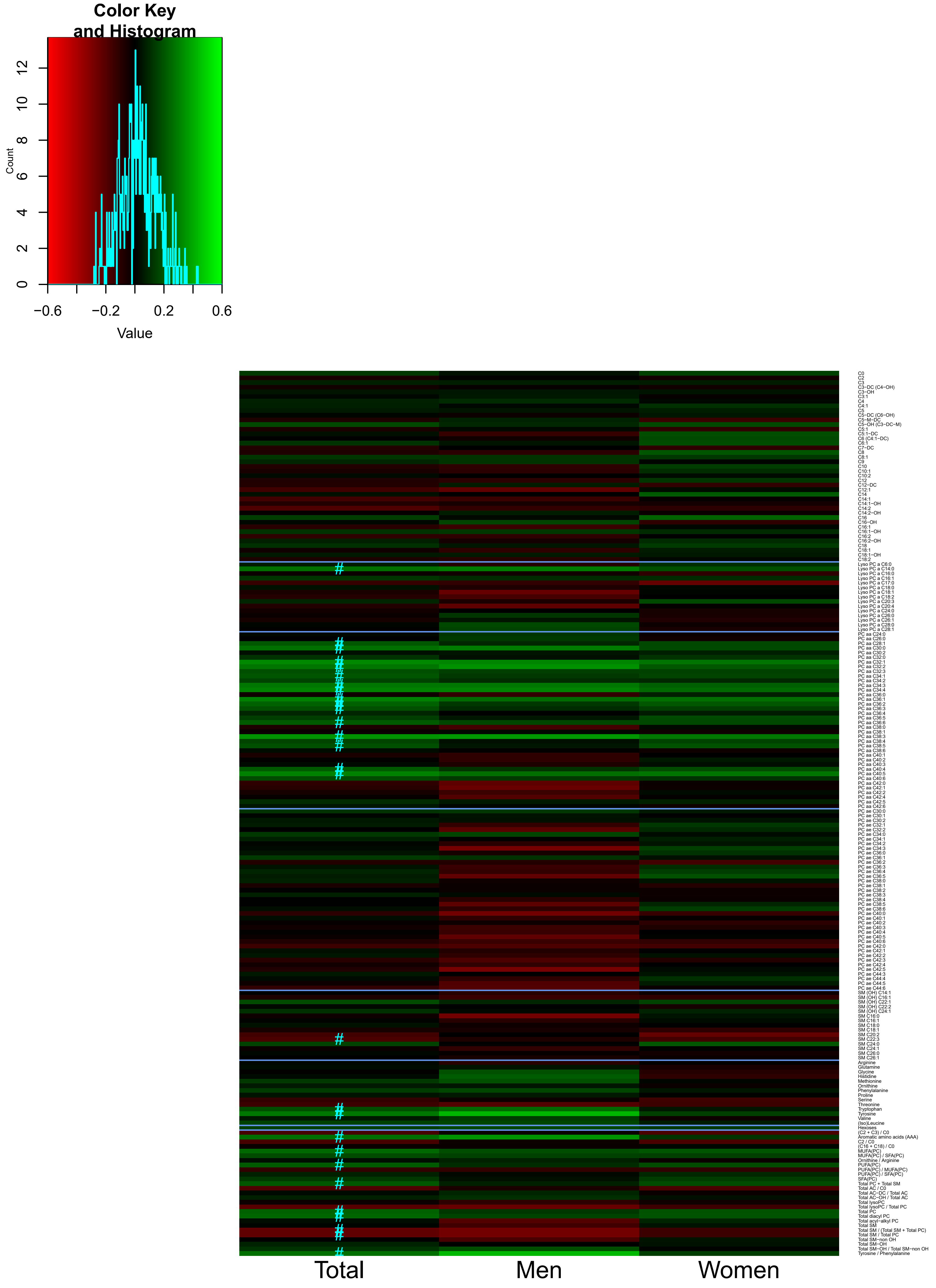

Online Resource 3c

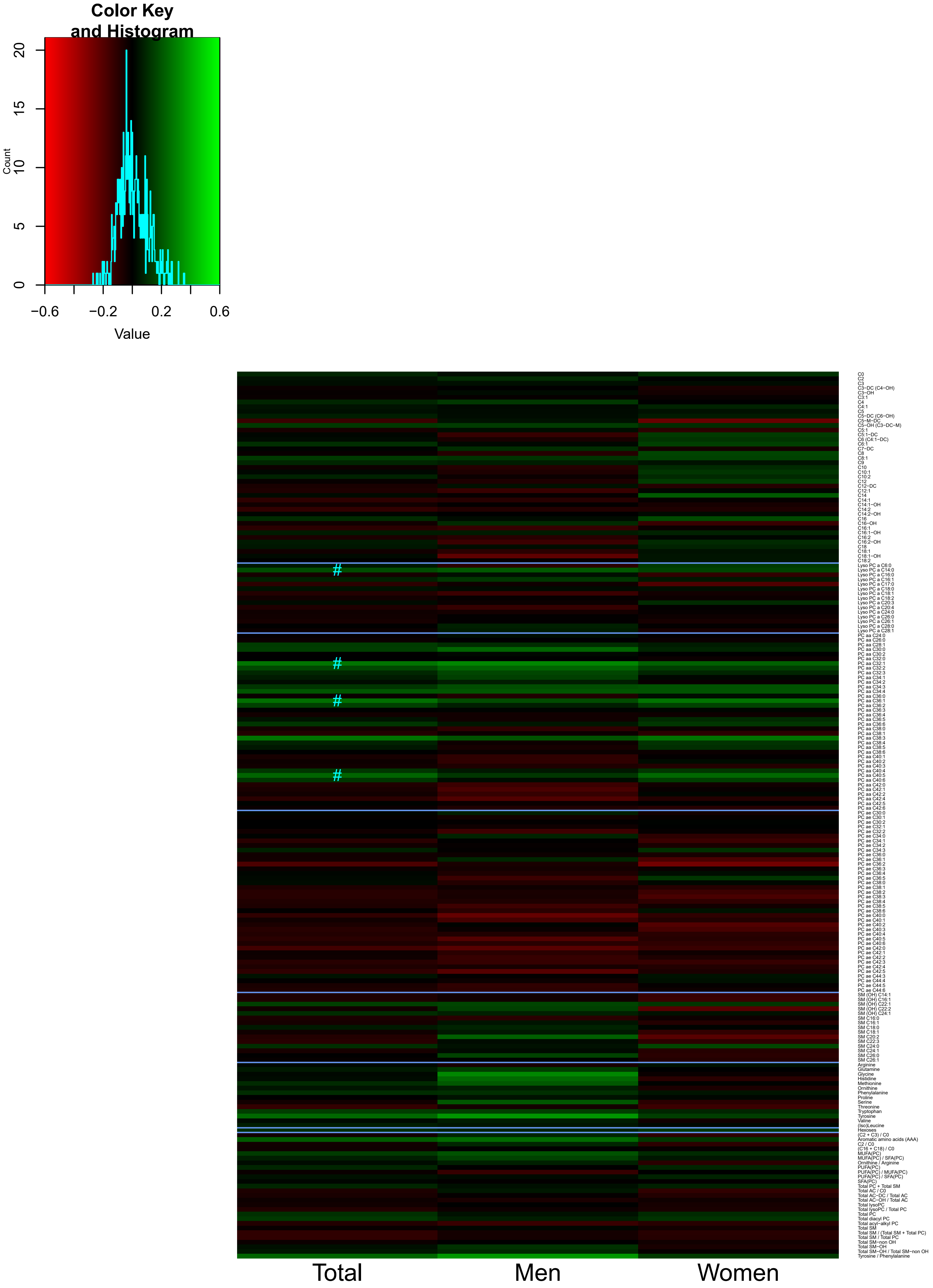

Supplement: Supplementary file 3 — Supplementary material 3 (PDF 382 kb) [file 11306_2019_1599_MOESM3_ESM.pdf]
