# Supplementary material for: Metabolomics: a search for biomarkers of visceral fat and liver fat content
Source: Metabolomics. 2019 Oct 5;15(10):139. doi: 10.1007/s11306-019-1599-x (PMC6778586; doi:10.1007/s11306-019-1599-x)
Supplement: Supplementary file 4 — Supplementary material 4 (DOCX 24 kb) [file 11306_2019_1599_MOESM4_ESM.docx]

**Online Resource 4**

**Article title:** Metabolomics: a search for biomarkers of visceral and liver fat content

**Journal name:** Metabolomics

**Author names:**

Sebastiaan Boone^1^, Dennis Mook-Kanamori^1,2^, Frits Rosendaal^1^, Martin den Heijer^1,8^, Hildo Lamb^3^, Albert de Roos^3^, Saskia le Cessie^1,4^, Ko Willems van Dijk^5,6,7^, Renée de Mutsert^1^

**Affiliations:**

1 Department of Clinical Epidemiology, Leiden University Medical Center, Leiden, the Netherlands

2 Department of Public Health and Primary Care, Leiden University Medical Center, Leiden, the Netherlands

3 Department of Radiology, Leiden University Medical Center, Leiden, the Netherlands

4 Department of Biomedical Data Sciences, section Medical Statistics and Bioinformatics, Leiden University Medical Center, Leiden, the Netherlands

5 Department of Endocrinology, Leiden University Medical Center, Leiden, the Netherlands

6 Einthoven Laboratory for Experimental Vascular Medicine, Leiden University Medical Center, Leiden, the Netherlands

7 Human Genetics, Leiden University Medical Center, Leiden, the Netherlands

8 Endocrinology, VU Medical Centre, Amsterdam, The Netherlands

**Corresponding author:**

S.C. Boone, MD, PhD candidate

Leiden University Medical Center (LUMC), Department of Clinical Epidemiology

PO Box 9600, 2300 RC Leiden

Department C7-P, Postal Zone C7-Q

Fax: +31 (0)71 526 6994

Tel: +31 (0)71 526 4037

Email: s.c.boone@lumc.nl

ORCID: 0000-0002-2411-0699

**Online Resource 4.** Metabolites associated with visceral adipose tissue (N = 174) or hepatic triglyceride content (N = 149) after false discovery rate correction in model 1 (crude)

|  | **Visceral adipose tissue** | | | | | |
| --- | --- | --- | --- | --- | --- | --- |
|  | Total |  | Men |  | Women |  |
|  | (N = 174) |  | (N = 84) |  | (N = 90) |  |
|  | Estimate (95% CI) | P-value | Estimate (95% CI) | P-value | Estimate (95% CI) | P-value |
| **Acylcarnitines** |  |  |  |  |  |  |
| C3 | 10.6 (3.5; 17.6) | 3.67E-03 | -4.3 (-13.8; 5.2) | 3.71E-01 | 12.7 (3.9; 21.5) | 5.14E-03 |
| C5 | 18.3 (8.2; 28.4) | 4.58E-04 | -0.9 (-15.8; 13.9) | 9.01E-01 | 21.4 (10.1; 32.7) | 3.08E-04 |
| C7-DC | -10.5 (-18.6; -2.4) | 1.17E-02 | -10.7 (-22.0; 0.7) | 6.58E-02 | -9.8 (-18.0; -1.7) | 1.86E-02 |
| C12-DC | -11.2 (-19.7; -2.7) | 9.94E-03 | -9.7 (-27.8; 8.4) | 2.88E-01 | -5.6 (-11.5; 0.4) | 6.83E-02 |
| C12:1 | -12.5 (-19.6; -5.5) | 5.95E-04 | -16.1 (-28.1; -4.0) | 9.77E-03 | -5.9 (-11.8; 0.0) | 5.01E-02 |
| C14:1-OH | -12.4 (-19.8; -5.0) | 1.11E-03 | -18.3 (-34.1; -2.5) | 2.35E-02 | -5.5 (-11.3; 0.3) | 6.22E-02 |
| C14:2 | -11.8 (-18.5; -5.1) | 6.62E-04 | -17.6 (-26.6; -8.7) | 1.88E-04 | -7.4 (-14.2; -0.5) | 3.52E-02 |
| **Lysophosphatidylcholines** |  |  |  |  |  |  |
| LysoPC a C17:0 | -17.2 (-25.7; -8.6) | 1.18E-04 | -23.5 (-35.8; -11.2) | 2.81E-04 | -10.0 (-17.0; -3.0) | 5.54E-03 |
| LysoPC a C18:0 | -12.6 (-22.2; -3.1) | 9.79E-03 | -26.3 (-42.7; -9.9) | 2.03E-03 | -4.6 (-11.6; 2.4) | 1.99E-01 |
| LysoPC a C18:1 | -13.5 (-22.4; -4.6) | 3.18E-03 | -22.0 (-34.7; -9.2) | 9.59E-04 | -11.1 (-19.8; -2.4) | 1.34E-02 |
| LysoPC a C18:2 | -16.1 (-24.3; -7.8) | 1.75E-04 | -33.3 (-44.7; -21.9) | 1.12E-07 | -13.5 (-22.1; -5.0) | 2.28E-03 |
| **Diacyl phosphatidylcholines** |  |  |  |  |  |  |
| PC aa C42:0 | -13.7 (-22.4; -5.0) | 2.21E-03 | -19.7 (-35.5; -3.8) | 1.55E-02 | -2.7 (-10.1; 4.7) | 4.66E-01 |
| PC aa C42:1 | -11.1 (-19.1; -3.0) | 7.41E-03 | -14.9 (-33.0; 3.2) | 1.05E-01 | -3.1 (-10.2; 4.0) | 3.83E-01 |
| **Acyl-alkyl phosphatidylcholines** |  |  |  |  |  |  |
| PC ae C30:0 | -13.7 (-22.6; -4.9) | 2.51E-03 | -14.2 (-29.4; 1.1) | 6.79E-02 | -1.7 (-11.1; 7.6) | 7.13E-01 |
| PC ae C32:1 | -14.5 (-23.3; -5.7) | 1.33E-03 | -14.7 (-31.6; 2.3) | 8.90E-02 | -3.3 (-11.2; 4.5) | 4.03E-01 |
| PC ae C32:2 | -15.1 (-23.3; -6.8) | 3.95E-04 | -10.5 (-29.7; 8.7) | 2.81E-01 | -0.7 (-9.7; 8.2) | 8.73E-01 |
| PC ae C34:1 | -14.4 (-21.7; -7.2) | 1.32E-04 | -6.2 (-23.8; 11.4) | 4.85E-01 | -3.4 (-11.8; 5.0) | 4.20E-01 |
| PC ae C34:2 | -22.7 (-32.1; -13.3) | 4.15E-06 | -25.6 (-46.9; -4.2) | 1.97E-02 | -7.6 (-17.8; 2.6) | 1.41E-01 |
| PC ae C34:3 | -20.6 (-30.9; -10.4) | 1.07E-04 | -25.2 (-53.2; 2.9) | 7.80E-02 | -8.0 (-17.7; 1.7) | 1.04E-01 |
| PC ae C36:2 | -18.4 (-26.1; -10.7) | 5.34E-06 | -14.8 (-30.0; 0.3) | 5.43E-02 | -6.2 (-15.0; 2.6) | 1.64E-01 |
| PC ae C36:3 | -19.3 (-28.9; -9.8) | 9.79E-05 | -19.5 (-41.9; 2.9) | 8.76E-02 | -4.1 (-14.4; 6.1) | 4.25E-01 |
| PC ae C38:2 | -19.4 (-27.3; -11.5) | 2.47E-06 | -19.0 (-33.7; -4.2) | 1.23E-02 | -5.2 (-14.1; 3.7) | 2.51E-01 |
| PC ae C38:3 | -10.8 (-18.9; -2.8) | 8.84E-03 | 1.6 (-16.7; 19.9) | 8.63E-01 | 1.7 (-6.3; 9.8) | 6.68E-01 |
| PC ae C40:3 | -19.1 (-26.9; -11.4) | 2.28E-06 | -27.4 (-48.4; -6.3) | 1.15E-02 | -1.9 (-10.5; 6.7) | 6.64E-01 |
| PC ae C40:4 | -17.1 (-24.6; -9.7) | 1.15E-05 | -20.1 (-34.5; -5.6) | 7.14E-03 | -6.0 (-12.9; 0.8) | 8.19E-02 |
| PC ae C42:3 | -14.1 (-23.8; -4.4) | 4.73E-03 | -17.1 (-39.6; 5.4) | 1.35E-01 | 0.8 (-7.4; 9.0) | 8.47E-01 |
| PC ae C42:4 | -21.7 (-29.3; -14.1) | 6.26E-08 | -26.4 (-36.2; -16.6) | 7.28E-07 | -10.4 (-18.0; -2.7) | 8.26E-03 |
| PC ae C42:5 | -15.2 (-23.5; -7.0) | 3.43E-04 | -18.9 (-35.2; -2.6) | 2.40E-02 | -5.3 (-12.3; 1.8) | 1.40E-01 |
| PC ae C44:4 | -15.8 (-23.3; -8.3) | 5.06E-05 | -19.3 (-31.2; -7.3) | 1.90E-03 | -7.2 (-14.0; -0.4) | 3.74E-02 |
| PC ae C44:6 | -16.6 (-25.0; -8.3) | 1.26E-04 | -24.5 (-38.3; -10.7) | 6.82E-04 | -7.9 (-14.8; -1.1) | 2.42E-02 |
| **Sphingomyelins** |  |  |  |  |  |  |
| SM (OH) C16:1 | -10.5 (-18.6; -2.4) | 1.17E-02 | -17.3 (-32.5; -2.1) | 2.64E-02 | 2.8 (-4.4; 9.9) | 4.46E-01 |
| SM (OH) C22:2 | -12.3 (-20.3; -4.4) | 2.48E-03 | -6.9 (-29.4; 15.6) | 5.44E-01 | 2.2 (-6.1; 10.4) | 6.03E-01 |
| SM C16:0 | -13.2 (-22.1; -4.4) | 3.68E-03 | -19.0 (-37.3; -0.8) | 4.15E-02 | 0.4 (-7.6; 8.5) | 9.16E-01 |
| SM C20:2 | -15.8 (-23.5; -8.1) | 8.23E-05 | -10.7 (-31.6; 10.2) | 3.10E-01 | -0.1 (-8.0; 7.7) | 9.78E-01 |
| **Amino acids** |  |  |  |  |  |  |
| Glycine | -18.9 (-25.9; -11.9) | 3.35E-07 | -45.0 (-74.6; -15.4) | 3.32E-03 | -7.9 (-12.3; -3.4) | 6.58E-04 |
| Serine | -16.0 (-25.1; -6.9) | 6.75E-04 | -19.9 (-43.0; 3.1) | 8.89E-02 | -7.6 (-13.8; -1.5) | 1.57E-02 |
| Valine | 19.7 (12.0; 27.4) | 1.15E-06 | 2.6 (-14.4; 19.5) | 7.65E-01 | 14.8 (6.5; 23.2) | 6.61E-04 |
| (Iso)leucine | 23.6 (16.9; 30.3) | 5.92E-11 | 7.1 (-6.5; 20.6) | 3.03E-01 | 22.8 (11.8; 33.7) | 8.08E-05 |
| **Hexoses** |  |  |  |  |  |  |
| Hexose | 14.1 (5.1; 23.1) | 2.42E-03 | 7.4 (-10.9; 25.7) | 4.23E-01 | 10.3 (2.6; 18.0) | 9.23E-03 |
| **Aggregate measures** |  |  |  |  |  |  |
| (C2 + C3) / C0 | -11.7 (-19.2; -4.2) | 2.43E-03 | -1.3 (-24.2; 21.7) | 9.13E-01 | -6.6 (-12.4; -0.9) | 2.47E-02 |
| C2 / C0 | -11.9 (-19.4; -4.4) | 2.03E-03 | -1.0 (-24.0; 22.0) | 9.30E-01 | -6.7 (-12.5; -1.0) | 2.25E-02 |
| Total AC / C0 | -13.0 (-20.4; -5.5) | 7.57E-04 | -5.9 (-28.1; 16.4) | 6.01E-01 | -7.0 (-12.9; -1.1) | 2.13E-02 |
| Total lysoPC | -12.4 (-21.5; -3.4) | 7.29E-03 | -25.8 (-38.6; -13.1) | 1.26E-04 | -9.4 (-17.6; -1.2) | 2.44E-02 |
| Total lysoPC / Total PC | -11.9 (-19.9; -3.9) | 3.67E-03 | -40.6 (-53.0; -28.3) | 5.04E-09 | -15.1 (-21.0; -9.3) | 1.81E-06 |
| Total acyl-alkyl PC | -14.5 (-23.6; -5.4) | 2.00E-03 | -11.8 (-32.9; 9.4) | 2.72E-01 | -1.1 (-9.7; 7.5) | 7.96E-01 |
| **(Cont.)** |  |  |  |  |  |  |
|  | **Hepatic triglyceride content** | | | | | |
|  | Total |  | Men |  | Women |  |
|  | (N = 149) |  | (N = 70) |  | (N = 79) |  |
|  | Estimate (95% CI) | P-value | Estimate (95% CI) | P-value | Estimate (95% CI) | P-value |
| **Acylcarnitines** |  |  |  |  |  |  |
| C0 | 1.39 (1.17; 1.65) | 2.90E-04 | 1.01 (0.77; 1.32) | 9.40E-01 | 1.50 (1.22; 1.84) | 1.98E-04 |
| C3 | 1.35 (1.15; 1.58) | 2.61E-04 | 1.01 (0.82; 1.25) | 9.15E-01 | 1.52 (1.20; 1.92) | 6.55E-04 |
| C5 | 1.41 (1.16; 1.71) | 7.22E-04 | 1.02 (0.75; 1.40) | 8.82E-01 | 1.48 (1.21; 1.82) | 2.39E-04 |
| C5-OH (C3-DC-M) | 1.35 (1.15; 1.59) | 2.85E-04 | 1.10 (0.88; 1.38) | 3.75E-01 | 1.33 (1.03; 1.71) | 3.12E-02 |
| C12-DC | 0.78 (0.66; 0.92) | 3.72E-03 | 0.99 (0.76; 1.29) | 9.47E-01 | 0.75 (0.62; 0.92) | 4.96E-03 |
| C16 | 1.38 (1.20; 1.59) | 1.22E-05 | 1.05 (0.88; 1.25) | 5.78E-01 | 1.48 (1.24; 1.76) | 3.04E-05 |
| C18 | 1.23 (1.05; 1.44) | 9.43E-03 | 0.93 (0.77; 1.12) | 4.38E-01 | 1.34 (1.10; 1.64) | 4.95E-03 |
| **Lysophosphatidylcholines** |  |  |  |  |  |  |
| LysoPC a C14:0 | 1.32 (1.12; 1.55) | 8.62E-04 | 1.33 (1.11; 1.58) | 2.44E-03 | 1.34 (1.09; 1.65) | 5.89E-03 |
| **Diacyl phosphatidylcholines** |  |  |  |  |  |  |
| PC aa C32:1 | 1.45 (1.25; 1.69) | 3.02E-06 | 1.65 (1.38; 1.97) | 5.06E-07 | 1.54 (1.28; 1.85) | 1.18E-05 |
| PC aa C34:1 | 1.26 (1.08; 1.48) | 4.05E-03 | 1.35 (1.10; 1.67) | 5.78E-03 | 1.36 (1.15; 1.61) | 4.76E-04 |
| PC aa C34:4 | 1.31 (1.14; 1.50) | 2.42E-04 | 1.60 (1.24; 2.05) | 3.86E-04 | 1.49 (1.28; 1.72) | 1.00E-06 |
| PC aa C36:1 | 1.30 (1.09; 1.55) | 3.33E-03 | 1.52 (1.11; 2.08) | 1.06E-02 | 1.45 (1.22; 1.72) | 4.64E-05 |
| PC aa C36:5 | 1.28 (1.08; 1.52) | 4.81E-03 | 1.16 (0.92; 1.46) | 2.02E-01 | 1.46 (1.18; 1.81) | 5.81E-04 |
| PC aa C36:6 | 1.28 (1.08; 1.53) | 4.64E-03 | 1.33 (1.07; 1.65) | 1.06E-02 | 1.46 (1.22; 1.76) | 8.72E-05 |
| PC aa C38:3 | 1.39 (1.22; 1.60) | 2.92E-06 | 1.81 (1.47; 2.21) | 1.80E-07 | 1.64 (1.44; 1.87) | 1.05E-10 |
| PC aa C38:5 | 1.25 (1.06; 1.48) | 9.24E-03 | 1.17 (0.93; 1.47) | 1.82E-01 | 1.48 (1.24; 1.76) | 4.07E-05 |
| PC aa C40:5 | 1.45 (1.22; 1.72) | 4.15E-05 | 1.44 (1.17; 1.76) | 6.38E-04 | 1.55 (1.29; 1.86) | 9.18E-06 |
| PC aa C40:6 | 1.32 (1.13; 1.55) | 4.71E-04 | 1.27 (1.05; 1.52) | 1.38E-02 | 1.50 (1.28; 1.76) | 2.47E-06 |
| **Acyl-alkyl phosphatidylcholines** |  |  |  |  |  |  |
| PC ae C40:3 | 0.82 (0.71; 0.95) | 6.47E-03 | 0.73 (0.56; 0.96) | 2.57E-02 | 1.10 (0.91; 1.32) | 3.24E-01 |
| PC ae C42:0 | 0.79 (0.68; 0.92) | 2.68E-03 | 0.81 (0.61; 1.07) | 1.36E-01 | 0.92 (0.77; 1.12) | 4.07E-01 |
| PC ae C42:4 | 0.79 (0.68; 0.92) | 2.07E-03 | 0.73 (0.61; 0.88) | 9.24E-04 | 0.95 (0.79; 1.14) | 5.78E-01 |
| PC ae C42:5 | 0.79 (0.67; 0.93) | 5.71E-03 | 0.64 (0.52; 0.79) | 6.03E-05 | 1.00 (0.82; 1.23) | 9.79E-01 |
| PC ae C44:6 | 0.78 (0.66; 0.92) | 3.77E-03 | 0.66 (0.53; 0.82) | 3.79E-04 | 0.92 (0.74; 1.14) | 4.46E-01 |
| **Sphingomyelines** |  |  |  |  |  |  |
| SM C20:2 | 0.75 (0.64; 0.89) | 9.14E-04 | 0.94 (0.72; 1.23) | 6.43E-01 | 0.85 (0.67; 1.08) | 1.80E-01 |
| SM C22:3 | 0.76 (0.65; 0.89) | 5.42E-04 | 0.81 (0.60; 1.10) | 1.75E-01 | 0.81 (0.68; 0.95) | 1.14E-02 |
| SM C24:0 | 1.25 (1.09; 1.44) | 1.98E-03 | 0.98 (0.75; 1.27) | 8.49E-01 | 1.45 (1.23; 1.70) | 1.73E-05 |
| **Amino acids** |  |  |  |  |  |  |
| Phenylalanine | 1.27 (1.06; 1.53) | 9.89E-03 | 1.29 (1.03; 1.62) | 2.85E-02 | 1.13 (0.89; 1.43) | 3.05E-01 |
| Serine | 0.75 (0.65; 0.87) | 2.21E-04 | 0.74 (0.50; 1.10) | 1.33E-01 | 0.83 (0.70; 1.00) | 4.54E-02 |
| Tryptophan | 1.28 (1.09; 1.51) | 3.04E-03 | 1.29 (1.03; 1.63) | 2.82E-02 | 1.18 (0.96; 1.45) | 1.14E-01 |
| Tyrosine | 1.49 (1.14; 1.94) | 3.60E-03 | 1.78 (1.47; 2.16) | 6.96E-08 | 1.33 (0.96; 1.84) | 8.37E-02 |
| Valine | 1.34 (1.13; 1.58) | 7.05E-04 | 1.13 (0.85; 1.51) | 3.87E-01 | 1.19 (0.92; 1.53) | 1.84E-01 |
| (Iso)leucine | 1.47 (1.27; 1.71) | 6.08E-07 | 1.21 (0.97; 1.52) | 9.06E-02 | 1.45 (1.05; 1.98) | 2.27E-02 |
| **Hexoses** |  |  |  |  |  |  |
| Hexose | 1.30 (1.11; 1.53) | 1.33E-03 | 1.10 (0.81; 1.50) | 5.35E-01 | 1.27 (1.03; 1.57) | 2.86E-02 |
| **Aggregate measures** |  |  |  |  |  |  |
| (C2 + C3) / C0 | 0.72 (0.56; 0.92) | 8.27E-03 | 1.00 (0.66; 1.50) | 9.92E-01 | 0.70 (0.56; 0.86) | 1.39E-03 |
| Aromatic amino acids (AAA) | 1.45 (1.17; 1.79) | 6.79E-04 | 1.60 (1.27; 2.01) | 1.29E-04 | 1.29 (0.98; 1.69) | 6.96E-02 |
| C2 / C0 | 0.71 (0.56; 0.91) | 7.00E-03 | 1.00 (0.66; 1.50) | 9.87E-01 | 0.69 (0.56; 0.86) | 1.34E-03 |
| MUFA(PC) / Total PC | 1.29 (1.09; 1.52) | 2.65E-03 | 1.44 (1.14; 1.81) | 2.95E-03 | 1.41 (1.19; 1.66) | 9.39E-05 |
| Total AC / C0 | 0.69 (0.54; 0.88) | 3.40E-03 | 0.95 (0.61; 1.46) | 8.01E-01 | 0.68 (0.54; 0.85) | 8.54E-04 |
| Total PC | 1.22 (1.05; 1.42) | 8.66E-03 | 1.35 (1.01; 1.82) | 4.55E-02 | 1.42 (1.22; 1.66) | 1.38E-05 |
| Total diacyl PC | 1.25 (1.08; 1.45) | 2.68E-03 | 1.41 (1.06; 1.88) | 1.82E-02 | 1.44 (1.24; 1.67) | 5.85E-06 |
| Tyrosine / Phenylalanine | 1.38 (1.09; 1.75) | 7.44E-03 | 1.79 (1.49; 2.15) | 1.77E-08 | 1.31 (1.02; 1.70) | 3.73E-02 |

Metabolites that reached the FDR adjusted p-value in the total group are summarized. The reported numbers represent regression outcomes (95% CI) from model 1 (crude) without adjusting for any other variables, expressed as the change in cm2 per SD of metabolite concentration for VAT and the relative increase in HTGC per SD for HTGC.
