# Supplementary material for: Metabolomics: a search for biomarkers of visceral fat and liver fat content
Source: Metabolomics. 2019 Oct 5;15(10):139. doi: 10.1007/s11306-019-1599-x (PMC6778586; doi:10.1007/s11306-019-1599-x)
Supplement: Supplementary file 5 — Supplementary material 5 (PDF 211 kb) [file 11306_2019_1599_MOESM5_ESM.pdf]

Online Resource 5

Article title: Metabolomics: a search for biomarkers of visceral and liver fat content

Journal name: Metabolomics

Author names:
Sebastiaan Boone[1], Dennis Mook-Kanamori[1,2], Frits Rosendaal[1], Martin den Heijer[1,8], Hildo Lamb[3], Albert de Roos[3], Saskia le Cessie[1,4], Ko Willems van Dijk[5,6,7], Renée de Mutsert[1]

Affiliations:
1 Department of Clinical Epidemiology, Leiden University Medical Center, Leiden, the Netherlands
2 Department of Public Health and Primary Care, Leiden University Medical Center, Leiden, the Netherlands
3 Department of Radiology, Leiden University Medical Center, Leiden, the Netherlands
4 Department of Biomedical Data Sciences, section Medical Statistics and Bioinformatics, Leiden University Medical Center, Leiden, the Netherlands
5 Department of Endocrinology, Leiden University Medical Center, Leiden, the Netherlands
6 Einthoven Laboratory for Experimental Vascular Medicine, Leiden University Medical Center, Leiden, the Netherlands
7 Human Genetics, Leiden University Medical Center, Leiden, the Netherlands
8 Endocrinology, VU Medical Centre, Amsterdam, The Netherlands

Corresponding author:
S.C. Boone, MD, PhD candidate
Leiden University Medical Center (LUMC), Department of Clinical Epidemiology
PO Box 9600, 2300 RC Leiden
Department C7-P, Postal Zone C7-Q
Fax: +31 (0)71 526 6994
Tel: +31 (0)71 526 4037
Email: s.c.boone@lumc.nl
ORCID: 0000-0002-2411-0699

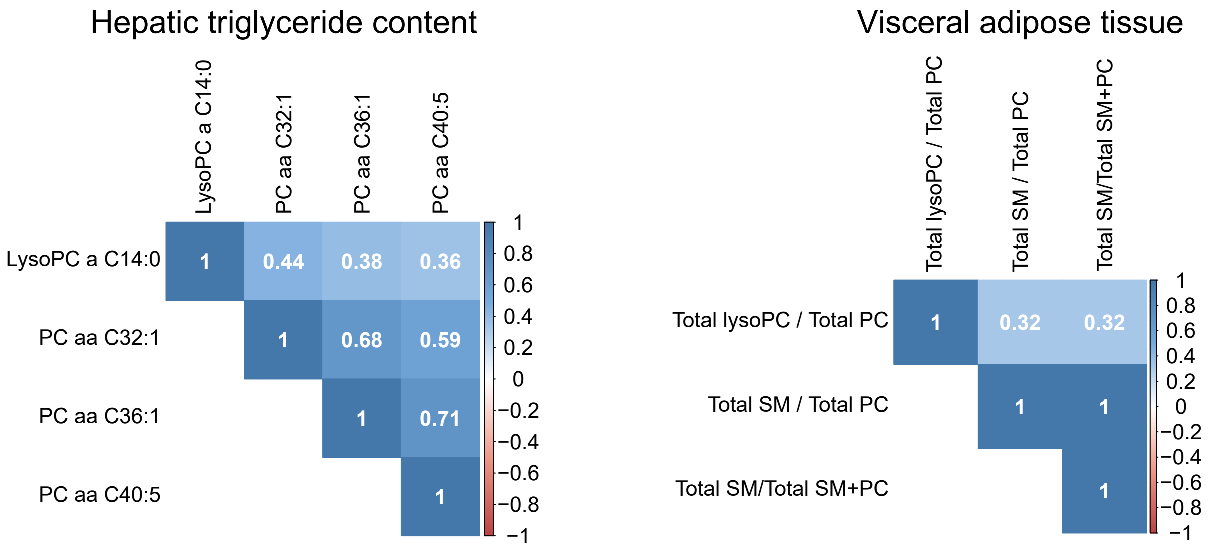

Online resource 5 Correlation plots of metabolites that were significantly associated with either hepatic triglyceride content or visceral adipose tissue in the complete sample in model 3 (adjusted for age, sex, total body fat percentage, waist circumference and fasting concentrations of triglycerides, HDL-cholesterol, and total cholesterol).
