# Supplementary material for: Metabolomics: a search for biomarkers of visceral fat and liver fat content
Source: Metabolomics. 2019 Oct 5;15(10):139. doi: 10.1007/s11306-019-1599-x (PMC6778586; doi:10.1007/s11306-019-1599-x)
Supplement: Supplementary file 6 — Supplementary material 6 (DOCX 89 kb) [file 11306_2019_1599_MOESM6_ESM.docx]

**Online Resource 6**

**Article title:** Metabolomics: a search for biomarkers of visceral and liver fat content

**Journal name:** Metabolomics

**Author names:**

Sebastiaan Boone^1^, Dennis Mook-Kanamori^1,2^, Frits Rosendaal^1^, Martin den Heijer^1,8^, Hildo Lamb^3^, Albert de Roos^3^, Saskia le Cessie^1,4^, Ko Willems van Dijk^5,6,7^, Renée de Mutsert^1^

**Affiliations:**

1 Department of Clinical Epidemiology, Leiden University Medical Center, Leiden, the Netherlands

2 Department of Public Health and Primary Care, Leiden University Medical Center, Leiden, the Netherlands

3 Department of Radiology, Leiden University Medical Center, Leiden, the Netherlands

4 Department of Biomedical Data Sciences, section Medical Statistics and Bioinformatics, Leiden University Medical Center, Leiden, the Netherlands

5 Department of Endocrinology, Leiden University Medical Center, Leiden, the Netherlands

6 Einthoven Laboratory for Experimental Vascular Medicine, Leiden University Medical Center, Leiden, the Netherlands

7 Human Genetics, Leiden University Medical Center, Leiden, the Netherlands

8 Endocrinology, VU Medical Centre, Amsterdam, The Netherlands

**Corresponding author:**

S.C. Boone, MD, PhD candidate

Leiden University Medical Center (LUMC), Department of Clinical Epidemiology

PO Box 9600, 2300 RC Leiden

Department C7-P, Postal Zone C7-Q

Fax: +31 (0)71 526 6994

Tel: +31 (0)71 526 4037

Email: s.c.boone@lumc.nl

ORCID: 0000-0002-2411-0699

**Online Resource 6**

Tables containing all associations from model 1 (crude), model 2 (adjusted for age, sex and total body fat percentage) and model 3 (adjusted for age, sex, total body fat, waist circumference and fasting serum concentrations of triglycerides, HDL cholesterol and total cholesterol). The reported numbers represent regression outcomes (95% CI) expressed as the difference in VAT (cm^2^) per SD of metabolite concentration and the relative increase in HTGC per SD of metabolite concentration. Metabolites that were significantly associated with visceral fat or hepatic triglyceride content after false discovery rate correction are marked with *. The associations listed in these tables are summarized in the figures of Online Resources 2 and 3.

**Model 1 (crude)**

|  | **Visceral adipose tissue** | | | | | |
| --- | --- | --- | --- | --- | --- | --- |
|  | Total |  | Men |  | Women |  |
|  | (N = 174) |  | (N = 84) |  | (N = 90) |  |
|  | Estimate (95% CI) | P-value | Estimate (95% CI) | P-value | Estimate (95% CI) | P-value |
| **Acylcarnitines** |  |  |  |  |  |  |
| C0 | 9.3 (1.1; 17.5) | 2.61E-02 | -2.7 (-16.1; 10.7) | 6.91E-01 | 9.1 (1.5; 16.7) | 2.01E-02 |
| C2 | -6.3 (-15.9; 3.3) | 1.96E-01 | -2.1 (-23.2; 18.9) | 8.43E-01 | -3.0 (-9.6; 3.5) | 3.57E-01 |
| C3 | 10.6 (3.5; 17.6)* | 3.67E-03 | -4.3 (-13.8; 5.2) | 3.71E-01 | 12.7 (3.9; 21.5) | 5.14E-03 |
| C3-DC (C4-OH) | -1.3 (-9.6; 7.0) | 7.65E-01 | 3.6 (-14.6; 21.9) | 6.92E-01 | -1.6 (-7.2; 4.0) | 5.72E-01 |
| C3-OH | 5.5 (-2.9; 14.0) | 1.96E-01 | 7.7 (-6.6; 21.9) | 2.87E-01 | 0.1 (-8.3; 8.6) | 9.75E-01 |
| C3:1 | 0.6 (-7.8; 8.9) | 8.95E-01 | -11.0 (-29.0; 7.0) | 2.27E-01 | 4.9 (-2.4; 12.2) | 1.88E-01 |
| C4 | 1.4 (-4.8; 7.5) | 6.66E-01 | -6.5 (-13.0; -0.1) | 4.66E-02 | 8.1 (-4.1; 20.3) | 1.88E-01 |
| C4:1 | -3.3 (-11.7; 5.1) | 4.37E-01 | -8.6 (-23.2; 6.0) | 2.45E-01 | 3.6 (-4.8; 12.0) | 4.00E-01 |
| C5 | 18.3 (8.2; 28.4)* | 4.58E-04 | -0.9 (-15.8; 13.9) | 9.01E-01 | 21.4 (10.1; 32.7) | 3.08E-04 |
| C5-DC (C6-OH) | -5.4 (-14.5; 3.7) | 2.46E-01 | -4.9 (-21.0; 11.2) | 5.47E-01 | -5.4 (-15.5; 4.7) | 2.89E-01 |
| C5-M-DC | 1.1 (-8.6; 10.9) | 8.18E-01 | -9.8 (-26.0; 6.4) | 2.31E-01 | 5.7 (-1.5; 12.9) | 1.17E-01 |
| C5-OH (C3-DC-M) | 7.0 (-1.6; 15.6) | 1.08E-01 | -1.7 (-17.5; 14.2) | 8.35E-01 | 1.2 (-7.3; 9.7) | 7.79E-01 |
| C5:1 | -3.5 (-11.1; 4.0) | 3.58E-01 | -12.4 (-26.7; 2.0) | 9.02E-02 | 2.4 (-7.1; 12.0) | 6.11E-01 |
| C5:1-DC | -5.0 (-14.1; 4.1) | 2.82E-01 | -6.4 (-20.4; 7.7) | 3.69E-01 | -1.8 (-9.1; 5.5) | 6.30E-01 |
| C6 (C4:1-DC) | -1.9 (-11.1; 7.4) | 6.90E-01 | -4.7 (-19.2; 9.9) | 5.25E-01 | 0.5 (-6.6; 7.6) | 8.83E-01 |
| C6:1 | -2.3 (-11.3; 6.6) | 6.09E-01 | -7.5 (-18.6; 3.6) | 1.83E-01 | 7.1 (-1.6; 15.8) | 1.08E-01 |
| C7-DC | -10.5 (-18.6; -2.4)* | 1.17E-02 | -10.7 (-22.0; 0.7) | 6.58E-02 | -9.8 (-18.0; -1.7) | 1.86E-02 |
| C8 | -6.4 (-16.0; 3.1) | 1.84E-01 | -6.9 (-18.5; 4.6) | 2.34E-01 | -4.5 (-15.2; 6.3) | 4.13E-01 |
| C8:1 | 13.3 (2.8; 23.7) | 1.30E-02 | 16.8 (2.3; 31.4) | 2.41E-02 | 10.6 (2.5; 18.6) | 1.04E-02 |
| C9 | -9.1 (-17.6; -0.5) | 3.72E-02 | -17.6 (-29.1; -6.1) | 3.23E-03 | -0.4 (-8.5; 7.8) | 9.32E-01 |
| C10 | -8.3 (-16.6; -0.1) | 4.85E-02 | -7.1 (-16.8; 2.5) | 1.45E-01 | -7.3 (-17.7; 3.1) | 1.68E-01 |
| C10:1 | -10.7 (-19.2; -2.3) | 1.30E-02 | -13.0 (-22.4; -3.7) | 7.02E-03 | -5.6 (-15.1; 3.9) | 2.43E-01 |
| C10:2 | -2.5 (-11.7; 6.7) | 5.92E-01 | -2.1 (-18.4; 14.1) | 7.94E-01 | 3.2 (-4.6; 11.1) | 4.13E-01 |
| C12 | -9.1 (-16.4; -1.8) | 1.53E-02 | -11.8 (-22.0; -1.5) | 2.51E-02 | -5.0 (-12.1; 2.0) | 1.60E-01 |
| C12-DC | -11.2 (-19.7; -2.7)* | 9.94E-03 | -9.7 (-27.8; 8.4) | 2.88E-01 | -5.6 (-11.5; 0.4) | 6.83E-02 |
| C12:1 | -12.5 (-19.6; -5.5)* | 5.95E-04 | -16.1 (-28.1; -4.0) | 9.77E-03 | -5.9 (-11.8; 0.0) | 5.01E-02 |
| C14 | -4.9 (-14.1; 4.2) | 2.90E-01 | -13.8 (-27.1; -0.5) | 4.27E-02 | 1.1 (-8.1; 10.2) | 8.16E-01 |
| C14:1 | -7.8 (-14.7; -0.8) | 2.83E-02 | -11.7 (-26.9; 3.5) | 1.29E-01 | -2.3 (-7.8; 3.1) | 3.93E-01 |
| C14:1-OH | -12.4 (-19.8; -5.0)* | 1.11E-03 | -18.3 (-34.1; -2.5) | 2.35E-02 | -5.5 (-11.3; 0.3) | 6.22E-02 |
| C14:2 | -11.8 (-18.5; -5.1)* | 6.62E-04 | -17.6 (-26.6; -8.7) | 1.88E-04 | -7.4 (-14.2; -0.5) | 3.52E-02 |
| C14:2-OH | -2.1 (-11.4; 7.2) | 6.58E-01 | -17.2 (-30.9; -3.5) | 1.45E-02 | 5.3 (-1.6; 12.2) | 1.29E-01 |
| C16 | 5.2 (-3.9; 14.4) | 2.61E-01 | -4.4 (-16.7; 8.0) | 4.83E-01 | 2.3 (-5.6; 10.2) | 5.61E-01 |
| C16-OH | 6.9 (-1.4; 15.3) | 1.03E-01 | 5.7 (-7.5; 18.9) | 3.94E-01 | 3.0 (-4.4; 10.3) | 4.26E-01 |
| C16:1 | -2.5 (-11.7; 6.7) | 5.94E-01 | -1.8 (-17.8; 14.1) | 8.19E-01 | -0.8 (-7.9; 6.4) | 8.31E-01 |
| C16:1-OH | -0.9 (-10.3; 8.4) | 8.45E-01 | -3.4 (-18.6; 11.9) | 6.61E-01 | 1.1 (-6.3; 8.6) | 7.62E-01 |
| C16:2 | -5.1 (-13.5; 3.3) | 2.34E-01 | -12.3 (-26.5; 1.8) | 8.58E-02 | -0.3 (-7.4; 6.8) | 9.37E-01 |
| C16:2-OH | -4.8 (-14.4; 4.7) | 3.21E-01 | -12.5 (-27.4; 2.3) | 9.60E-02 | -1.6 (-8.4; 5.1) | 6.29E-01 |
| C18 | -0.8 (-10.6; 9.1) | 8.78E-01 | -11.3 (-26.3; 3.7) | 1.37E-01 | 0.7 (-8.1; 9.5) | 8.74E-01 |
| C18:1 | 4.3 (-5.0; 13.7) | 3.58E-01 | 3.5 (-10.6; 17.7) | 6.22E-01 | 1.2 (-6.3; 8.6) | 7.55E-01 |
| C18:1-OH | -6.1 (-13.4; 1.1) | 9.67E-02 | 23.3 (-0.2; 46.8) | 5.22E-02 | -3.6 (-9.3; 2.0) | 2.00E-01 |
| C18:2 | -1.9 (-11.7; 7.9) | 7.03E-01 | -14.8 (-28.1; -1.5) | 2.97E-02 | -3.7 (-12.7; 5.2) | 4.08E-01 |
| **Lysophosphatidylcholines** |  |  |  |  |  |  |
| Lyso PC a C6:0 | -0.2 (-10.4; 10.0) | 9.70E-01 | 8.1 (-8.3; 24.6) | 3.28E-01 | 0.1 (-9.2; 9.3) | 9.86E-01 |
| Lyso PC a C14:0 | -1.5 (-10.8; 7.7) | 7.43E-01 | -1.3 (-14.7; 12.0) | 8.45E-01 | 0.7 (-8.7; 10.1) | 8.83E-01 |
| Lyso PC a C16:0 | -8.0 (-17.6; 1.5) | 9.94E-02 | -16.4 (-29.6; -3.3) | 1.50E-02 | -8.5 (-16.6; -0.5) | 3.86E-02 |
| Lyso PC a C16:1 | -1.5 (-10.8; 7.7) | 7.46E-01 | 2.4 (-11.2; 15.9) | 7.29E-01 | 0.2 (-7.2; 7.6) | 9.60E-01 |
| Lyso PC a C17:0 | -17.2 (-25.7; -8.6)* | 1.18E-04 | -23.5 (-35.8; -11.2) | 2.81E-04 | -10.0 (-17.0; -3.0) | 5.54E-03 |
| Lyso PC a C18:0 | -12.6 (-22.2; -3.1)* | 9.79E-03 | -26.3 (-42.7; -9.9) | 2.03E-03 | -4.6 (-11.6; 2.4) | 1.99E-01 |
| Lyso PC a C18:1 | -13.5 (-22.4; -4.6)* | 3.18E-03 | -22.0 (-34.7; -9.2) | 9.59E-04 | -11.1 (-19.8; -2.4) | 1.34E-02 |
| Lyso PC a C18:2 | -16.1 (-24.3; -7.8)* | 1.75E-04 | -33.3 (-44.7; -21.9) | 1.12E-07 | -13.5 (-22.1; -5.0) | 2.28E-03 |
| Lyso PC a C20:3 | -4.1 (-13.0; 4.9) | 3.71E-01 | -10.8 (-22.7; 1.0) | 7.14E-02 | -2.4 (-11.4; 6.5) | 5.88E-01 |
| Lyso PC a C20:4 | -0.8 (-9.0; 7.5) | 8.55E-01 | -12.7 (-23.9; -1.6) | 2.51E-02 | -2.5 (-11.4; 6.3) | 5.67E-01 |
| Lyso PC a C24:0 | 4.1 (-4.8; 13.0) | 3.64E-01 | 5.1 (-14.8; 24.9) | 6.12E-01 | 9.9 (3.4; 16.5) | 3.40E-03 |
| Lyso PC a C26:0 | 5.4 (-2.5; 13.3) | 1.78E-01 | 9.1 (-3.4; 21.5) | 1.52E-01 | 9.5 (2.1; 16.8) | 1.23E-02 |
| Lyso PC a C26:1 | 4.1 (-4.5; 12.7) | 3.45E-01 | 6.2 (-9.5; 21.8) | 4.34E-01 | 8.9 (1.7; 16.0) | 1.56E-02 |
| Lyso PC a C28:0 | 1.2 (-7.1; 9.6) | 7.69E-01 | 3.1 (-12.4; 18.5) | 6.93E-01 | 8.5 (0.7; 16.3) | 3.25E-02 |
| Lyso PC a C28:1 | -3.6 (-12.4; 5.2) | 4.20E-01 | 1.9 (-16.0; 19.8) | 8.33E-01 | 7.0 (-0.9; 14.9) | 8.21E-02 |
| **Diacyl phosphatidylcholines** |  |  |  |  |  |  |
| PC aa C24:0 | 6.6 (-1.2; 14.4) | 9.48E-02 | 10.8 (-5.6; 27.2) | 1.95E-01 | 11.3 (5.1; 17.5) | 4.60E-04 |
| PC aa C26:0 | 3.8 (-4.1; 11.8) | 3.44E-01 | 9.6 (-1.8; 21.1) | 9.80E-02 | 6.9 (-0.7; 14.5) | 7.65E-02 |
| PC aa C28:1 | 0.9 (-8.0; 9.8) | 8.46E-01 | 14.1 (-7.3; 35.6) | 1.94E-01 | 5.7 (-2.0; 13.4) | 1.46E-01 |
| PC aa C30:0 | -6.8 (-15.5; 2.0) | 1.29E-01 | 0.1 (-14.9; 15.2) | 9.87E-01 | -1.4 (-10.2; 7.5) | 7.59E-01 |
| PC aa C30:2 | -6.0 (-13.5; 1.5) | 1.14E-01 | -5.9 (-19.6; 7.7) | 3.88E-01 | -1.7 (-9.8; 6.4) | 6.83E-01 |
| PC aa C32:0 | -4.3 (-13.1; 4.4) | 3.31E-01 | 3.0 (-9.6; 15.6) | 6.32E-01 | -2.0 (-10.7; 6.6) | 6.40E-01 |
| PC aa C32:1 | 6.5 (-2.9; 16.0) | 1.74E-01 | 18.2 (3.6; 32.7) | 1.50E-02 | 5.8 (-1.3; 12.9) | 1.11E-01 |
| PC aa C32:2 | -3.5 (-11.5; 4.6) | 3.96E-01 | 6.6 (-11.7; 24.9) | 4.78E-01 | 3.3 (-4.2; 10.8) | 3.88E-01 |
| PC aa C32:3 | -6.7 (-14.7; 1.4) | 1.04E-01 | 2.3 (-23.2; 27.8) | 8.60E-01 | 7.9 (0.5; 15.3) | 3.67E-02 |
| PC aa C34:1 | 3.4 (-5.8; 12.7) | 4.62E-01 | 19.3 (5.2; 33.4) | 7.93E-03 | 3.3 (-3.4; 10.0) | 3.29E-01 |
| PC aa C34:2 | -1.4 (-9.5; 6.7) | 7.37E-01 | 8.7 (-8.8; 26.1) | 3.27E-01 | 2.0 (-5.0; 9.1) | 5.69E-01 |
| PC aa C34:3 | -4.2 (-11.3; 2.9) | 2.46E-01 | 22.8 (5.7; 39.8) | 9.49E-03 | 2.8 (-4.6; 10.3) | 4.51E-01 |
| PC aa C34:4 | 4.2 (-4.3; 12.6) | 3.30E-01 | 22.9 (7.1; 38.7) | 4.98E-03 | 9.0 (2.4; 15.6) | 8.13E-03 |
| PC aa C36:0 | -3.8 (-13.6; 6.0) | 4.44E-01 | -4.6 (-21.6; 12.4) | 5.93E-01 | 7.6 (-1.7; 16.9) | 1.07E-01 |
| PC aa C36:1 | -1.9 (-10.2; 6.4) | 6.56E-01 | 17.7 (0.2; 35.2) | 4.78E-02 | 3.3 (-4.2; 10.8) | 3.80E-01 |
| PC aa C36:2 | -6.8 (-15.0; 1.5) | 1.06E-01 | 0.0 (-19.8; 19.7) | 9.99E-01 | 2.2 (-5.4; 9.8) | 5.60E-01 |
| PC aa C36:3 | -3.5 (-11.3; 4.3) | 3.77E-01 | 16.5 (0.9; 32.1) | 3.87E-02 | 3.2 (-4.0; 10.5) | 3.78E-01 |
| PC aa C36:4 | 4.6 (-3.8; 13.0) | 2.83E-01 | 19.4 (5.3; 33.4) | 7.50E-03 | 6.6 (-0.6; 13.7) | 7.16E-02 |
| PC aa C36:5 | 8.8 (0.5; 17.0) | 3.85E-02 | 11.2 (-1.0; 23.4) | 7.05E-02 | 9.7 (1.2; 18.2) | 2.61E-02 |
| PC aa C36:6 | 4.5 (-3.0; 12.0) | 2.39E-01 | 12.4 (-2.9; 27.7) | 1.10E-01 | 11.1 (5.6; 16.7) | 1.38E-04 |
| PC aa C38:0 | -5.3 (-14.3; 3.7) | 2.49E-01 | -6.1 (-23.5; 11.3) | 4.89E-01 | 7.7 (-0.2; 15.5) | 5.53E-02 |
| PC aa C38:1 | 3.0 (-6.7; 12.6) | 5.44E-01 | 0.7 (-19.5; 20.9) | 9.45E-01 | 7.4 (-0.5; 15.2) | 6.51E-02 |
| PC aa C38:3 | 4.3 (-4.4; 13.0) | 3.32E-01 | 26.3 (10.8; 41.9) | 1.17E-03 | 11.2 (4.4; 18.0) | 1.57E-03 |
| PC aa C38:4 | 6.1 (-2.8; 15.0) | 1.80E-01 | 20.2 (3.0; 37.3) | 2.16E-02 | 11.0 (3.7; 18.3) | 3.46E-03 |
| PC aa C38:5 | 5.8 (-2.5; 14.2) | 1.71E-01 | 16.8 (5.2; 28.5) | 5.08E-03 | 8.6 (0.6; 16.6) | 3.49E-02 |
| PC aa C38:6 | 6.5 (-1.3; 14.3) | 1.03E-01 | 7.1 (-7.6; 21.8) | 3.38E-01 | 12.4 (5.3; 19.4) | 7.38E-04 |
| PC aa C40:1 | -7.2 (-17.0; 2.5) | 1.44E-01 | -12.8 (-28.5; 2.9) | 1.09E-01 | 5.4 (-3.7; 14.6) | 2.43E-01 |
| PC aa C40:2 | -3.1 (-12.4; 6.2) | 5.09E-01 | -6.1 (-23.5; 11.3) | 4.85E-01 | 6.3 (-2.4; 15.0) | 1.53E-01 |
| PC aa C40:3 | -5.3 (-14.7; 4.1) | 2.65E-01 | -5.2 (-22.0; 11.6) | 5.39E-01 | 4.1 (-5.1; 13.3) | 3.75E-01 |
| PC aa C40:4 | 0.4 (-8.9; 9.6) | 9.36E-01 | 14.2 (-2.4; 30.7) | 9.22E-02 | 1.7 (-6.2; 9.7) | 6.65E-01 |
| PC aa C40:5 | 8.4 (-0.8; 17.6) | 7.25E-02 | 17.2 (3.2; 31.1) | 1.65E-02 | 8.8 (1.0; 16.5) | 2.71E-02 |
| PC aa C40:6 | 8.9 (0.9; 16.8) | 2.99E-02 | 9.9 (-4.9; 24.8) | 1.88E-01 | 15.7 (9.0; 22.3) | 1.15E-05 |
| PC aa C42:0 | -13.7 (-22.4; -5.0)* | 2.21E-03 | -19.7 (-35.5; -3.8) | 1.55E-02 | -2.7 (-10.1; 4.7) | 4.66E-01 |
| PC aa C42:1 | -11.1 (-19.1; -3.0)* | 7.41E-03 | -14.9 (-33.0; 3.2) | 1.05E-01 | -3.1 (-10.2; 4.0) | 3.83E-01 |
| PC aa C42:2 | -3.6 (-13.0; 5.7) | 4.47E-01 | -8.5 (-26.7; 9.6) | 3.52E-01 | 5.0 (-3.0; 13.1) | 2.16E-01 |
| PC aa C42:4 | -8.5 (-17.7; 0.7) | 7.06E-02 | -4.6 (-27.6; 18.5) | 6.94E-01 | -3.2 (-10.7; 4.3) | 3.94E-01 |
| PC aa C42:5 | 0.4 (-8.7; 9.5) | 9.31E-01 | 6.9 (-7.8; 21.5) | 3.55E-01 | 2.9 (-5.9; 11.6) | 5.16E-01 |
| PC aa C42:6 | -6.5 (-14.5; 1.5) | 1.13E-01 | 8.2 (-7.6; 24.1) | 3.04E-01 | -0.3 (-9.3; 8.7) | 9.52E-01 |
| **Acyl-alkyl phosphatidylcholines** |  |  |  |  |  |  |
| PC ae C30:0 | -13.7 (-22.6; -4.9)* | 2.51E-03 | -14.2 (-29.4; 1.1) | 6.79E-02 | -1.7 (-11.1; 7.6) | 7.13E-01 |
| PC ae C30:1 | -6.7 (-17.3; 3.9) | 2.14E-01 | -7.6 (-26.7; 11.5) | 4.29E-01 | 2.1 (-6.5; 10.8) | 6.27E-01 |
| PC ae C30:2 | 2.8 (-5.3; 10.8) | 4.98E-01 | 9.5 (-7.8; 26.7) | 2.78E-01 | 14.4 (7.0; 21.8) | 2.11E-04 |
| PC ae C32:1 | -14.5 (-23.3; -5.7)* | 1.33E-03 | -14.7 (-31.6; 2.3) | 8.90E-02 | -3.3 (-11.2; 4.5) | 4.03E-01 |
| PC ae C32:2 | -15.1 (-23.3; -6.8)* | 3.95E-04 | -10.5 (-29.7; 8.7) | 2.81E-01 | -0.7 (-9.7; 8.2) | 8.73E-01 |
| PC ae C34:0 | -9.6 (-17.8; -1.4) | 2.22E-02 | -7.8 (-22.8; 7.2) | 3.02E-01 | -1.4 (-8.8; 6.0) | 7.13E-01 |
| PC ae C34:1 | -14.4 (-21.7; -7.2)* | 1.32E-04 | -6.2 (-23.8; 11.4) | 4.85E-01 | -3.4 (-11.8; 5.0) | 4.20E-01 |
| PC ae C34:2 | -22.7 (-32.1; -13.3)* | 4.15E-06 | -25.6 (-46.9; -4.2) | 1.97E-02 | -7.6 (-17.8; 2.6) | 1.41E-01 |
| PC ae C34:3 | -20.6 (-30.9; -10.4)* | 1.07E-04 | -25.2 (-53.2; 2.9) | 7.80E-02 | -8.0 (-17.7; 1.7) | 1.04E-01 |
| PC ae C36:0 | -4.3 (-14.4; 5.9) | 4.07E-01 | 3.3 (-16.1; 22.7) | 7.35E-01 | 3.5 (-6.4; 13.4) | 4.81E-01 |
| PC ae C36:1 | -6.5 (-13.7; 0.6) | 7.42E-02 | 9.4 (-10.1; 28.9) | 3.42E-01 | 1.0 (-6.2; 8.3) | 7.78E-01 |
| PC ae C36:2 | -18.4 (-26.1; -10.7)* | 5.34E-06 | -14.8 (-30.0; 0.3) | 5.43E-02 | -6.2 (-15.0; 2.6) | 1.64E-01 |
| PC ae C36:3 | -19.3 (-28.9; -9.8)* | 9.79E-05 | -19.5 (-41.9; 2.9) | 8.76E-02 | -4.1 (-14.4; 6.1) | 4.25E-01 |
| PC ae C36:4 | -5.6 (-15.1; 3.9) | 2.44E-01 | -2.3 (-18.8; 14.2) | 7.83E-01 | 2.5 (-5.6; 10.6) | 5.41E-01 |
| PC ae C36:5 | -2.3 (-12.0; 7.4) | 6.37E-01 | 0.4 (-18.5; 19.2) | 9.69E-01 | 2.5 (-4.9; 10.0) | 5.04E-01 |
| PC ae C38:0 | -2.0 (-9.6; 5.6) | 6.08E-01 | 7.8 (-9.1; 24.6) | 3.61E-01 | 9.2 (3.4; 15.0) | 2.30E-03 |
| PC ae C38:1 | -5.5 (-13.9; 2.9) | 1.97E-01 | -3.8 (-19.3; 11.8) | 6.30E-01 | 3.1 (-6.0; 12.2) | 5.03E-01 |
| PC ae C38:2 | -19.4 (-27.3; -11.5)* | 2.47E-06 | -19.0 (-33.7; -4.2) | 1.23E-02 | -5.2 (-14.1; 3.7) | 2.51E-01 |
| PC ae C38:3 | -10.8 (-18.9; -2.8)* | 8.84E-03 | 1.6 (-16.7; 19.9) | 8.63E-01 | 1.7 (-6.3; 9.8) | 6.68E-01 |
| PC ae C38:4 | -11.0 (-19.7; -2.3) | 1.31E-02 | -5.7 (-23.8; 12.5) | 5.35E-01 | -1.8 (-9.6; 6.0) | 6.50E-01 |
| PC ae C38:5 | -4.8 (-14.5; 4.8) | 3.25E-01 | 0.4 (-16.5; 17.4) | 9.60E-01 | 1.5 (-6.5; 9.4) | 7.11E-01 |
| PC ae C38:6 | -1.3 (-10.6; 8.1) | 7.90E-01 | -1.1 (-19.0; 16.9) | 9.07E-01 | 9.5 (2.3; 16.7) | 1.05E-02 |
| PC ae C40:0 | -5.5 (-13.2; 2.2) | 1.61E-01 | 5.2 (-12.4; 22.8) | 5.58E-01 | 4.8 (-1.9; 11.6) | 1.56E-01 |
| PC ae C40:1 | -5.3 (-14.4; 3.8) | 2.53E-01 | 1.9 (-19.1; 22.8) | 8.59E-01 | 5.4 (-1.5; 12.3) | 1.21E-01 |
| PC ae C40:2 | -5.8 (-14.2; 2.5) | 1.69E-01 | -0.6 (-20.4; 19.2) | 9.53E-01 | 4.2 (-3.1; 11.5) | 2.51E-01 |
| PC ae C40:3 | -19.1 (-26.9; -11.4)* | 2.28E-06 | -27.4 (-48.4; -6.3) | 1.15E-02 | -1.9 (-10.5; 6.7) | 6.64E-01 |
| PC ae C40:4 | -17.1 (-24.6; -9.7)* | 1.15E-05 | -20.1 (-34.5; -5.6) | 7.14E-03 | -6.0 (-12.9; 0.8) | 8.19E-02 |
| PC ae C40:5 | -10.9 (-19.7; -2.2) | 1.40E-02 | -12.1 (-29.9; 5.8) | 1.82E-01 | -2.4 (-9.6; 4.9) | 5.18E-01 |
| PC ae C40:6 | -7.5 (-15.5; 0.6) | 6.98E-02 | -8.8 (-25.4; 7.9) | 2.99E-01 | 3.5 (-3.3; 10.3) | 3.09E-01 |
| PC ae C42:0 | -6.1 (-14.4; 2.3) | 1.52E-01 | 2.4 (-16.9; 21.7) | 8.04E-01 | 3.6 (-3.9; 11.0) | 3.44E-01 |
| PC ae C42:1 | -2.6 (-11.4; 6.1) | 5.54E-01 | 4.0 (-13.6; 21.5) | 6.54E-01 | 8.6 (0.6; 16.5) | 3.48E-02 |
| PC ae C42:2 | -9.2 (-19.0; 0.5) | 6.30E-02 | -9.0 (-29.5; 11.5) | 3.86E-01 | 1.7 (-6.9; 10.2) | 6.98E-01 |
| PC ae C42:3 | -14.1 (-23.8; -4.4)* | 4.73E-03 | -17.1 (-39.6; 5.4) | 1.35E-01 | 0.8 (-7.4; 9.0) | 8.47E-01 |
| PC ae C42:4 | -21.7 (-29.3; -14.1)* | 6.26E-08 | -26.4 (-36.2; -16.6) | 7.28E-07 | -10.4 (-18.0; -2.7) | 8.26E-03 |
| PC ae C42:5 | -15.2 (-23.5; -7.0)* | 3.43E-04 | -18.9 (-35.2; -2.6) | 2.40E-02 | -5.3 (-12.3; 1.8) | 1.40E-01 |
| PC ae C44:3 | -5.2 (-15.3; 4.9) | 3.07E-01 | -6.7 (-30.6; 17.2) | 5.80E-01 | 6.7 (-1.6; 14.9) | 1.11E-01 |
| PC ae C44:4 | -15.8 (-23.3; -8.3)* | 5.06E-05 | -19.3 (-31.2; -7.3) | 1.90E-03 | -7.2 (-14.0; -0.4) | 3.74E-02 |
| PC ae C44:5 | -10.7 (-19.9; -1.5) | 2.30E-02 | -15.0 (-30.9; 0.9) | 6.39E-02 | -3.2 (-10.9; 4.5) | 4.09E-01 |
| PC ae C44:6 | -16.6 (-25.0; -8.3)* | 1.26E-04 | -24.5 (-38.3; -10.7) | 6.82E-04 | -7.9 (-14.8; -1.1) | 2.42E-02 |
| **Sphingomyelins** |  |  |  |  |  |  |
| SM (OH) C14:1 | -9.1 (-17.6; -0.6) | 3.54E-02 | -12.4 (-30.8; 6.0) | 1.83E-01 | 2.9 (-4.9; 10.7) | 4.62E-01 |
| SM (OH) C16:1 | -10.5 (-18.6; -2.4)* | 1.17E-02 | -17.3 (-32.5; -2.1) | 2.64E-02 | 2.8 (-4.4; 9.9) | 4.46E-01 |
| SM (OH) C22:1 | -5.8 (-14.0; 2.4) | 1.67E-01 | -0.2 (-20.8; 20.5) | 9.87E-01 | 3.6 (-3.1; 10.4) | 2.87E-01 |
| SM (OH) C22:2 | -12.3 (-20.3; -4.4)* | 2.48E-03 | -6.9 (-29.4; 15.6) | 5.44E-01 | 2.2 (-6.1; 10.4) | 6.03E-01 |
| SM (OH) C24:1 | -3.8 (-12.2; 4.7) | 3.79E-01 | -3.5 (-23.4; 16.5) | 7.30E-01 | 3.0 (-2.8; 8.8) | 3.10E-01 |
| SM C16:0 | -13.2 (-22.1; -4.4)* | 3.68E-03 | -19.0 (-37.3; -0.8) | 4.15E-02 | 0.4 (-7.6; 8.5) | 9.16E-01 |
| SM C16:1 | -3.6 (-12.0; 4.8) | 4.00E-01 | 3.9 (-17.5; 25.4) | 7.15E-01 | 10.5 (3.1; 17.8) | 5.75E-03 |
| SM C18:0 | -1.5 (-9.9; 6.8) | 7.18E-01 | -1.1 (-19.6; 17.4) | 9.08E-01 | 7.6 (0.1; 15.0) | 4.75E-02 |
| SM C18:1 | -3.8 (-12.1; 4.5) | 3.68E-01 | -1.8 (-20.7; 17.1) | 8.52E-01 | 9.4 (2.1; 16.6) | 1.18E-02 |
| SM C20:2 | -15.8 (-23.5; -8.1)* | 8.23E-05 | -10.7 (-31.6; 10.2) | 3.10E-01 | -0.1 (-8.0; 7.7) | 9.78E-01 |
| SM C22:3 | -8.7 (-17.0; -0.4) | 4.03E-02 | -15.1 (-38.4; 8.3) | 2.03E-01 | -0.7 (-7.7; 6.3) | 8.34E-01 |
| SM C24:0 | 1.2 (-6.8; 9.2) | 7.64E-01 | 3.5 (-12.3; 19.3) | 6.61E-01 | 3.8 (-2.7; 10.3) | 2.52E-01 |
| SM C24:1 | -2.1 (-10.4; 6.2) | 6.20E-01 | 1.9 (-15.5; 19.3) | 8.28E-01 | 5.3 (-1.1; 11.7) | 1.06E-01 |
| SM C26:0 | -3.2 (-12.1; 5.8) | 4.87E-01 | 3.3 (-16.8; 23.4) | 7.48E-01 | 1.5 (-5.7; 8.6) | 6.84E-01 |
| SM C26:1 | -4.8 (-14.1; 4.4) | 3.06E-01 | -15.9 (-35.2; 3.5) | 1.06E-01 | 5.2 (-1.2; 11.6) | 1.07E-01 |
| **Amino acids** |  |  |  |  |  |  |
| Arginine | -8.2 (-17.6; 1.2) | 8.63E-02 | -7.4 (-24.8; 10.0) | 3.98E-01 | -5.6 (-11.7; 0.4) | 6.89E-02 |
| Glutamine | -9.9 (-19.6; -0.2) | 4.66E-02 | -12.5 (-31.1; 6.0) | 1.82E-01 | -5.3 (-12.0; 1.4) | 1.23E-01 |
| Glycine | -18.9 (-25.9; -11.9)* | 3.35E-07 | -45.0 (-74.6; -15.4) | 3.32E-03 | -7.9 (-12.3; -3.4) | 6.58E-04 |
| Histidine | -3.9 (-13.7; 6.0) | 4.37E-01 | 3.9 (-15.9; 23.7) | 6.96E-01 | -0.6 (-7.1; 5.8) | 8.50E-01 |
| Methionine | 3.2 (-5.5; 11.8) | 4.73E-01 | -4.2 (-16.9; 8.5) | 5.12E-01 | 0.1 (-7.0; 7.3) | 9.69E-01 |
| Ornithine | 1.7 (-9.0; 12.5) | 7.52E-01 | -0.1 (-19.2; 19.0) | 9.92E-01 | 1.2 (-6.5; 8.8) | 7.62E-01 |
| Phenylalanine | 10.3 (2.0; 18.6) | 1.53E-02 | 10.6 (-1.0; 22.2) | 7.17E-02 | 3.2 (-4.6; 11.0) | 4.20E-01 |
| Proline | -0.4 (-8.2; 7.5) | 9.26E-01 | -8.9 (-13.2; -4.6) | 9.30E-05 | 1.7 (-13.7; 17.1) | 8.23E-01 |
| Serine | -16.0 (-25.1; -6.9)* | 6.75E-04 | -19.9 (-43.0; 3.1) | 8.89E-02 | -7.6 (-13.8; -1.5) | 1.57E-02 |
| Threonine | -6.2 (-14.8; 2.4) | 1.57E-01 | -12.5 (-31.6; 6.7) | 1.99E-01 | -1.6 (-7.2; 4.1) | 5.77E-01 |
| Tryptophan | 7.0 (-1.3; 15.3) | 9.97E-02 | 7.8 (-5.3; 20.9) | 2.40E-01 | 1.6 (-5.6; 8.9) | 6.56E-01 |
| Tyrosine | 10.5 (1.3; 19.7) | 2.54E-02 | 20.2 (6.0; 34.4) | 5.76E-03 | 4.6 (-2.6; 11.8) | 2.07E-01 |
| Valine | 19.7 (12.0; 27.4)* | 1.15E-06 | 2.6 (-14.4; 19.5) | 7.65E-01 | 14.8 (6.5; 23.2) | 6.61E-04 |
| (Iso)Leucine | 23.6 (16.9; 30.3)* | 5.92E-11 | 7.1 (-6.5; 20.6) | 3.03E-01 | 22.8 (11.8; 33.7) | 8.08E-05 |
| **Hexoses** |  |  |  |  |  |  |
| Hexoses | 14.1 (5.1; 23.1)* | 2.42E-03 | 7.4 (-10.9; 25.7) | 4.23E-01 | 10.3 (2.6; 18.0) | 9.23E-03 |
| **Aggregate measures** |  |  |  |  |  |  |
| (C2 + C3) / C0 | -11.7 (-19.2; -4.2)* | 2.43E-03 | -1.3 (-24.2; 21.7) | 9.13E-01 | -6.6 (-12.4; -0.9) | 2.47E-02 |
| Aromatic amino acids (AAA) | 10.7 (2.1; 19.2) | 1.48E-02 | 16.0 (3.3; 28.8) | 1.45E-02 | 3.9 (-3.5; 11.3) | 2.98E-01 |
| C2 / C0 | -11.9 (-19.4; -4.4)* | 2.03E-03 | -1.0 (-24.0; 22.0) | 9.30E-01 | -6.7 (-12.5; -1.0) | 2.25E-02 |
| (C16 + C18) / C0 | -7.1 (-15.8; 1.6) | 1.11E-01 | -6.9 (-21.0; 7.3) | 3.37E-01 | -5.5 (-13.9; 2.8) | 1.92E-01 |
| MUFA(PC) | 1.9 (-6.9; 10.7) | 6.74E-01 | 19.4 (4.1; 34.6) | 1.33E-02 | 3.6 (-3.5; 10.6) | 3.14E-01 |
| MUFA(PC) / SFA(PC) | 10.7 (0.2; 21.3) | 4.61E-02 | 24.1 (11.4; 36.8) | 3.11E-04 | 2.2 (-5.6; 9.9) | 5.77E-01 |
| Ornithine / Arginine | 8.2 (-2.1; 18.5) | 1.18E-01 | 4.0 (-11.6; 19.5) | 6.15E-01 | 7.1 (-2.7; 16.8) | 1.54E-01 |
| PUFA(PC) | 0.2 (-7.5; 7.9) | 9.60E-01 | 17.4 (2.2; 32.6) | 2.55E-02 | 6.9 (0.7; 13.1) | 3.07E-02 |
| PUFA(PC) / MUFA(PC) | -3.2 (-13.5; 7.0) | 5.35E-01 | -9.6 (-24.1; 4.8) | 1.88E-01 | 1.5 (-7.3; 10.4) | 7.28E-01 |
| PUFA(PC) / SFA(PC) | 9.2 (0.1; 18.3) | 4.69E-02 | 9.9 (-5.2; 25.1) | 1.96E-01 | 6.4 (-3.7; 16.4) | 2.14E-01 |
| SFA(PC) | -6.4 (-15.6; 2.7) | 1.68E-01 | -0.3 (-17.0; 16.4) | 9.71E-01 | 2.4 (-7.0; 11.8) | 6.10E-01 |
| Total PC + Total SM | -1.0 (-8.7; 6.7) | 7.98E-01 | 14.9 (-0.6; 30.4) | 5.92E-02 | 6.2 (-0.3; 12.7) | 6.31E-02 |
| Total AC / C0 | -13.0 (-20.4; -5.5)* | 7.57E-04 | -5.9 (-28.1; 16.4) | 6.01E-01 | -7.0 (-12.9; -1.1) | 2.13E-02 |
| Total AC-DC / Total AC | 0.5 (-9.8; 10.7) | 9.27E-01 | 2.1 (-17.4; 21.5) | 8.34E-01 | -2.3 (-9.0; 4.5) | 5.06E-01 |
| Total AC-OH / Total AC | 4.0 (-6.7; 14.7) | 4.60E-01 | 12.6 (-7.8; 33.0) | 2.23E-01 | -0.4 (-7.0; 6.3) | 9.11E-01 |
| Total lysoPC | -12.4 (-21.5; -3.4)* | 7.29E-03 | -25.8 (-38.6; -13.1) | 1.26E-04 | -9.4 (-17.6; -1.2) | 2.44E-02 |
| Total lysoPC / Total PC | -11.9 (-19.9; -3.9)* | 3.67E-03 | -40.6 (-53.0; -28.3) | 5.04E-09 | -15.1 (-21.0; -9.3) | 1.81E-06 |
| Total PC | 0.4 (-7.3; 8.2) | 9.12E-01 | 18.6 (3.4; 33.8) | 1.73E-02 | 6.3 (-0.1; 12.7) | 5.39E-02 |
| Total diacyl PC | 2.0 (-5.7; 9.7) | 6.10E-01 | 20.8 (5.7; 35.9) | 7.58E-03 | 6.8 (0.6; 13.0) | 3.08E-02 |
| Total acyl-alkyl PC | -14.5 (-23.6; -5.4)* | 2.00E-03 | -11.8 (-32.9; 9.4) | 2.72E-01 | -1.1 (-9.7; 7.5) | 7.96E-01 |
| Total SM | -7.6 (-16.1; 1.0) | 8.31E-02 | -8.1 (-27.5; 11.3) | 4.08E-01 | 4.3 (-2.7; 11.4) | 2.26E-01 |
| Total SM / (Total SM + Total PC) | -10.8 (-19.7; -1.8) | 1.87E-02 | -23.0 (-34.2; -11.7) | 1.13E-04 | -2.9 (-10.4; 4.5) | 4.38E-01 |
| Total SM / Total PC | -10.7 (-19.6; -1.7) | 1.93E-02 | -23.0 (-34.2; -11.7) | 1.10E-04 | -2.8 (-10.3; 4.6) | 4.54E-01 |
| Total SM-non OH | -7.0 (-15.6; 1.7) | 1.13E-01 | -7.7 (-26.3; 10.9) | 4.11E-01 | 4.5 (-2.5; 11.6) | 2.04E-01 |
| Total SM-OH | -9.5 (-17.6; -1.3) | 2.34E-02 | -7.8 (-30.0; 14.4) | 4.85E-01 | 3.2 (-4.1; 10.4) | 3.87E-01 |
| Total SM-OH / Total SM-non OH | -7.6 (-16.5; 1.4) | 9.74E-02 | -0.5 (-17.1; 16.0) | 9.52E-01 | -0.5 (-8.2; 7.1) | 8.91E-01 |
| Tyrosine / Phenylalanine | 5.4 (-4.2; 14.9) | 2.69E-01 | 19.3 (1.8; 36.8) | 3.07E-02 | 3.4 (-3.3; 10.2) | 3.13E-01 |
|  | **Hepatic triglyceride content** | | | | | |
|  | Total |  | Men |  | Women |  |
|  | (N = 149) |  | (N = 70) |  | (N = 79) |  |
|  | Estimate (95% CI) | P-value | Estimate (95% CI) | P-value | Estimate (95% CI) | P-value |
| **Acylcarnitines** |  |  |  |  |  |  |
| C0 | 1.39 (1.17; 1.65)* | 2.90E-04 | 1.01 (0.77; 1.32) | 9.40E-01 | 1.50 (1.22; 1.84) | 1.98E-04 |
| C2 | 0.98 (0.80; 1.20) | 8.56E-01 | 1.02 (0.77; 1.35) | 9.10E-01 | 0.99 (0.81; 1.21) | 9.35E-01 |
| C3 | 1.35 (1.15; 1.58)* | 2.61E-04 | 1.01 (0.82; 1.25) | 9.15E-01 | 1.52 (1.20; 1.92) | 6.55E-04 |
| C3-DC (C4-OH) | 0.95 (0.80; 1.11) | 4.95E-01 | 0.95 (0.70; 1.29) | 7.61E-01 | 0.96 (0.84; 1.11) | 6.15E-01 |
| C3-OH | 1.10 (0.94; 1.30) | 2.22E-01 | 1.01 (0.81; 1.26) | 9.28E-01 | 1.10 (0.86; 1.41) | 4.59E-01 |
| C3:1 | 1.03 (0.88; 1.21) | 7.19E-01 | 0.92 (0.70; 1.21) | 5.41E-01 | 1.09 (0.91; 1.31) | 3.32E-01 |
| C4 | 1.19 (1.04; 1.36) | 1.24E-02 | 1.03 (0.91; 1.17) | 5.90E-01 | 1.29 (0.90; 1.87) | 1.68E-01 |
| C4:1 | 1.05 (0.88; 1.25) | 5.90E-01 | 0.89 (0.65; 1.23) | 4.82E-01 | 1.21 (0.97; 1.51) | 8.47E-02 |
| C5 | 1.41 (1.16; 1.71)* | 7.22E-04 | 1.02 (0.75; 1.40) | 8.82E-01 | 1.48 (1.21; 1.82) | 2.39E-04 |
| C5-DC (C6-OH) | 0.95 (0.82; 1.11) | 5.39E-01 | 1.00 (0.75; 1.33) | 9.96E-01 | 0.91 (0.75; 1.10) | 3.07E-01 |
| C5-M-DC | 1.10 (0.93; 1.29) | 2.77E-01 | 0.99 (0.75; 1.30) | 9.20E-01 | 1.10 (0.89; 1.36) | 3.90E-01 |
| C5-OH (C3-DC-M) | 1.35 (1.15; 1.59)* | 2.85E-04 | 1.10 (0.88; 1.38) | 3.75E-01 | 1.33 (1.03; 1.71) | 3.12E-02 |
| C5:1 | 0.95 (0.79; 1.14) | 5.99E-01 | 0.83 (0.63; 1.10) | 1.87E-01 | 0.97 (0.76; 1.22) | 7.71E-01 |
| C5:1-DC | 1.07 (0.91; 1.26) | 4.01E-01 | 0.89 (0.74; 1.06) | 1.89E-01 | 1.26 (1.03; 1.55) | 2.81E-02 |
| C6 (C4:1-DC) | 1.15 (0.98; 1.34) | 9.54E-02 | 0.98 (0.82; 1.17) | 7.97E-01 | 1.36 (1.04; 1.79) | 2.72E-02 |
| C6:1 | 1.18 (0.99; 1.40) | 6.64E-02 | 1.04 (0.82; 1.31) | 7.63E-01 | 1.36 (1.12; 1.66) | 2.34E-03 |
| C7-DC | 0.82 (0.67; 0.99) | 4.13E-02 | 0.95 (0.73; 1.24) | 7.06E-01 | 0.71 (0.59; 0.84) | 2.02E-04 |
| C8 | 0.98 (0.86; 1.12) | 7.87E-01 | 0.92 (0.79; 1.07) | 2.69E-01 | 1.18 (0.72; 1.94) | 5.06E-01 |
| C8:1 | 1.21 (1.01; 1.44) | 3.88E-02 | 1.19 (0.88; 1.62) | 2.48E-01 | 1.18 (0.97; 1.42) | 9.02E-02 |
| C9 | 1.04 (0.89; 1.21) | 6.58E-01 | 1.01 (0.82; 1.25) | 9.08E-01 | 1.05 (0.87; 1.26) | 6.11E-01 |
| C10 | 0.96 (0.84; 1.09) | 5.13E-01 | 0.91 (0.80; 1.04) | 1.75E-01 | 1.07 (0.65; 1.76) | 7.79E-01 |
| C10:1 | 0.93 (0.79; 1.09) | 3.62E-01 | 0.86 (0.72; 1.04) | 1.26E-01 | 1.02 (0.76; 1.36) | 9.12E-01 |
| C10:2 | 1.06 (0.88; 1.27) | 5.54E-01 | 0.99 (0.75; 1.29) | 9.13E-01 | 1.13 (0.90; 1.43) | 2.84E-01 |
| C12 | 0.98 (0.83; 1.15) | 7.62E-01 | 0.89 (0.76; 1.04) | 1.47E-01 | 1.04 (0.69; 1.57) | 8.63E-01 |
| C12-DC | 0.78 (0.66; 0.92)* | 3.72E-03 | 0.99 (0.76; 1.29) | 9.47E-01 | 0.75 (0.62; 0.92) | 4.96E-03 |
| C12:1 | 0.83 (0.67; 1.03) | 8.44E-02 | 0.77 (0.62; 0.95) | 1.56E-02 | 0.91 (0.65; 1.27) | 5.79E-01 |
| C14 | 1.18 (0.99; 1.41) | 6.77E-02 | 0.94 (0.78; 1.15) | 5.57E-01 | 1.44 (1.09; 1.91) | 1.18E-02 |
| C14:1 | 0.90 (0.74; 1.10) | 3.12E-01 | 0.80 (0.65; 1.00) | 4.99E-02 | 0.95 (0.70; 1.30) | 7.66E-01 |
| C14:1-OH | 0.88 (0.73; 1.05) | 1.54E-01 | 0.85 (0.66; 1.08) | 1.82E-01 | 0.88 (0.71; 1.08) | 2.21E-01 |
| C14:2 | 0.85 (0.71; 1.01) | 5.83E-02 | 0.79 (0.66; 0.96) | 1.64E-02 | 0.80 (0.61; 1.05) | 1.04E-01 |
| C14:2-OH | 1.07 (0.90; 1.26) | 4.33E-01 | 0.89 (0.67; 1.19) | 4.41E-01 | 1.13 (0.96; 1.32) | 1.37E-01 |
| C16 | 1.38 (1.20; 1.59)* | 1.22E-05 | 1.05 (0.88; 1.25) | 5.78E-01 | 1.48 (1.24; 1.76) | 3.04E-05 |
| C16-OH | 1.09 (0.89; 1.34) | 3.90E-01 | 1.17 (0.87; 1.56) | 2.90E-01 | 0.97 (0.77; 1.23) | 8.24E-01 |
| C16:1 | 1.01 (0.82; 1.24) | 9.41E-01 | 0.86 (0.68; 1.07) | 1.75E-01 | 1.08 (0.80; 1.46) | 5.92E-01 |
| C16:1-OH | 1.15 (0.88; 1.49) | 2.96E-01 | 1.09 (0.86; 1.39) | 4.70E-01 | 1.12 (0.80; 1.57) | 5.07E-01 |
| C16:2 | 0.99 (0.81; 1.22) | 9.55E-01 | 0.84 (0.65; 1.09) | 1.77E-01 | 1.06 (0.79; 1.42) | 7.06E-01 |
| C16:2-OH | 1.10 (0.90; 1.34) | 3.69E-01 | 0.99 (0.74; 1.33) | 9.72E-01 | 1.10 (0.88; 1.38) | 4.01E-01 |
| C18 | 1.23 (1.05; 1.44)* | 9.43E-03 | 0.93 (0.77; 1.12) | 4.38E-01 | 1.34 (1.10; 1.64) | 4.95E-03 |
| C18:1 | 1.11 (0.90; 1.37) | 3.31E-01 | 0.89 (0.70; 1.14) | 3.61E-01 | 1.16 (0.87; 1.53) | 3.08E-01 |
| C18:1-OH | 0.92 (0.75; 1.12) | 3.95E-01 | 1.28 (0.84; 1.95) | 2.39E-01 | 0.95 (0.78; 1.15) | 5.79E-01 |
| C18:2 | 1.05 (0.87; 1.26) | 6.34E-01 | 0.78 (0.59; 1.03) | 7.78E-02 | 1.03 (0.79; 1.36) | 8.07E-01 |
| **Lysophosphatidylcholines** |  |  |  |  |  |  |
| Lyso PC a C6:0 | 1.02 (0.79; 1.32) | 8.68E-01 | 0.96 (0.75; 1.23) | 7.62E-01 | 1.15 (0.79; 1.67) | 4.55E-01 |
| Lyso PC a C14:0 | 1.32 (1.12; 1.55)* | 8.62E-04 | 1.33 (1.11; 1.58) | 2.44E-03 | 1.34 (1.09; 1.65) | 5.89E-03 |
| Lyso PC a C16:0 | 1.00 (0.83; 1.20) | 9.86E-01 | 0.89 (0.70; 1.12) | 3.03E-01 | 0.96 (0.75; 1.24) | 7.77E-01 |
| Lyso PC a C16:1 | 1.17 (0.97; 1.40) | 9.44E-02 | 1.20 (0.96; 1.49) | 1.01E-01 | 1.23 (1.01; 1.50) | 4.32E-02 |
| Lyso PC a C17:0 | 0.81 (0.67; 0.98) | 3.38E-02 | 0.75 (0.59; 0.95) | 1.88E-02 | 0.87 (0.69; 1.09) | 2.28E-01 |
| Lyso PC a C18:0 | 1.00 (0.83; 1.20) | 9.79E-01 | 0.79 (0.60; 1.05) | 1.05E-01 | 1.09 (0.91; 1.31) | 3.47E-01 |
| Lyso PC a C18:1 | 0.89 (0.72; 1.10) | 2.69E-01 | 0.69 (0.54; 0.89) | 4.37E-03 | 0.95 (0.73; 1.23) | 6.81E-01 |
| Lyso PC a C18:2 | 0.86 (0.72; 1.03) | 1.11E-01 | 0.70 (0.56; 0.86) | 1.28E-03 | 0.87 (0.67; 1.13) | 2.90E-01 |
| Lyso PC a C20:3 | 1.16 (0.95; 1.42) | 1.39E-01 | 0.98 (0.76; 1.27) | 9.01E-01 | 1.31 (1.02; 1.69) | 3.74E-02 |
| Lyso PC a C20:4 | 0.96 (0.78; 1.18) | 6.97E-01 | 0.73 (0.60; 0.88) | 1.59E-03 | 1.02 (0.74; 1.41) | 8.94E-01 |
| Lyso PC a C24:0 | 0.99 (0.86; 1.15) | 9.42E-01 | 0.97 (0.72; 1.29) | 8.10E-01 | 1.08 (0.93; 1.25) | 3.17E-01 |
| Lyso PC a C26:0 | 1.04 (0.90; 1.21) | 5.81E-01 | 1.24 (0.99; 1.55) | 5.57E-02 | 1.06 (0.91; 1.23) | 4.79E-01 |
| Lyso PC a C26:1 | 0.98 (0.84; 1.14) | 8.20E-01 | 1.03 (0.81; 1.32) | 8.01E-01 | 1.03 (0.88; 1.21) | 6.82E-01 |
| Lyso PC a C28:0 | 1.01 (0.88; 1.15) | 9.21E-01 | 1.21 (0.94; 1.55) | 1.34E-01 | 1.04 (0.89; 1.22) | 6.34E-01 |
| Lyso PC a C28:1 | 0.96 (0.82; 1.11) | 5.49E-01 | 1.13 (0.84; 1.52) | 4.01E-01 | 1.08 (0.91; 1.30) | 3.66E-01 |
| **Diacyl phosphatidylcholines** |  |  |  |  |  |  |
| PC aa C24:0 | 1.10 (0.94; 1.27) | 2.30E-01 | 1.21 (0.92; 1.60) | 1.61E-01 | 1.14 (0.98; 1.32) | 9.01E-02 |
| PC aa C26:0 | 1.05 (0.90; 1.22) | 5.46E-01 | 1.22 (0.97; 1.53) | 9.06E-02 | 1.07 (0.91; 1.26) | 3.91E-01 |
| PC aa C28:1 | 1.21 (1.01; 1.45) | 3.56E-02 | 1.23 (0.93; 1.65) | 1.48E-01 | 1.41 (1.17; 1.71) | 4.56E-04 |
| PC aa C30:0 | 1.24 (1.05; 1.46) | 1.19E-02 | 1.39 (1.09; 1.78) | 8.06E-03 | 1.36 (1.13; 1.63) | 1.43E-03 |
| PC aa C30:2 | 1.05 (0.87; 1.27) | 5.96E-01 | 1.01 (0.81; 1.26) | 9.27E-01 | 1.14 (0.87; 1.50) | 3.46E-01 |
| PC aa C32:0 | 1.14 (0.98; 1.32) | 8.92E-02 | 1.05 (0.83; 1.32) | 6.82E-01 | 1.34 (1.15; 1.57) | 2.91E-04 |
| PC aa C32:1 | 1.45 (1.25; 1.69)* | 3.02E-06 | 1.65 (1.38; 1.97) | 5.06E-07 | 1.54 (1.28; 1.85) | 1.18E-05 |
| PC aa C32:2 | 1.20 (1.00; 1.45) | 4.79E-02 | 1.53 (1.14; 2.06) | 5.45E-03 | 1.31 (1.05; 1.63) | 1.66E-02 |
| PC aa C32:3 | 1.11 (0.94; 1.31) | 2.09E-01 | 1.18 (0.89; 1.56) | 2.48E-01 | 1.46 (1.23; 1.74) | 4.08E-05 |
| PC aa C34:1 | 1.26 (1.08; 1.48)* | 4.05E-03 | 1.35 (1.10; 1.67) | 5.78E-03 | 1.36 (1.15; 1.61) | 4.76E-04 |
| PC aa C34:2 | 1.13 (0.97; 1.32) | 1.14E-01 | 1.21 (0.91; 1.62) | 1.90E-01 | 1.23 (1.02; 1.47) | 2.64E-02 |
| PC aa C34:3 | 1.16 (0.98; 1.37) | 7.51E-02 | 1.55 (1.19; 2.01) | 1.35E-03 | 1.39 (1.15; 1.69) | 1.07E-03 |
| PC aa C34:4 | 1.31 (1.14; 1.50)* | 2.42E-04 | 1.60 (1.24; 2.05) | 3.86E-04 | 1.49 (1.28; 1.72) | 1.00E-06 |
| PC aa C36:0 | 1.02 (0.87; 1.19) | 8.49E-01 | 0.87 (0.71; 1.07) | 1.72E-01 | 1.37 (1.07; 1.75) | 1.20E-02 |
| PC aa C36:1 | 1.30 (1.09; 1.55)* | 3.33E-03 | 1.52 (1.11; 2.08) | 1.06E-02 | 1.45 (1.22; 1.72) | 4.64E-05 |
| PC aa C36:2 | 1.14 (0.98; 1.33) | 8.19E-02 | 1.12 (0.81; 1.55) | 4.93E-01 | 1.34 (1.14; 1.58) | 6.52E-04 |
| PC aa C36:3 | 1.12 (0.96; 1.31) | 1.43E-01 | 1.37 (1.05; 1.79) | 2.10E-02 | 1.32 (1.11; 1.58) | 2.52E-03 |
| PC aa C36:4 | 1.09 (0.92; 1.29) | 2.98E-01 | 1.12 (0.87; 1.45) | 3.74E-01 | 1.25 (1.04; 1.52) | 2.01E-02 |
| PC aa C36:5 | 1.28 (1.08; 1.52)* | 4.81E-03 | 1.16 (0.92; 1.46) | 2.02E-01 | 1.46 (1.18; 1.81) | 5.81E-04 |
| PC aa C36:6 | 1.28 (1.08; 1.53)* | 4.64E-03 | 1.33 (1.07; 1.65) | 1.06E-02 | 1.46 (1.22; 1.76) | 8.72E-05 |
| PC aa C38:0 | 0.95 (0.80; 1.14) | 5.82E-01 | 0.82 (0.66; 1.03) | 9.22E-02 | 1.22 (0.96; 1.54) | 1.03E-01 |
| PC aa C38:1 | 1.08 (0.91; 1.29) | 3.81E-01 | 0.94 (0.73; 1.21) | 6.25E-01 | 1.16 (0.95; 1.42) | 1.43E-01 |
| PC aa C38:3 | 1.39 (1.22; 1.60)* | 2.92E-06 | 1.81 (1.47; 2.21) | 1.80E-07 | 1.64 (1.44; 1.87) | 1.05E-10 |
| PC aa C38:4 | 1.19 (1.02; 1.38) | 2.99E-02 | 1.15 (0.88; 1.49) | 2.96E-01 | 1.44 (1.22; 1.69) | 2.71E-05 |
| PC aa C38:5 | 1.25 (1.06; 1.48)* | 9.24E-03 | 1.17 (0.93; 1.47) | 1.82E-01 | 1.48 (1.24; 1.76) | 4.07E-05 |
| PC aa C38:6 | 1.14 (0.98; 1.34) | 8.86E-02 | 1.08 (0.85; 1.37) | 5.40E-01 | 1.29 (1.08; 1.54) | 4.62E-03 |
| PC aa C40:1 | 0.94 (0.79; 1.11) | 4.50E-01 | 0.83 (0.66; 1.04) | 1.05E-01 | 1.15 (0.95; 1.40) | 1.52E-01 |
| PC aa C40:2 | 1.03 (0.87; 1.22) | 7.03E-01 | 0.87 (0.69; 1.11) | 2.63E-01 | 1.24 (1.02; 1.52) | 2.98E-02 |
| PC aa C40:3 | 1.08 (0.91; 1.28) | 3.88E-01 | 0.97 (0.74; 1.26) | 8.09E-01 | 1.28 (1.05; 1.56) | 1.40E-02 |
| PC aa C40:4 | 1.21 (1.01; 1.46) | 3.94E-02 | 1.29 (0.99; 1.67) | 6.17E-02 | 1.32 (1.09; 1.61) | 4.96E-03 |
| PC aa C40:5 | 1.45 (1.22; 1.72)* | 4.15E-05 | 1.44 (1.17; 1.76) | 6.38E-04 | 1.55 (1.29; 1.86) | 9.18E-06 |
| PC aa C40:6 | 1.32 (1.13; 1.55)* | 4.71E-04 | 1.27 (1.05; 1.52) | 1.38E-02 | 1.50 (1.28; 1.76) | 2.47E-06 |
| PC aa C42:0 | 0.81 (0.69; 0.96) | 1.54E-02 | 0.68 (0.55; 0.85) | 6.97E-04 | 1.01 (0.82; 1.26) | 9.03E-01 |
| PC aa C42:1 | 0.85 (0.72; 1.00) | 5.67E-02 | 0.65 (0.52; 0.81) | 2.15E-04 | 1.06 (0.88; 1.27) | 5.24E-01 |
| PC aa C42:2 | 0.98 (0.83; 1.16) | 8.33E-01 | 0.77 (0.63; 0.93) | 8.19E-03 | 1.22 (1.02; 1.46) | 3.13E-02 |
| PC aa C42:4 | 0.89 (0.78; 1.03) | 1.11E-01 | 0.74 (0.58; 0.95) | 1.95E-02 | 1.05 (0.90; 1.23) | 5.32E-01 |
| PC aa C42:5 | 1.08 (0.91; 1.27) | 3.84E-01 | 1.10 (0.85; 1.42) | 4.77E-01 | 1.18 (0.96; 1.44) | 1.07E-01 |
| PC aa C42:6 | 0.97 (0.83; 1.15) | 7.56E-01 | 1.11 (0.83; 1.49) | 4.60E-01 | 1.10 (0.91; 1.34) | 3.05E-01 |
| **Acyl-alkyl phosphatidylcholines** |  |  |  |  |  |  |
| PC ae C30:0 | 0.99 (0.85; 1.16) | 8.94E-01 | 1.10 (0.88; 1.38) | 3.87E-01 | 1.15 (0.93; 1.42) | 1.86E-01 |
| PC ae C30:1 | 0.94 (0.81; 1.09) | 4.21E-01 | 1.03 (0.80; 1.33) | 8.23E-01 | 1.02 (0.85; 1.22) | 8.24E-01 |
| PC ae C30:2 | 1.12 (0.95; 1.32) | 1.79E-01 | 1.08 (0.87; 1.35) | 4.81E-01 | 1.40 (1.15; 1.71) | 1.14E-03 |
| PC ae C32:1 | 0.92 (0.77; 1.10) | 3.49E-01 | 0.86 (0.66; 1.12) | 2.71E-01 | 1.16 (0.93; 1.44) | 1.84E-01 |
| PC ae C32:2 | 0.87 (0.74; 1.02) | 7.94E-02 | 0.84 (0.68; 1.03) | 9.72E-02 | 1.18 (0.92; 1.52) | 1.81E-01 |
| PC ae C34:0 | 1.10 (0.93; 1.29) | 2.63E-01 | 1.10 (0.83; 1.45) | 5.04E-01 | 1.25 (1.08; 1.46) | 4.09E-03 |
| PC ae C34:1 | 0.93 (0.79; 1.10) | 3.85E-01 | 0.98 (0.78; 1.22) | 8.25E-01 | 1.15 (0.93; 1.43) | 1.90E-01 |
| PC ae C34:2 | 0.79 (0.66; 0.96) | 1.66E-02 | 0.76 (0.59; 0.98) | 3.50E-02 | 1.00 (0.77; 1.28) | 9.72E-01 |
| PC ae C34:3 | 0.82 (0.67; 1.00) | 5.28E-02 | 0.65 (0.47; 0.90) | 1.04E-02 | 1.06 (0.84; 1.33) | 6.33E-01 |
| PC ae C36:0 | 1.06 (0.88; 1.27) | 5.49E-01 | 0.95 (0.74; 1.23) | 6.89E-01 | 1.37 (1.11; 1.69) | 3.61E-03 |
| PC ae C36:1 | 1.05 (0.89; 1.23) | 5.85E-01 | 1.16 (0.86; 1.56) | 3.36E-01 | 1.23 (1.02; 1.47) | 2.63E-02 |
| PC ae C36:2 | 0.80 (0.67; 0.95) | 1.28E-02 | 0.79 (0.63; 0.98) | 2.96E-02 | 0.98 (0.78; 1.23) | 8.57E-01 |
| PC ae C36:3 | 0.86 (0.71; 1.05) | 1.34E-01 | 0.78 (0.59; 1.05) | 9.68E-02 | 1.12 (0.87; 1.45) | 3.64E-01 |
| PC ae C36:4 | 1.03 (0.84; 1.26) | 7.87E-01 | 0.85 (0.66; 1.11) | 2.34E-01 | 1.27 (1.01; 1.61) | 4.32E-02 |
| PC ae C36:5 | 1.07 (0.88; 1.29) | 4.94E-01 | 0.79 (0.61; 1.03) | 7.79E-02 | 1.35 (1.12; 1.61) | 1.50E-03 |
| PC ae C38:0 | 1.08 (0.92; 1.27) | 3.26E-01 | 1.08 (0.83; 1.41) | 5.48E-01 | 1.35 (1.10; 1.65) | 4.97E-03 |
| PC ae C38:1 | 0.91 (0.79; 1.04) | 1.74E-01 | 0.94 (0.79; 1.12) | 4.94E-01 | 0.98 (0.79; 1.20) | 8.26E-01 |
| PC ae C38:2 | 0.86 (0.74; 0.99) | 4.23E-02 | 0.84 (0.69; 1.01) | 7.02E-02 | 1.09 (0.90; 1.33) | 3.80E-01 |
| PC ae C38:3 | 0.96 (0.82; 1.13) | 6.38E-01 | 1.13 (0.83; 1.54) | 4.32E-01 | 1.21 (1.01; 1.44) | 3.47E-02 |
| PC ae C38:4 | 0.89 (0.75; 1.05) | 1.65E-01 | 0.82 (0.61; 1.09) | 1.64E-01 | 1.08 (0.91; 1.29) | 3.87E-01 |
| PC ae C38:5 | 0.96 (0.77; 1.19) | 7.06E-01 | 0.76 (0.60; 0.97) | 2.78E-02 | 1.19 (0.95; 1.49) | 1.33E-01 |
| PC ae C38:6 | 1.08 (0.89; 1.30) | 4.37E-01 | 0.89 (0.70; 1.13) | 3.42E-01 | 1.40 (1.13; 1.75) | 3.07E-03 |
| PC ae C40:0 | 0.88 (0.75; 1.04) | 1.36E-01 | 0.81 (0.61; 1.07) | 1.38E-01 | 1.11 (0.91; 1.35) | 3.14E-01 |
| PC ae C40:1 | 1.00 (0.84; 1.19) | 9.90E-01 | 0.92 (0.70; 1.22) | 5.67E-01 | 1.24 (1.00; 1.54) | 4.56E-02 |
| PC ae C40:2 | 0.97 (0.83; 1.14) | 7.44E-01 | 0.96 (0.77; 1.19) | 6.85E-01 | 1.15 (0.97; 1.38) | 1.12E-01 |
| PC ae C40:3 | 0.82 (0.71; 0.95)* | 6.47E-03 | 0.73 (0.56; 0.96) | 2.57E-02 | 1.10 (0.91; 1.32) | 3.24E-01 |
| PC ae C40:4 | 0.84 (0.73; 0.97) | 2.03E-02 | 0.73 (0.59; 0.91) | 4.79E-03 | 1.03 (0.88; 1.22) | 6.84E-01 |
| PC ae C40:5 | 0.89 (0.75; 1.05) | 1.58E-01 | 0.67 (0.53; 0.84) | 8.20E-04 | 1.09 (0.92; 1.30) | 2.98E-01 |
| PC ae C40:6 | 0.92 (0.78; 1.09) | 3.47E-01 | 0.82 (0.63; 1.07) | 1.40E-01 | 1.11 (0.93; 1.33) | 2.56E-01 |
| PC ae C42:0 | 0.79 (0.68; 0.92)* | 2.68E-03 | 0.81 (0.61; 1.07) | 1.36E-01 | 0.92 (0.77; 1.12) | 4.07E-01 |
| PC ae C42:1 | 1.00 (0.85; 1.18) | 9.69E-01 | 0.95 (0.74; 1.23) | 7.18E-01 | 1.25 (1.05; 1.48) | 1.21E-02 |
| PC ae C42:2 | 1.03 (0.85; 1.23) | 7.86E-01 | 0.88 (0.65; 1.18) | 3.85E-01 | 1.27 (1.04; 1.55) | 2.04E-02 |
| PC ae C42:3 | 0.84 (0.72; 0.97) | 1.62E-02 | 0.72 (0.55; 0.94) | 1.65E-02 | 1.06 (0.88; 1.29) | 5.21E-01 |
| PC ae C42:4 | 0.79 (0.68; 0.92)* | 2.07E-03 | 0.73 (0.61; 0.88) | 9.24E-04 | 0.95 (0.79; 1.14) | 5.78E-01 |
| PC ae C42:5 | 0.79 (0.67; 0.93)* | 5.71E-03 | 0.64 (0.52; 0.79) | 6.03E-05 | 1.00 (0.82; 1.23) | 9.79E-01 |
| PC ae C44:3 | 1.04 (0.90; 1.22) | 5.76E-01 | 0.92 (0.64; 1.31) | 6.32E-01 | 1.28 (1.09; 1.50) | 3.41E-03 |
| PC ae C44:4 | 0.88 (0.75; 1.03) | 1.20E-01 | 0.76 (0.63; 0.93) | 8.95E-03 | 1.05 (0.87; 1.27) | 5.77E-01 |
| PC ae C44:5 | 0.84 (0.70; 1.02) | 7.45E-02 | 0.69 (0.53; 0.90) | 6.05E-03 | 1.02 (0.82; 1.27) | 8.45E-01 |
| PC ae C44:6 | 0.78 (0.66; 0.92)* | 3.77E-03 | 0.66 (0.53; 0.82) | 3.79E-04 | 0.92 (0.74; 1.14) | 4.46E-01 |
| **Sphingomyelins** |  |  |  |  |  |  |
| SM (OH) C14:1 | 0.96 (0.80; 1.16) | 6.83E-01 | 0.78 (0.63; 0.98) | 3.32E-02 | 1.24 (0.99; 1.56) | 6.66E-02 |
| SM (OH) C16:1 | 0.92 (0.77; 1.09) | 3.20E-01 | 0.79 (0.62; 1.02) | 6.92E-02 | 1.14 (0.93; 1.39) | 1.95E-01 |
| SM (OH) C22:1 | 1.15 (0.97; 1.37) | 1.05E-01 | 1.03 (0.78; 1.36) | 8.51E-01 | 1.40 (1.19; 1.65) | 1.10E-04 |
| SM (OH) C22:2 | 0.95 (0.81; 1.12) | 5.55E-01 | 0.95 (0.72; 1.25) | 7.01E-01 | 1.24 (1.01; 1.52) | 4.25E-02 |
| SM (OH) C24:1 | 1.13 (0.96; 1.35) | 1.49E-01 | 0.92 (0.70; 1.21) | 5.58E-01 | 1.31 (1.12; 1.54) | 1.29E-03 |
| SM C16:0 | 0.95 (0.80; 1.14) | 5.86E-01 | 0.68 (0.54; 0.87) | 2.49E-03 | 1.28 (1.05; 1.55) | 1.49E-02 |
| SM C16:1 | 1.06 (0.90; 1.25) | 4.55E-01 | 0.96 (0.73; 1.28) | 7.91E-01 | 1.44 (1.20; 1.72) | 1.12E-04 |
| SM C18:0 | 1.09 (0.93; 1.27) | 2.70E-01 | 1.07 (0.83; 1.37) | 6.06E-01 | 1.28 (1.08; 1.53) | 6.20E-03 |
| SM C18:1 | 1.01 (0.87; 1.17) | 9.09E-01 | 1.04 (0.81; 1.34) | 7.43E-01 | 1.22 (1.03; 1.45) | 2.31E-02 |
| SM C20:2 | 0.75 (0.64; 0.89)* | 9.14E-04 | 0.94 (0.72; 1.23) | 6.43E-01 | 0.85 (0.67; 1.08) | 1.80E-01 |
| SM C22:3 | 0.76 (0.65; 0.89)* | 5.42E-04 | 0.81 (0.60; 1.10) | 1.75E-01 | 0.81 (0.68; 0.95) | 1.14E-02 |
| SM C24:0 | 1.25 (1.09; 1.44)* | 1.98E-03 | 0.98 (0.75; 1.27) | 8.49E-01 | 1.45 (1.23; 1.70) | 1.73E-05 |
| SM C24:1 | 1.04 (0.89; 1.23) | 6.20E-01 | 0.86 (0.68; 1.08) | 1.94E-01 | 1.28 (1.09; 1.51) | 3.98E-03 |
| SM C26:0 | 1.00 (0.85; 1.17) | 9.81E-01 | 0.93 (0.70; 1.24) | 6.27E-01 | 1.11 (0.96; 1.28) | 1.54E-01 |
| SM C26:1 | 1.04 (0.88; 1.23) | 6.23E-01 | 0.85 (0.65; 1.10) | 2.18E-01 | 1.22 (1.03; 1.43) | 2.14E-02 |
| **Amino acids** |  |  |  |  |  |  |
| Arginine | 0.93 (0.76; 1.14) | 5.02E-01 | 0.93 (0.75; 1.16) | 5.32E-01 | 0.97 (0.75; 1.26) | 8.09E-01 |
| Glutamine | 0.96 (0.78; 1.18) | 6.87E-01 | 1.06 (0.79; 1.43) | 7.00E-01 | 0.96 (0.76; 1.21) | 7.05E-01 |
| Glycine | 0.85 (0.74; 0.98) | 2.35E-02 | 0.91 (0.55; 1.53) | 7.31E-01 | 0.94 (0.81; 1.10) | 4.41E-01 |
| Histidine | 0.86 (0.71; 1.05) | 1.47E-01 | 1.41 (1.09; 1.81) | 8.35E-03 | 0.77 (0.64; 0.91) | 3.06E-03 |
| Methionine | 1.16 (0.93; 1.45) | 1.88E-01 | 1.24 (0.95; 1.60) | 1.09E-01 | 0.98 (0.74; 1.29) | 8.77E-01 |
| Ornithine | 1.18 (0.98; 1.44) | 8.49E-02 | 1.08 (0.87; 1.34) | 4.58E-01 | 1.19 (0.94; 1.51) | 1.48E-01 |
| Phenylalanine | 1.27 (1.06; 1.53)* | 9.89E-03 | 1.29 (1.03; 1.62) | 2.85E-02 | 1.13 (0.89; 1.43) | 3.05E-01 |
| Proline | 1.10 (0.92; 1.32) | 2.80E-01 | 0.96 (0.86; 1.07) | 4.38E-01 | 1.19 (0.86; 1.65) | 2.87E-01 |
| Serine | 0.75 (0.65; 0.87)* | 2.21E-04 | 0.74 (0.50; 1.10) | 1.33E-01 | 0.83 (0.70; 1.00) | 4.54E-02 |
| Threonine | 0.79 (0.65; 0.97) | 2.72E-02 | 0.82 (0.59; 1.15) | 2.43E-01 | 0.80 (0.65; 0.99) | 3.93E-02 |
| Tryptophan | 1.28 (1.09; 1.51)* | 3.04E-03 | 1.29 (1.03; 1.63) | 2.82E-02 | 1.18 (0.96; 1.45) | 1.14E-01 |
| Tyrosine | 1.49 (1.14; 1.94)* | 3.60E-03 | 1.78 (1.47; 2.16) | 6.96E-08 | 1.33 (0.96; 1.84) | 8.37E-02 |
| Valine | 1.34 (1.13; 1.58)* | 7.05E-04 | 1.13 (0.85; 1.51) | 3.87E-01 | 1.19 (0.92; 1.53) | 1.84E-01 |
| (Iso)Leucine | 1.47 (1.27; 1.71)* | 6.08E-07 | 1.21 (0.97; 1.52) | 9.06E-02 | 1.45 (1.05; 1.98) | 2.27E-02 |
| **Hexoses** |  |  |  |  |  |  |
| Hexoses | 1.30 (1.11; 1.53)* | 1.33E-03 | 1.10 (0.81; 1.50) | 5.35E-01 | 1.27 (1.03; 1.57) | 2.86E-02 |
| **Aggregate measures** |  |  |  |  |  |  |
| (C2 + C3) / C0 | 0.72 (0.56; 0.92)* | 8.27E-03 | 1.00 (0.66; 1.50) | 9.92E-01 | 0.70 (0.56; 0.86) | 1.39E-03 |
| Aromatic amino acids (AAA) | 1.45 (1.17; 1.79)* | 6.79E-04 | 1.60 (1.27; 2.01) | 1.29E-04 | 1.29 (0.98; 1.69) | 6.96E-02 |
| C2 / C0 | 0.71 (0.56; 0.91)* | 7.00E-03 | 1.00 (0.66; 1.50) | 9.87E-01 | 0.69 (0.56; 0.86) | 1.34E-03 |
| (C16 + C18) / C0 | 0.91 (0.75; 1.11) | 3.52E-01 | 0.96 (0.74; 1.25) | 7.66E-01 | 0.89 (0.70; 1.14) | 3.59E-01 |
| MUFA(PC) | 1.29 (1.09; 1.52)* | 2.65E-03 | 1.44 (1.14; 1.81) | 2.95E-03 | 1.41 (1.19; 1.66) | 9.39E-05 |
| MUFA(PC) / SFA(PC) | 1.27 (1.04; 1.55) | 1.98E-02 | 1.40 (1.15; 1.70) | 1.13E-03 | 1.18 (0.90; 1.55) | 2.19E-01 |
| Ornithine / Arginine | 1.21 (1.02; 1.45) | 3.24E-02 | 1.09 (0.92; 1.29) | 2.92E-01 | 1.22 (0.93; 1.61) | 1.55E-01 |
| PUFA(PC) | 1.20 (1.03; 1.39) | 1.71E-02 | 1.29 (0.96; 1.75) | 9.30E-02 | 1.41 (1.21; 1.65) | 2.85E-05 |
| PUFA(PC) / MUFA(PC) | 0.83 (0.69; 0.99) | 4.04E-02 | 0.79 (0.65; 0.97) | 2.21E-02 | 0.85 (0.68; 1.07) | 1.58E-01 |
| PUFA(PC) / SFA(PC) | 1.09 (0.94; 1.28) | 2.51E-01 | 1.09 (0.89; 1.33) | 4.03E-01 | 1.08 (0.83; 1.40) | 5.64E-01 |
| SFA(PC) | 1.12 (0.96; 1.32) | 1.60E-01 | 1.04 (0.82; 1.34) | 7.23E-01 | 1.44 (1.21; 1.72) | 1.03E-04 |
| Total PC + Total SM | 1.20 (1.03; 1.39) | 2.08E-02 | 1.25 (0.93; 1.68) | 1.35E-01 | 1.42 (1.22; 1.66) | 1.34E-05 |
| Total AC / C0 | 0.69 (0.54; 0.88)* | 3.40E-03 | 0.95 (0.61; 1.46) | 8.01E-01 | 0.68 (0.54; 0.85) | 8.54E-04 |
| Total AC-DC / Total AC | 0.91 (0.75; 1.09) | 2.97E-01 | 1.08 (0.84; 1.39) | 5.59E-01 | 0.85 (0.71; 1.02) | 8.54E-02 |
| Total AC-OH / Total AC | 0.97 (0.82; 1.15) | 7.61E-01 | 1.08 (0.82; 1.41) | 5.90E-01 | 0.95 (0.81; 1.11) | 5.12E-01 |
| Total lysoPC | 0.95 (0.78; 1.16) | 6.11E-01 | 0.77 (0.60; 1.00) | 4.58E-02 | 0.98 (0.76; 1.26) | 8.62E-01 |
| Total lysoPC / Total PC | 0.84 (0.70; 1.01) | 5.82E-02 | 0.62 (0.47; 0.84) | 1.97E-03 | 0.73 (0.59; 0.90) | 3.70E-03 |
| Total PC | 1.22 (1.05; 1.42)* | 8.66E-03 | 1.35 (1.01; 1.82) | 4.55E-02 | 1.42 (1.22; 1.66) | 1.38E-05 |
| Total diacyl PC | 1.25 (1.08; 1.45)* | 2.68E-03 | 1.41 (1.06; 1.88) | 1.82E-02 | 1.44 (1.24; 1.67) | 5.85E-06 |
| Total acyl-alkyl PC | 0.90 (0.75; 1.08) | 2.49E-01 | 0.73 (0.59; 0.91) | 5.43E-03 | 1.21 (0.98; 1.49) | 6.88E-02 |
| Total SM | 1.04 (0.87; 1.23) | 6.79E-01 | 0.81 (0.65; 1.02) | 6.71E-02 | 1.34 (1.13; 1.58) | 1.11E-03 |
| Total SM / (Total SM + Total PC) | 0.81 (0.68; 0.97) | 2.52E-02 | 0.66 (0.53; 0.83) | 4.19E-04 | 0.93 (0.74; 1.16) | 5.11E-01 |
| Total SM / Total PC | 0.82 (0.68; 0.98) | 2.76E-02 | 0.66 (0.53; 0.83) | 5.42E-04 | 0.93 (0.74; 1.16) | 5.18E-01 |
| Total SM-non OH | 1.04 (0.87; 1.23) | 6.76E-01 | 0.80 (0.64; 1.02) | 6.65E-02 | 1.33 (1.13; 1.58) | 1.07E-03 |
| Total SM-OH | 1.03 (0.86; 1.23) | 7.33E-01 | 0.91 (0.70; 1.17) | 4.46E-01 | 1.32 (1.10; 1.58) | 3.79E-03 |
| Total SM-OH / Total SM-non OH | 0.99 (0.81; 1.21) | 9.22E-01 | 1.11 (0.82; 1.51) | 4.79E-01 | 1.13 (0.89; 1.43) | 3.29E-01 |
| Tyrosine / Phenylalanine | 1.38 (1.09; 1.75)* | 7.44E-03 | 1.79 (1.49; 2.15) | 1.77E-08 | 1.31 (1.02; 1.70) | 3.73E-02 |

**Model 2 (adjusted for age, sex and total body fat)**

|  | **Visceral adipose tissue** | | | | | |
| --- | --- | --- | --- | --- | --- | --- |
|  | Total |  | Men |  | Women |  |
|  | (N = 174) |  | (N = 84) |  | (N = 90) |  |
|  | Estimate (95% CI) | P-value | Estimate (95% CI) | P-value | Estimate (95% CI) | P-value |
| **Acylcarnitines** |  |  |  |  |  |  |
| C0 | -2.7 (-8.7; 3.3) | 3.80E-01 | -3.4 (-13.4; 6.7) | 5.06E-01 | -1.0 (-7.8; 5.8) | 7.77E-01 |
| C2 | -3.9 (-10.0; 2.2) | 2.11E-01 | -6.5 (-23.3; 10.3) | 4.44E-01 | -3.4 (-8.1; 1.3) | 1.49E-01 |
| C3 | -3.2 (-8.8; 2.4) | 2.59E-01 | -4.4 (-11.1; 2.3) | 1.92E-01 | 1.3 (-6.3; 8.9) | 7.38E-01 |
| C3-DC (C4-OH) | 0.5 (-5.6; 6.7) | 8.62E-01 | 3.7 (-8.9; 16.3) | 5.66E-01 | -1.2 (-5.8; 3.5) | 6.19E-01 |
| C3-OH | 2.4 (-2.7; 7.5) | 3.49E-01 | 5.2 (-1.0; 11.3) | 9.74E-02 | -2.4 (-7.8; 2.9) | 3.69E-01 |
| C3:1 | -2.5 (-8.4; 3.3) | 3.94E-01 | -1.6 (-16.8; 13.6) | 8.37E-01 | -0.4 (-5.6; 4.9) | 8.85E-01 |
| C4 | -4.1 (-8.4; 0.2) | 6.28E-02 | -4.2 (-9.9; 1.5) | 1.47E-01 | -0.2 (-8.3; 7.9) | 9.63E-01 |
| C4:1 | -3.3 (-9.6; 2.9) | 2.98E-01 | -5.2 (-12.8; 2.5) | 1.81E-01 | 0.8 (-5.8; 7.3) | 8.17E-01 |
| C5 | 1.2 (-6.5; 8.8) | 7.64E-01 | -3.9 (-13.2; 5.4) | 4.04E-01 | 10.3 (1.7; 19.0) | 2.02E-02 |
| C5-DC (C6-OH) | -2.0 (-10.1; 6.0) | 6.22E-01 | -5.9 (-16.0; 4.3) | 2.53E-01 | 0.6 (-6.5; 7.6) | 8.75E-01 |
| C5-M-DC | -7.8 (-15.6; 0.0) | 4.95E-02 | -8.4 (-23.0; 6.1) | 2.52E-01 | -4.2 (-10.0; 1.5) | 1.47E-01 |
| C5-OH (C3-DC-M) | -0.9 (-7.4; 5.6) | 7.85E-01 | 1.9 (-9.0; 12.9) | 7.29E-01 | -2.4 (-7.7; 2.9) | 3.70E-01 |
| C5:1 | -4.0 (-9.5; 1.4) | 1.48E-01 | -5.3 (-13.8; 3.2) | 2.19E-01 | -0.4 (-6.3; 5.5) | 8.89E-01 |
| C5:1-DC | -2.7 (-8.8; 3.4) | 3.79E-01 | -6.9 (-17.2; 3.4) | 1.89E-01 | 0.3 (-4.9; 5.5) | 9.09E-01 |
| C6 (C4:1-DC) | -4.3 (-10.5; 1.8) | 1.68E-01 | -8.5 (-18.5; 1.5) | 9.51E-02 | -1.1 (-6.3; 4.2) | 6.91E-01 |
| C6:1 | -3.2 (-9.3; 2.8) | 2.95E-01 | -3.1 (-12.1; 6.0) | 5.05E-01 | 0.3 (-6.4; 6.9) | 9.40E-01 |
| C7-DC | -2.9 (-9.0; 3.2) | 3.46E-01 | -7.9 (-16.8; 1.0) | 8.14E-02 | -0.2 (-6.6; 6.2) | 9.55E-01 |
| C8 | -6.7 (-13.9; 0.4) | 6.58E-02 | -9.9 (-17.7; -2.1) | 1.36E-02 | -0.6 (-9.4; 8.3) | 8.97E-01 |
| C8:1 | 8.0 (0.0; 16.0) | 5.13E-02 | 7.8 (-10.3; 25.9) | 3.95E-01 | 6.7 (0.6; 12.8) | 3.23E-02 |
| C9 | -1.9 (-9.0; 5.2) | 6.01E-01 | -13.6 (-26.3; -1.0) | 3.42E-02 | 5.4 (-1.0; 11.7) | 9.67E-02 |
| C10 | -6.4 (-12.8; 0.0) | 5.06E-02 | -10.1 (-16.4; -3.8) | 2.10E-03 | -0.8 (-8.9; 7.4) | 8.48E-01 |
| C10:1 | -7.7 (-14.0; -1.4) | 1.75E-02 | -12.7 (-19.8; -5.5) | 6.83E-04 | 0.3 (-6.5; 7.0) | 9.35E-01 |
| C10:2 | -0.1 (-8.5; 8.4) | 9.89E-01 | -6.3 (-18.9; 6.3) | 3.21E-01 | 4.1 (-1.8; 10.1) | 1.73E-01 |
| C12 | -6.2 (-12.7; 0.2) | 5.88E-02 | -13.2 (-21.6; -4.9) | 2.21E-03 | -0.2 (-4.8; 4.5) | 9.45E-01 |
| C12-DC | -1.6 (-7.4; 4.1) | 5.78E-01 | -6.8 (-18.7; 5.0) | 2.55E-01 | 1.1 (-3.5; 5.8) | 6.32E-01 |
| C12:1 | -5.5 (-11.8; 0.8) | 8.77E-02 | -15.3 (-26.2; -4.4) | 6.66E-03 | -1.9 (-5.5; 1.6) | 2.87E-01 |
| C14 | -4.6 (-11.9; 2.6) | 2.11E-01 | -13.1 (-25.1; -1.1) | 3.27E-02 | 3.8 (-2.7; 10.3) | 2.54E-01 |
| C14:1 | -4.1 (-9.5; 1.2) | 1.28E-01 | -10.1 (-21.5; 1.3) | 8.28E-02 | -0.7 (-4.5; 3.0) | 6.94E-01 |
| C14:1-OH | -5.6 (-12.3; 1.2) | 1.05E-01 | -14.7 (-28.6; -0.9) | 3.75E-02 | -1.5 (-6.5; 3.6) | 5.62E-01 |
| C14:2 | -8.2 (-14.0; -2.4) | 5.53E-03 | -13.6 (-24.9; -2.2) | 1.98E-02 | -2.9 (-7.2; 1.4) | 1.78E-01 |
| C14:2-OH | -4.7 (-10.8; 1.5) | 1.40E-01 | -8.2 (-19.2; 2.8) | 1.41E-01 | 1.2 (-3.6; 5.9) | 6.21E-01 |
| C16 | -1.5 (-8.8; 5.8) | 6.87E-01 | -4.8 (-16.4; 6.8) | 4.16E-01 | 1.2 (-5.5; 7.9) | 7.26E-01 |
| C16-OH | 4.8 (0.0; 9.6) | 5.08E-02 | 5.6 (-1.6; 12.8) | 1.26E-01 | 3.4 (-1.8; 8.7) | 1.97E-01 |
| C16:1 | -2.9 (-8.9; 3.1) | 3.48E-01 | -5.9 (-16.9; 5.0) | 2.85E-01 | -1.5 (-6.2; 3.1) | 5.12E-01 |
| C16:1-OH | 2.6 (-4.3; 9.4) | 4.63E-01 | -1.0 (-13.7; 11.6) | 8.74E-01 | 4.4 (-1.5; 10.2) | 1.40E-01 |
| C16:2 | -5.5 (-11.9; 0.9) | 9.28E-02 | -11.0 (-22.6; 0.6) | 6.30E-02 | -0.8 (-6.1; 4.6) | 7.77E-01 |
| C16:2-OH | -0.6 (-7.2; 6.1) | 8.65E-01 | -12.2 (-23.8; -0.7) | 3.75E-02 | 4.2 (-1.1; 9.5) | 1.22E-01 |
| C18 | -2.7 (-9.8; 4.5) | 4.59E-01 | -3.8 (-15.3; 7.8) | 5.17E-01 | 0.3 (-6.6; 7.2) | 9.35E-01 |
| C18:1 | 0.8 (-4.5; 6.2) | 7.63E-01 | 2.3 (-5.6; 10.2) | 5.65E-01 | -0.4 (-5.4; 4.6) | 8.70E-01 |
| C18:1-OH | 4.7 (-0.1; 9.5) | 5.32E-02 | 6.3 (-11.5; 24.0) | 4.85E-01 | 1.9 (-3.1; 6.8) | 4.58E-01 |
| C18:2 | -5.5 (-11.6; 0.6) | 7.47E-02 | -8.6 (-18.4; 1.1) | 8.05E-02 | -1.3 (-8.7; 6.0) | 7.17E-01 |
| **Lysophosphatidylcholines** |  |  |  |  |  |  |
| Lyso PC a C6:0 | 1.9 (-4.5; 8.4) | 5.50E-01 | 2.9 (-12.2; 18.0) | 7.01E-01 | -0.5 (-4.9; 3.9) | 8.21E-01 |
| Lyso PC a C14:0 | 2.2 (-4.8; 9.3) | 5.33E-01 | 1.6 (-8.7; 12.0) | 7.56E-01 | 2.6 (-4.2; 9.4) | 4.53E-01 |
| Lyso PC a C16:0 | -6.9 (-14.9; 1.0) | 8.80E-02 | -11.2 (-25.4; 3.0) | 1.22E-01 | -4.2 (-10.3; 1.9) | 1.76E-01 |
| Lyso PC a C16:1 | -1.8 (-7.9; 4.3) | 5.65E-01 | -1.5 (-11.7; 8.7) | 7.71E-01 | -3.1 (-8.2; 2.0) | 2.35E-01 |
| Lyso PC a C17:0 | -9.1 (-16.8; -1.4) | 2.01E-02 | -12.0 (-25.9; 1.9) | 8.85E-02 | -5.9 (-11.1; -0.6) | 2.99E-02 |
| Lyso PC a C18:0 | -7.4 (-14.5; -0.2) | 4.33E-02 | -15.6 (-32.5; 1.3) | 6.98E-02 | -3.0 (-7.7; 1.7) | 2.07E-01 |
| Lyso PC a C18:1 | -6.8 (-13.8; 0.3) | 5.87E-02 | -11.5 (-25.7; 2.7) | 1.12E-01 | -3.7 (-9.3; 1.8) | 1.86E-01 |
| Lyso PC a C18:2 | -12.4 (-20.7; -4.2) | 3.41E-03 | -19.9 (-33.7; -6.2) | 5.06E-03 | -3.2 (-10.5; 4.1) | 3.93E-01 |
| Lyso PC a C20:3 | -4.9 (-12.0; 2.2) | 1.75E-01 | -8.3 (-19.1; 2.5) | 1.32E-01 | -1.5 (-7.9; 4.9) | 6.37E-01 |
| Lyso PC a C20:4 | -2.4 (-8.3; 3.4) | 4.14E-01 | -2.3 (-12.1; 7.5) | 6.41E-01 | 0.0 (-6.3; 6.3) | 1.00E+00 |
| Lyso PC a C24:0 | 1.7 (-4.5; 7.9) | 5.90E-01 | -1.9 (-13.9; 10.2) | 7.56E-01 | 3.8 (-0.9; 8.4) | 1.12E-01 |
| Lyso PC a C26:0 | 0.5 (-4.9; 5.9) | 8.50E-01 | -2.7 (-13.2; 7.8) | 6.12E-01 | 1.9 (-3.6; 7.5) | 4.96E-01 |
| Lyso PC a C26:1 | 0.6 (-5.0; 6.1) | 8.43E-01 | -1.8 (-13.7; 10.1) | 7.62E-01 | 2.0 (-3.0; 7.1) | 4.27E-01 |
| Lyso PC a C28:0 | 0.7 (-4.7; 6.2) | 7.95E-01 | -4.8 (-15.7; 6.2) | 3.90E-01 | 3.5 (-1.8; 8.8) | 1.90E-01 |
| Lyso PC a C28:1 | -0.5 (-5.9; 5.0) | 8.65E-01 | -3.0 (-15.0; 9.0) | 6.23E-01 | 1.1 (-4.3; 6.4) | 6.96E-01 |
| **Diacyl phosphatidylcholines** |  |  |  |  |  |  |
| PC aa C24:0 | 2.8 (-2.5; 8.0) | 2.96E-01 | -0.8 (-12.5; 10.9) | 8.91E-01 | 4.1 (-0.6; 8.9) | 8.66E-02 |
| PC aa C26:0 | 1.7 (-3.5; 6.9) | 5.12E-01 | 0.4 (-8.3; 9.0) | 9.32E-01 | 1.6 (-3.9; 7.0) | 5.72E-01 |
| PC aa C28:1 | 2.6 (-3.8; 8.9) | 4.29E-01 | 6.5 (-10.1; 23.2) | 4.36E-01 | -0.3 (-6.1; 5.5) | 9.18E-01 |
| PC aa C30:0 | 1.9 (-5.4; 9.2) | 6.07E-01 | -0.1 (-11.7; 11.5) | 9.89E-01 | 1.1 (-5.6; 7.8) | 7.49E-01 |
| PC aa C30:2 | -5.6 (-11.7; 0.4) | 6.88E-02 | -6.7 (-17.8; 4.3) | 2.29E-01 | -4.2 (-9.5; 1.0) | 1.10E-01 |
| PC aa C32:0 | 1.5 (-4.9; 7.9) | 6.47E-01 | 1.0 (-7.3; 9.2) | 8.16E-01 | -0.9 (-7.9; 6.1) | 8.09E-01 |
| PC aa C32:1 | 4.2 (-2.0; 10.5) | 1.82E-01 | 5.4 (-5.1; 16.0) | 3.10E-01 | 0.1 (-4.7; 4.8) | 9.76E-01 |
| PC aa C32:2 | 3.0 (-2.4; 8.5) | 2.73E-01 | 1.5 (-8.9; 11.8) | 7.81E-01 | 2.6 (-2.4; 7.5) | 3.05E-01 |
| PC aa C32:3 | -0.6 (-8.4; 7.1) | 8.72E-01 | -2.3 (-21.9; 17.2) | 8.12E-01 | 0.5 (-4.6; 5.7) | 8.42E-01 |
| PC aa C34:1 | 4.8 (-1.3; 10.8) | 1.25E-01 | 7.5 (-3.3; 18.2) | 1.72E-01 | 0.3 (-4.8; 5.3) | 9.12E-01 |
| PC aa C34:2 | 2.5 (-3.4; 8.3) | 4.08E-01 | 0.6 (-11.0; 12.2) | 9.18E-01 | 1.2 (-4.4; 6.7) | 6.72E-01 |
| PC aa C34:3 | 6.2 (0.0; 12.5) | 4.99E-02 | 11.4 (-2.9; 25.6) | 1.17E-01 | 1.4 (-3.6; 6.4) | 5.86E-01 |
| PC aa C34:4 | 8.4 (2.8; 14.0) | 3.47E-03 | 13.2 (0.7; 25.7) | 3.83E-02 | 5.0 (0.6; 9.5) | 2.79E-02 |
| PC aa C36:0 | -1.7 (-10.3; 6.9) | 6.97E-01 | -7.1 (-18.7; 4.6) | 2.32E-01 | 5.0 (-1.0; 11.0) | 1.04E-01 |
| PC aa C36:1 | 2.8 (-2.6; 8.2) | 3.06E-01 | 5.8 (-5.7; 17.2) | 3.18E-01 | -0.3 (-5.5; 5.0) | 9.20E-01 |
| PC aa C36:2 | 0.5 (-5.8; 6.8) | 8.82E-01 | -1.2 (-13.6; 11.2) | 8.48E-01 | 0.4 (-5.8; 6.5) | 9.04E-01 |
| PC aa C36:3 | 2.8 (-2.9; 8.5) | 3.35E-01 | 4.0 (-5.8; 13.8) | 4.21E-01 | -0.4 (-5.6; 4.7) | 8.72E-01 |
| PC aa C36:4 | 7.3 (1.7; 12.9) | 1.06E-02 | 12.9 (1.7; 24.1) | 2.44E-02 | 2.9 (-2.4; 8.2) | 2.78E-01 |
| PC aa C36:5 | 6.9 (0.9; 12.8) | 2.47E-02 | 3.5 (-5.6; 12.6) | 4.44E-01 | 7.4 (2.1; 12.7) | 6.79E-03 |
| PC aa C36:6 | 5.4 (-0.4; 11.2) | 6.91E-02 | 1.8 (-9.3; 12.9) | 7.46E-01 | 6.0 (1.2; 10.8) | 1.54E-02 |
| PC aa C38:0 | -3.3 (-11.3; 4.7) | 4.18E-01 | -9.5 (-21.0; 2.0) | 1.03E-01 | 3.0 (-3.1; 9.2) | 3.26E-01 |
| PC aa C38:1 | 0.2 (-6.5; 7.0) | 9.47E-01 | 0.7 (-10.2; 11.7) | 8.97E-01 | 1.2 (-4.4; 6.8) | 6.78E-01 |
| PC aa C38:3 | 3.8 (-2.6; 10.3) | 2.42E-01 | 7.2 (-4.8; 19.3) | 2.36E-01 | 0.4 (-5.5; 6.3) | 8.93E-01 |
| PC aa C38:4 | 7.6 (1.1; 14.0) | 2.19E-02 | 14.5 (-0.3; 29.3) | 5.55E-02 | 3.5 (-2.1; 9.2) | 2.21E-01 |
| PC aa C38:5 | 7.8 (2.6; 13.0) | 3.76E-03 | 9.6 (0.1; 19.0) | 4.79E-02 | 4.8 (-0.7; 10.3) | 8.49E-02 |
| PC aa C38:6 | 2.9 (-3.7; 9.6) | 3.87E-01 | -2.0 (-13.1; 9.2) | 7.27E-01 | 5.8 (0.6; 11.0) | 2.89E-02 |
| PC aa C40:1 | -6.4 (-14.4; 1.5) | 1.10E-01 | -13.1 (-24.1; -2.1) | 2.00E-02 | 1.6 (-5.3; 8.4) | 6.51E-01 |
| PC aa C40:2 | -2.9 (-10.4; 4.6) | 4.53E-01 | -10.2 (-22.4; 1.9) | 9.82E-02 | 2.7 (-3.5; 9.0) | 3.88E-01 |
| PC aa C40:3 | -3.2 (-10.8; 4.5) | 4.11E-01 | -11.0 (-22.4; 0.4) | 5.80E-02 | 1.1 (-5.9; 8.2) | 7.51E-01 |
| PC aa C40:4 | 3.6 (-2.0; 9.1) | 2.08E-01 | 7.8 (-5.5; 21.0) | 2.48E-01 | -0.4 (-5.0; 4.2) | 8.56E-01 |
| PC aa C40:5 | 6.2 (0.5; 11.8) | 3.21E-02 | 8.1 (-4.1; 20.4) | 1.89E-01 | 3.4 (-1.8; 8.7) | 1.96E-01 |
| PC aa C40:6 | 3.5 (-3.2; 10.1) | 3.07E-01 | -1.1 (-11.6; 9.5) | 8.40E-01 | 6.5 (0.8; 12.3) | 2.52E-02 |
| PC aa C42:0 | -8.1 (-15.1; -1.1) | 2.36E-02 | -12.8 (-24.6; -1.0) | 3.43E-02 | -3.4 (-9.6; 2.8) | 2.77E-01 |
| PC aa C42:1 | -6.3 (-12.3; -0.3) | 3.95E-02 | -8.3 (-21.4; 4.9) | 2.13E-01 | -4.7 (-10.6; 1.1) | 1.09E-01 |
| PC aa C42:2 | -4.3 (-12.0; 3.4) | 2.68E-01 | -6.4 (-17.5; 4.7) | 2.52E-01 | -0.3 (-7.5; 7.0) | 9.39E-01 |
| PC aa C42:4 | -1.6 (-7.9; 4.7) | 6.13E-01 | -4.0 (-21.1; 13.1) | 6.43E-01 | -2.1 (-6.9; 2.8) | 4.04E-01 |
| PC aa C42:5 | 3.9 (-3.2; 10.9) | 2.85E-01 | 0.3 (-11.4; 12.1) | 9.54E-01 | 4.0 (-2.1; 10.1) | 1.98E-01 |
| PC aa C42:6 | 0.6 (-5.4; 6.5) | 8.55E-01 | -2.5 (-13.6; 8.7) | 6.60E-01 | -1.5 (-7.6; 4.6) | 6.24E-01 |
| **Acyl-alkyl phosphatidylcholines** |  |  |  |  |  |  |
| PC ae C30:0 | -3.7 (-11.3; 3.9) | 3.41E-01 | -12.4 (-26.6; 1.8) | 8.58E-02 | 1.0 (-5.7; 7.7) | 7.67E-01 |
| PC ae C30:1 | -3.2 (-10.7; 4.2) | 3.97E-01 | -11.0 (-26.7; 4.7) | 1.66E-01 | 1.3 (-4.4; 6.9) | 6.58E-01 |
| PC ae C30:2 | 1.2 (-5.6; 8.1) | 7.19E-01 | -0.8 (-14.3; 12.8) | 9.12E-01 | 3.3 (-3.7; 10.3) | 3.57E-01 |
| PC ae C32:1 | -5.3 (-11.7; 1.1) | 1.06E-01 | -13.9 (-27.0; -0.9) | 3.71E-02 | -1.1 (-7.0; 4.7) | 6.98E-01 |
| PC ae C32:2 | -5.9 (-12.4; 0.7) | 7.85E-02 | -13.4 (-28.5; 1.7) | 8.08E-02 | -2.5 (-8.3; 3.2) | 3.88E-01 |
| PC ae C34:0 | -3.1 (-10.1; 3.9) | 3.89E-01 | -5.3 (-17.8; 7.3) | 4.04E-01 | -2.6 (-8.7; 3.5) | 3.99E-01 |
| PC ae C34:1 | -3.7 (-9.8; 2.5) | 2.45E-01 | -7.3 (-20.3; 5.8) | 2.70E-01 | -3.2 (-8.8; 2.5) | 2.67E-01 |
| PC ae C34:2 | -7.4 (-16.1; 1.3) | 9.39E-02 | -18.1 (-35.6; -0.6) | 4.28E-02 | -1.9 (-9.9; 6.0) | 6.28E-01 |
| PC ae C34:3 | -5.8 (-13.7; 2.2) | 1.53E-01 | -16.7 (-39.5; 6.0) | 1.47E-01 | -2.5 (-8.3; 3.3) | 3.98E-01 |
| PC ae C36:0 | 0.6 (-7.8; 9.1) | 8.84E-01 | -0.2 (-13.1; 12.6) | 9.71E-01 | -0.1 (-7.4; 7.2) | 9.75E-01 |
| PC ae C36:1 | 2.0 (-3.8; 7.9) | 4.92E-01 | 5.4 (-9.9; 20.6) | 4.83E-01 | -1.5 (-6.5; 3.6) | 5.64E-01 |
| PC ae C36:2 | -6.5 (-15.0; 2.0) | 1.34E-01 | -7.3 (-22.8; 8.1) | 3.49E-01 | -5.1 (-11.6; 1.5) | 1.28E-01 |
| PC ae C36:3 | -6.8 (-14.7; 1.0) | 8.82E-02 | -16.5 (-33.2; 0.1) | 5.16E-02 | -2.5 (-9.6; 4.7) | 4.97E-01 |
| PC ae C36:4 | 0.2 (-5.8; 6.1) | 9.52E-01 | -3.0 (-14.6; 8.6) | 6.11E-01 | 1.6 (-3.7; 6.8) | 5.59E-01 |
| PC ae C36:5 | 1.6 (-4.9; 8.1) | 6.25E-01 | -0.9 (-13.9; 12.1) | 8.92E-01 | 2.3 (-2.1; 6.7) | 2.94E-01 |
| PC ae C38:0 | 2.9 (-3.5; 9.2) | 3.74E-01 | -3.1 (-16.1; 9.9) | 6.34E-01 | 4.6 (-0.2; 9.5) | 6.23E-02 |
| PC ae C38:1 | -2.3 (-9.4; 4.7) | 5.17E-01 | -7.3 (-19.6; 5.0) | 2.43E-01 | 1.0 (-5.8; 7.7) | 7.81E-01 |
| PC ae C38:2 | -8.6 (-15.7; -1.6) | 1.69E-02 | -13.2 (-26.6; 0.3) | 5.48E-02 | -5.8 (-12.5; 0.9) | 9.07E-02 |
| PC ae C38:3 | -2.1 (-8.6; 4.4) | 5.31E-01 | -2.7 (-14.4; 9.0) | 6.50E-01 | -3.7 (-9.3; 1.9) | 1.96E-01 |
| PC ae C38:4 | -0.6 (-6.4; 5.2) | 8.43E-01 | 1.9 (-10.1; 13.9) | 7.56E-01 | -1.8 (-7.0; 3.4) | 4.94E-01 |
| PC ae C38:5 | 0.7 (-5.2; 6.5) | 8.26E-01 | 0.0 (-11.9; 11.9) | 9.97E-01 | 0.5 (-4.4; 5.5) | 8.32E-01 |
| PC ae C38:6 | 0.8 (-6.9; 8.5) | 8.35E-01 | -5.1 (-17.0; 6.9) | 4.02E-01 | 5.7 (0.2; 11.2) | 4.07E-02 |
| PC ae C40:0 | -0.5 (-7.2; 6.2) | 8.79E-01 | -1.6 (-15.7; 12.4) | 8.16E-01 | -1.1 (-6.5; 4.3) | 6.86E-01 |
| PC ae C40:1 | 0.5 (-6.1; 7.0) | 8.89E-01 | -4.5 (-20.7; 11.7) | 5.82E-01 | 1.6 (-3.2; 6.5) | 5.05E-01 |
| PC ae C40:2 | -1.1 (-7.6; 5.4) | 7.27E-01 | -2.5 (-18.2; 13.3) | 7.58E-01 | -1.0 (-6.4; 4.5) | 7.28E-01 |
| PC ae C40:3 | -8.8 (-16.6; -1.0) | 2.79E-02 | -16.4 (-35.5; 2.7) | 9.14E-02 | -5.5 (-12.5; 1.5) | 1.22E-01 |
| PC ae C40:4 | -5.2 (-11.1; 0.8) | 9.00E-02 | -7.1 (-20.0; 5.7) | 2.71E-01 | -3.9 (-9.0; 1.3) | 1.37E-01 |
| PC ae C40:5 | -2.7 (-8.3; 3.0) | 3.48E-01 | -3.1 (-16.1; 9.9) | 6.35E-01 | -2.3 (-7.5; 3.0) | 3.95E-01 |
| PC ae C40:6 | -3.7 (-11.0; 3.7) | 3.27E-01 | -9.0 (-21.7; 3.7) | 1.63E-01 | 0.3 (-5.1; 5.7) | 9.11E-01 |
| PC ae C42:0 | -0.2 (-7.2; 6.8) | 9.62E-01 | -3.7 (-17.7; 10.3) | 5.99E-01 | 0.8 (-5.4; 7.0) | 7.93E-01 |
| PC ae C42:1 | -0.5 (-6.2; 5.3) | 8.66E-01 | -4.0 (-18.0; 10.1) | 5.76E-01 | 0.9 (-5.0; 6.7) | 7.64E-01 |
| PC ae C42:2 | -5.5 (-12.5; 1.5) | 1.21E-01 | -11.9 (-26.5; 2.7) | 1.08E-01 | -3.3 (-8.9; 2.2) | 2.35E-01 |
| PC ae C42:3 | -6.5 (-14.4; 1.4) | 1.08E-01 | -11.6 (-27.7; 4.5) | 1.56E-01 | -1.9 (-7.7; 3.9) | 5.15E-01 |
| PC ae C42:4 | -10.1 (-16.6; -3.7) | 2.23E-03 | -13.2 (-24.5; -1.9) | 2.24E-02 | -6.2 (-12.9; 0.4) | 6.62E-02 |
| PC ae C42:5 | -6.6 (-12.9; -0.3) | 4.04E-02 | -8.9 (-22.4; 4.6) | 1.93E-01 | -4.2 (-9.7; 1.3) | 1.37E-01 |
| PC ae C44:3 | -3.3 (-11.2; 4.5) | 4.04E-01 | -8.5 (-24.5; 7.5) | 2.93E-01 | 0.0 (-6.7; 6.6) | 9.88E-01 |
| PC ae C44:4 | -6.6 (-12.6; -0.6) | 3.21E-02 | -8.4 (-17.9; 1.1) | 8.08E-02 | -4.3 (-10.3; 1.7) | 1.56E-01 |
| PC ae C44:5 | -4.1 (-10.5; 2.3) | 2.11E-01 | -2.5 (-16.4; 11.4) | 7.20E-01 | -2.6 (-8.2; 3.0) | 3.56E-01 |
| PC ae C44:6 | -9.2 (-15.2; -3.2) | 3.01E-03 | -10.3 (-22.1; 1.5) | 8.75E-02 | -5.6 (-11.4; 0.2) | 5.74E-02 |
| **Sphingomyelins** |  |  |  |  |  |  |
| SM (OH) C14:1 | -4.9 (-12.5; 2.6) | 1.99E-01 | -3.8 (-19.8; 12.1) | 6.33E-01 | -3.7 (-9.2; 1.8) | 1.86E-01 |
| SM (OH) C16:1 | -7.1 (-14.7; 0.4) | 6.32E-02 | -11.6 (-27.4; 4.2) | 1.50E-01 | -3.2 (-8.0; 1.5) | 1.77E-01 |
| SM (OH) C22:1 | -1.2 (-7.6; 5.1) | 7.04E-01 | 2.8 (-10.2; 15.7) | 6.73E-01 | -2.7 (-8.2; 2.9) | 3.44E-01 |
| SM (OH) C22:2 | -5.1 (-12.9; 2.7) | 2.02E-01 | -3.6 (-22.5; 15.3) | 7.07E-01 | -5.6 (-12.1; 1.0) | 9.54E-02 |
| SM (OH) C24:1 | -1.6 (-7.3; 4.0) | 5.71E-01 | 5.2 (-7.8; 18.1) | 4.28E-01 | -3.2 (-8.2; 1.8) | 2.08E-01 |
| SM C16:0 | -9.8 (-16.6; -3.1) | 4.67E-03 | -15.4 (-28.7; -2.0) | 2.45E-02 | -5.1 (-11.0; 0.8) | 8.69E-02 |
| SM C16:1 | -4.0 (-12.8; 4.8) | 3.76E-01 | -1.6 (-19.6; 16.4) | 8.64E-01 | -4.1 (-11.4; 3.3) | 2.76E-01 |
| SM C18:0 | -4.3 (-11.0; 2.3) | 1.98E-01 | -8.9 (-23.5; 5.7) | 2.28E-01 | -1.9 (-6.4; 2.7) | 4.20E-01 |
| SM C18:1 | -6.0 (-14.0; 2.0) | 1.39E-01 | -8.9 (-25.1; 7.3) | 2.78E-01 | -3.0 (-9.1; 3.0) | 3.23E-01 |
| SM C20:2 | -6.0 (-13.6; 1.6) | 1.22E-01 | -8.3 (-26.9; 10.3) | 3.78E-01 | -3.5 (-8.7; 1.7) | 1.81E-01 |
| SM C22:3 | -6.1 (-13.0; 0.8) | 8.12E-02 | -8.3 (-30.3; 13.8) | 4.57E-01 | -3.3 (-8.4; 1.8) | 1.96E-01 |
| SM C24:0 | -0.4 (-6.5; 5.7) | 8.97E-01 | 2.0 (-7.7; 11.7) | 6.87E-01 | -2.3 (-7.8; 3.1) | 3.91E-01 |
| SM C24:1 | -2.5 (-9.4; 4.3) | 4.67E-01 | -1.1 (-14.8; 12.5) | 8.71E-01 | -3.4 (-9.6; 2.8) | 2.77E-01 |
| SM C26:0 | -0.6 (-6.4; 5.3) | 8.48E-01 | 5.2 (-10.2; 20.6) | 5.04E-01 | -2.7 (-8.3; 2.9) | 3.46E-01 |
| SM C26:1 | -5.5 (-12.3; 1.4) | 1.20E-01 | -11.0 (-24.4; 2.5) | 1.08E-01 | 0.2 (-5.3; 5.7) | 9.49E-01 |
| **Amino acids** |  |  |  |  |  |  |
| Arginine | -3.2 (-10.9; 4.5) | 4.10E-01 | -6.6 (-21.8; 8.6) | 3.89E-01 | -2.4 (-7.8; 3.0) | 3.77E-01 |
| Glutamine | -4.2 (-11.8; 3.4) | 2.78E-01 | -11.8 (-28.5; 4.9) | 1.65E-01 | -1.6 (-7.6; 4.4) | 6.01E-01 |
| Glycine | -7.1 (-14.5; 0.3) | 5.95E-02 | -30.5 (-60.3; -0.8) | 4.45E-02 | -5.0 (-10.6; 0.6) | 8.21E-02 |
| Histidine | 1.6 (-4.9; 8.2) | 6.19E-01 | -5.5 (-20.9; 9.9) | 4.80E-01 | 2.9 (-1.4; 7.3) | 1.79E-01 |
| Methionine | 0.1 (-6.0; 6.2) | 9.77E-01 | -4.5 (-13.8; 4.8) | 3.35E-01 | 3.3 (-1.9; 8.5) | 2.07E-01 |
| Ornithine | -3.0 (-11.4; 5.4) | 4.81E-01 | -4.9 (-22.0; 12.2) | 5.68E-01 | -3.4 (-9.0; 2.1) | 2.22E-01 |
| Phenylalanine | 6.0 (0.5; 11.5) | 3.24E-02 | 3.6 (-5.9; 13.0) | 4.57E-01 | 4.9 (-0.7; 10.6) | 8.74E-02 |
| Proline | -3.8 (-8.7; 1.1) | 1.31E-01 | -3.2 (-8.8; 2.4) | 2.54E-01 | 0.4 (-7.3; 8.1) | 9.15E-01 |
| Serine | -6.3 (-13.9; 1.3) | 1.05E-01 | -8.9 (-31.1; 13.3) | 4.29E-01 | -4.3 (-9.6; 1.1) | 1.17E-01 |
| Threonine | -3.2 (-9.5; 3.0) | 3.13E-01 | -11.4 (-26.5; 3.6) | 1.35E-01 | 0.4 (-4.1; 4.9) | 8.58E-01 |
| Tryptophan | 2.8 (-3.3; 8.9) | 3.61E-01 | 4.9 (-5.7; 15.4) | 3.62E-01 | -0.2 (-5.9; 5.5) | 9.38E-01 |
| Tyrosine | 5.8 (-0.1; 11.6) | 5.37E-02 | 5.7 (-5.0; 16.4) | 2.94E-01 | 2.5 (-2.3; 7.3) | 3.00E-01 |
| Valine | 2.2 (-4.9; 9.3) | 5.45E-01 | 0.0 (-12.9; 13.0) | 9.97E-01 | 5.5 (-0.5; 11.5) | 6.97E-02 |
| (Iso)Leucine | 4.5 (-3.3; 12.3) | 2.53E-01 | 1.7 (-8.5; 11.8) | 7.45E-01 | 9.8 (1.6; 18.0) | 2.02E-02 |
| **Hexoses** |  |  |  |  |  |  |
| Hexoses | 4.7 (-1.9; 11.3) | 1.58E-01 | 5.5 (-4.8; 15.9) | 2.91E-01 | 4.8 (-0.9; 10.5) | 1.01E-01 |
| **Aggregate measures** |  |  |  |  |  |  |
| (C2 + C3) / C0 | -1.9 (-7.5; 3.6) | 4.99E-01 | -4.4 (-21.9; 13.2) | 6.23E-01 | -2.4 (-7.2; 2.3) | 3.16E-01 |
| Aromatic amino acids (AAA) | 5.6 (-0.4; 11.5) | 6.68E-02 | 5.6 (-4.3; 15.5) | 2.63E-01 | 2.6 (-3.0; 8.3) | 3.58E-01 |
| C2 / C0 | -1.9 (-7.5; 3.7) | 5.05E-01 | -4.2 (-21.7; 13.4) | 6.38E-01 | -2.5 (-7.2; 2.3) | 3.04E-01 |
| (C16 + C18) / C0 | -0.2 (-6.1; 5.7) | 9.50E-01 | -3.7 (-15.5; 8.0) | 5.30E-01 | 1.4 (-5.2; 8.0) | 6.78E-01 |
| MUFA(PC) | 4.3 (-1.7; 10.2) | 1.57E-01 | 6.9 (-4.1; 17.8) | 2.15E-01 | 0.0 (-5.1; 5.2) | 9.91E-01 |
| MUFA(PC) / SFA(PC) | 5.6 (-0.9; 12.0) | 8.92E-02 | 13.6 (2.5; 24.7) | 1.68E-02 | -0.8 (-5.6; 4.0) | 7.48E-01 |
| Ornithine / Arginine | -0.1 (-9.1; 8.8) | 9.77E-01 | -0.1 (-15.3; 15.1) | 9.92E-01 | -0.6 (-6.6; 5.5) | 8.55E-01 |
| PUFA(PC) | 5.0 (-0.4; 10.4) | 6.84E-02 | 6.0 (-3.6; 15.6) | 2.20E-01 | 2.8 (-2.3; 7.8) | 2.75E-01 |
| PUFA(PC) / MUFA(PC) | -1.1 (-7.3; 5.1) | 7.23E-01 | -3.5 (-14.1; 7.0) | 5.07E-01 | 2.3 (-3.9; 8.5) | 4.59E-01 |
| PUFA(PC) / SFA(PC) | 5.0 (-2.4; 12.4) | 1.82E-01 | 6.7 (-5.3; 18.7) | 2.70E-01 | 2.5 (-3.8; 8.8) | 4.37E-01 |
| SFA(PC) | 0.4 (-7.3; 8.1) | 9.20E-01 | -4.1 (-16.0; 7.7) | 4.92E-01 | 1.8 (-5.5; 9.0) | 6.29E-01 |
| Total PC + Total SM | 3.3 (-2.2; 8.9) | 2.35E-01 | 3.9 (-6.0; 13.7) | 4.38E-01 | 1.3 (-4.1; 6.6) | 6.42E-01 |
| Total AC / C0 | -2.2 (-7.5; 3.0) | 3.99E-01 | -8.7 (-25.3; 7.8) | 2.98E-01 | -1.9 (-6.7; 2.9) | 4.26E-01 |
| Total AC-DC / Total AC | 3.0 (-3.5; 9.6) | 3.57E-01 | 6.1 (-7.9; 20.1) | 3.88E-01 | 1.5 (-4.3; 7.3) | 6.08E-01 |
| Total AC-OH / Total AC | 6.6 (0.9; 12.4) | 2.41E-02 | 15.8 (2.1; 29.6) | 2.47E-02 | 2.7 (-2.0; 7.4) | 2.55E-01 |
| Total lysoPC | -8.9 (-16.7; -1.1) | 2.61E-02 | -15.4 (-30.3; -0.5) | 4.23E-02 | -3.8 (-9.6; 2.0) | 2.00E-01 |
| Total lysoPC / Total PC | -15.5 (-23.0; -8.0)* | 6.85E-05 | -25.4 (-41.4; -9.5) | 2.16E-03 | -6.2 (-11.0; -1.4) | 1.18E-02 |
| Total PC | 5.0 (-0.5; 10.5) | 7.58E-02 | 6.3 (-3.3; 16.0) | 1.96E-01 | 2.2 (-2.9; 7.4) | 3.88E-01 |
| Total diacyl PC | 5.6 (0.1; 11.2) | 4.48E-02 | 7.7 (-2.0; 17.4) | 1.18E-01 | 2.5 (-2.5; 7.6) | 3.23E-01 |
| Total acyl-alkyl PC | -3.3 (-10.3; 3.7) | 3.52E-01 | -8.2 (-23.5; 7.1) | 2.90E-01 | -1.2 (-7.3; 4.8) | 6.82E-01 |
| Total SM | -6.2 (-12.7; 0.4) | 6.55E-02 | -8.3 (-22.5; 5.9) | 2.51E-01 | -4.4 (-10.3; 1.5) | 1.42E-01 |
| Total SM / (Total SM + Total PC) | -12.1 (-17.8; -6.3)* | 5.01E-05 | -13.9 (-24.1; -3.7) | 8.02E-03 | -7.2 (-12.2; -2.2) | 4.96E-03 |
| Total SM / Total PC | -12.1 (-17.8; -6.4)* | 4.89E-05 | -14.0 (-24.2; -3.8) | 7.59E-03 | -7.2 (-12.1; -2.2) | 5.03E-03 |
| Total SM-non OH | -6.3 (-12.8; 0.2) | 5.57E-02 | -8.7 (-22.2; 4.8) | 2.03E-01 | -4.3 (-10.2; 1.6) | 1.52E-01 |
| Total SM-OH | -4.1 (-11.3; 3.1) | 2.66E-01 | -2.3 (-19.5; 14.9) | 7.91E-01 | -4.3 (-10.1; 1.5) | 1.43E-01 |
| Total SM-OH / Total SM-non OH | 2.2 (-4.7; 9.1) | 5.33E-01 | 7.3 (-3.7; 18.3) | 1.90E-01 | -0.8 (-6.1; 4.5) | 7.64E-01 |
| Tyrosine / Phenylalanine | 2.6 (-3.3; 8.5) | 3.85E-01 | 4.1 (-9.0; 17.1) | 5.39E-01 | -0.3 (-4.6; 3.9) | 8.71E-01 |
|  | **Hepatic triglyceride content** | | | | | |
|  | Total |  | Men |  | Women |  |
|  | (N = 174) |  | (N = 84) |  | (N = 90) |  |
|  | Estimate (95% CI) | P-value | Estimate (95% CI) | P-value | Estimate (95% CI) | P-value |
| **Acylcarnitines** |  |  |  |  |  |  |
| C0 | 1.14 (0.99; 1.31) | 7.09E-02 | 1.04 (0.83; 1.29) | 7.44E-01 | 1.17 (0.96; 1.42) | 1.17E-01 |
| C2 | 0.94 (0.81; 1.10) | 4.48E-01 | 0.99 (0.77; 1.28) | 9.56E-01 | 0.95 (0.80; 1.13) | 5.77E-01 |
| C3 | 1.08 (0.92; 1.25) | 3.50E-01 | 1.07 (0.88; 1.31) | 4.95E-01 | 1.07 (0.83; 1.37) | 6.13E-01 |
| C3-DC (C4-OH) | 0.96 (0.85; 1.08) | 4.70E-01 | 0.98 (0.79; 1.22) | 8.85E-01 | 0.96 (0.84; 1.10) | 5.66E-01 |
| C3-OH | 1.05 (0.92; 1.20) | 4.65E-01 | 1.05 (0.87; 1.26) | 5.86E-01 | 1.05 (0.86; 1.28) | 6.53E-01 |
| C3:1 | 1.02 (0.89; 1.16) | 7.89E-01 | 1.06 (0.85; 1.32) | 6.10E-01 | 0.98 (0.85; 1.14) | 8.29E-01 |
| C4 | 1.08 (0.97; 1.21) | 1.63E-01 | 1.11 (1.00; 1.23) | 6.14E-02 | 1.02 (0.78; 1.33) | 9.10E-01 |
| C4:1 | 1.08 (0.92; 1.25) | 3.44E-01 | 1.02 (0.82; 1.27) | 8.73E-01 | 1.12 (0.95; 1.31) | 1.64E-01 |
| C5 | 1.06 (0.91; 1.24) | 4.37E-01 | 1.05 (0.81; 1.35) | 7.12E-01 | 1.07 (0.90; 1.27) | 4.30E-01 |
| C5-DC (C6-OH) | 1.01 (0.86; 1.19) | 8.79E-01 | 0.97 (0.81; 1.16) | 7.17E-01 | 1.05 (0.88; 1.25) | 5.59E-01 |
| C5-M-DC | 0.94 (0.82; 1.07) | 3.16E-01 | 1.08 (0.91; 1.29) | 3.55E-01 | 0.87 (0.75; 1.01) | 7.15E-02 |
| C5-OH (C3-DC-M) | 1.18 (1.04; 1.35) | 1.09E-02 | 1.10 (0.92; 1.30) | 2.98E-01 | 1.18 (0.99; 1.41) | 6.24E-02 |
| C5:1 | 0.94 (0.83; 1.06) | 3.22E-01 | 1.02 (0.79; 1.33) | 8.72E-01 | 0.89 (0.77; 1.04) | 1.43E-01 |
| C5:1-DC | 1.04 (0.90; 1.19) | 6.30E-01 | 0.88 (0.76; 1.01) | 7.02E-02 | 1.20 (0.99; 1.44) | 5.67E-02 |
| C6 (C4:1-DC) | 1.01 (0.86; 1.17) | 9.39E-01 | 0.96 (0.85; 1.08) | 4.96E-01 | 1.19 (0.94; 1.50) | 1.51E-01 |
| C6:1 | 1.12 (0.99; 1.27) | 7.79E-02 | 1.05 (0.87; 1.26) | 5.89E-01 | 1.20 (1.02; 1.40) | 2.67E-02 |
| C7-DC | 0.92 (0.79; 1.07) | 2.63E-01 | 1.00 (0.78; 1.28) | 9.96E-01 | 0.89 (0.76; 1.05) | 1.66E-01 |
| C8 | 0.93 (0.81; 1.07) | 2.99E-01 | 0.90 (0.84; 0.98) | 9.92E-03 | 1.22 (0.85; 1.76) | 2.76E-01 |
| C8:1 | 1.13 (1.01; 1.27) | 3.33E-02 | 1.13 (0.92; 1.39) | 2.54E-01 | 1.14 (1.01; 1.27) | 2.80E-02 |
| C9 | 1.09 (0.97; 1.23) | 1.52E-01 | 1.13 (0.93; 1.37) | 2.18E-01 | 1.01 (0.87; 1.18) | 8.81E-01 |
| C10 | 0.92 (0.80; 1.06) | 2.65E-01 | 0.90 (0.84; 0.96) | 3.77E-03 | 1.15 (0.81; 1.65) | 4.28E-01 |
| C10:1 | 0.94 (0.81; 1.10) | 4.42E-01 | 0.90 (0.77; 1.06) | 2.01E-01 | 1.11 (0.88; 1.40) | 3.76E-01 |
| C10:2 | 1.02 (0.87; 1.21) | 7.84E-01 | 1.00 (0.80; 1.24) | 9.67E-01 | 1.03 (0.86; 1.24) | 7.66E-01 |
| C12 | 0.93 (0.78; 1.10) | 3.77E-01 | 0.90 (0.79; 1.02) | 1.05E-01 | 1.13 (0.84; 1.53) | 4.08E-01 |
| C12-DC | 0.92 (0.80; 1.06) | 2.59E-01 | 1.07 (0.86; 1.33) | 5.65E-01 | 0.89 (0.74; 1.07) | 2.00E-01 |
| C12:1 | 0.86 (0.71; 1.04) | 1.16E-01 | 0.80 (0.66; 0.96) | 1.73E-02 | 1.03 (0.78; 1.36) | 8.53E-01 |
| C14 | 1.04 (0.89; 1.21) | 6.46E-01 | 1.00 (0.88; 1.14) | 9.67E-01 | 1.24 (0.98; 1.57) | 7.35E-02 |
| C14:1 | 0.86 (0.74; 0.99) | 3.62E-02 | 0.87 (0.69; 1.09) | 2.31E-01 | 0.98 (0.78; 1.24) | 8.83E-01 |
| C14:1-OH | 0.92 (0.81; 1.05) | 2.31E-01 | 0.95 (0.81; 1.11) | 5.07E-01 | 0.95 (0.80; 1.13) | 5.89E-01 |
| C14:2 | 0.83 (0.72; 0.96) | 1.04E-02 | 0.89 (0.71; 1.11) | 2.84E-01 | 0.90 (0.72; 1.12) | 3.54E-01 |
| C14:2-OH | 0.98 (0.87; 1.11) | 7.80E-01 | 1.07 (0.90; 1.28) | 4.31E-01 | 0.98 (0.87; 1.10) | 6.84E-01 |
| C16 | 1.16 (1.01; 1.32) | 3.38E-02 | 1.02 (0.88; 1.17) | 8.00E-01 | 1.26 (1.10; 1.45) | 1.29E-03 |
| C16-OH | 1.02 (0.88; 1.18) | 8.06E-01 | 1.17 (0.92; 1.50) | 1.99E-01 | 0.90 (0.76; 1.06) | 2.03E-01 |
| C16:1 | 0.91 (0.78; 1.05) | 1.97E-01 | 0.87 (0.73; 1.04) | 1.17E-01 | 1.01 (0.81; 1.27) | 8.95E-01 |
| C16:1-OH | 1.12 (0.98; 1.28) | 9.39E-02 | 1.14 (0.92; 1.42) | 2.39E-01 | 1.11 (0.95; 1.28) | 1.87E-01 |
| C16:2 | 0.89 (0.77; 1.04) | 1.47E-01 | 0.90 (0.70; 1.15) | 3.78E-01 | 1.00 (0.84; 1.20) | 9.65E-01 |
| C16:2-OH | 1.08 (0.93; 1.25) | 3.05E-01 | 0.95 (0.72; 1.25) | 7.19E-01 | 1.11 (0.96; 1.28) | 1.69E-01 |
| C18 | 1.12 (0.99; 1.27) | 7.49E-02 | 1.05 (0.90; 1.23) | 5.04E-01 | 1.14 (0.96; 1.37) | 1.40E-01 |
| C18:1 | 0.96 (0.82; 1.12) | 6.05E-01 | 0.89 (0.74; 1.08) | 2.31E-01 | 1.06 (0.87; 1.31) | 5.47E-01 |
| C18:1-OH | 1.06 (0.96; 1.18) | 2.58E-01 | 1.06 (0.69; 1.62) | 7.78E-01 | 1.06 (0.95; 1.18) | 2.76E-01 |
| C18:2 | 0.94 (0.80; 1.09) | 3.95E-01 | 0.91 (0.71; 1.16) | 4.43E-01 | 1.02 (0.83; 1.24) | 8.76E-01 |
| **Lysophosphatidylcholines** |  |  |  |  |  |  |
| Lyso PC a C6:0 | 1.07 (0.92; 1.24) | 3.71E-01 | 0.91 (0.76; 1.10) | 3.38E-01 | 1.11 (0.91; 1.37) | 2.98E-01 |
| Lyso PC a C14:0 | 1.30 (1.17; 1.45)* | 3.64E-06 | 1.33 (1.15; 1.54) | 2.02E-04 | 1.20 (1.04; 1.39) | 1.57E-02 |
| Lyso PC a C16:0 | 0.98 (0.86; 1.11) | 7.26E-01 | 0.96 (0.82; 1.13) | 6.15E-01 | 0.90 (0.76; 1.06) | 1.92E-01 |
| Lyso PC a C16:1 | 1.13 (1.01; 1.27) | 2.65E-02 | 1.11 (0.93; 1.32) | 2.55E-01 | 1.10 (0.97; 1.26) | 1.38E-01 |
| Lyso PC a C17:0 | 0.88 (0.76; 1.02) | 8.27E-02 | 0.89 (0.74; 1.07) | 2.12E-01 | 0.79 (0.66; 0.95) | 1.15E-02 |
| Lyso PC a C18:0 | 1.04 (0.92; 1.17) | 5.02E-01 | 0.93 (0.75; 1.15) | 5.03E-01 | 1.02 (0.90; 1.15) | 7.88E-01 |
| Lyso PC a C18:1 | 0.94 (0.82; 1.07) | 3.49E-01 | 0.79 (0.65; 0.95) | 1.38E-02 | 1.01 (0.86; 1.19) | 8.81E-01 |
| Lyso PC a C18:2 | 0.92 (0.80; 1.05) | 2.23E-01 | 0.85 (0.71; 1.02) | 7.65E-02 | 1.00 (0.81; 1.23) | 1.00E+00 |
| Lyso PC a C20:3 | 1.09 (0.94; 1.26) | 2.38E-01 | 0.94 (0.76; 1.15) | 5.35E-01 | 1.19 (0.99; 1.42) | 6.12E-02 |
| Lyso PC a C20:4 | 0.92 (0.79; 1.07) | 2.70E-01 | 0.80 (0.67; 0.95) | 1.40E-02 | 0.99 (0.79; 1.24) | 9.09E-01 |
| Lyso PC a C24:0 | 0.96 (0.86; 1.07) | 4.20E-01 | 1.00 (0.83; 1.21) | 9.87E-01 | 0.95 (0.83; 1.08) | 3.92E-01 |
| Lyso PC a C26:0 | 0.98 (0.88; 1.09) | 6.67E-01 | 1.13 (0.94; 1.36) | 1.82E-01 | 0.94 (0.82; 1.08) | 3.73E-01 |
| Lyso PC a C26:1 | 0.94 (0.85; 1.05) | 2.84E-01 | 1.05 (0.87; 1.27) | 6.10E-01 | 0.93 (0.82; 1.05) | 2.29E-01 |
| Lyso PC a C28:0 | 1.01 (0.91; 1.13) | 8.11E-01 | 1.18 (1.01; 1.39) | 3.85E-02 | 0.97 (0.85; 1.10) | 6.08E-01 |
| Lyso PC a C28:1 | 1.00 (0.89; 1.13) | 9.63E-01 | 1.17 (0.93; 1.47) | 1.82E-01 | 0.94 (0.82; 1.08) | 3.69E-01 |
| **Diacyl phosphatidylcholines** |  |  |  |  |  |  |
| PC aa C24:0 | 1.02 (0.91; 1.14) | 7.29E-01 | 1.13 (0.93; 1.38) | 2.24E-01 | 0.97 (0.85; 1.11) | 6.75E-01 |
| PC aa C26:0 | 1.00 (0.90; 1.12) | 9.38E-01 | 1.15 (0.96; 1.37) | 1.38E-01 | 0.96 (0.84; 1.09) | 5.39E-01 |
| PC aa C28:1 | 1.23 (1.07; 1.41)* | 4.50E-03 | 1.10 (0.87; 1.38) | 4.20E-01 | 1.18 (0.98; 1.44) | 8.59E-02 |
| PC aa C30:0 | 1.30 (1.14; 1.47)* | 9.32E-05 | 1.34 (1.11; 1.62) | 3.37E-03 | 1.19 (1.01; 1.40) | 4.16E-02 |
| PC aa C30:2 | 1.04 (0.89; 1.21) | 6.28E-01 | 1.05 (0.85; 1.29) | 6.48E-01 | 1.07 (0.87; 1.32) | 4.99E-01 |
| PC aa C32:0 | 1.13 (1.00; 1.28) | 5.39E-02 | 1.04 (0.86; 1.24) | 6.93E-01 | 1.13 (0.97; 1.32) | 1.27E-01 |
| PC aa C32:1 | 1.38 (1.23; 1.55)* | 9.12E-08 | 1.37 (1.12; 1.68) | 2.82E-03 | 1.31 (1.13; 1.52) | 4.03E-04 |
| PC aa C32:2 | 1.32 (1.17; 1.49)* | 2.05E-05 | 1.41 (1.18; 1.68) | 2.48E-04 | 1.23 (1.05; 1.44) | 1.16E-02 |
| PC aa C32:3 | 1.20 (1.02; 1.41) | 2.77E-02 | 1.16 (0.94; 1.44) | 1.72E-01 | 1.16 (0.95; 1.42) | 1.35E-01 |
| PC aa C34:1 | 1.22 (1.08; 1.38)* | 1.26E-03 | 1.14 (0.92; 1.41) | 2.24E-01 | 1.18 (1.00; 1.38) | 4.54E-02 |
| PC aa C34:2 | 1.17 (1.03; 1.33) | 1.60E-02 | 1.11 (0.87; 1.42) | 3.97E-01 | 1.09 (0.92; 1.30) | 2.94E-01 |
| PC aa C34:3 | 1.33 (1.16; 1.52)* | 6.96E-05 | 1.33 (1.05; 1.68) | 1.86E-02 | 1.25 (1.06; 1.47) | 7.80E-03 |
| PC aa C34:4 | 1.36 (1.20; 1.53)* | 1.84E-06 | 1.36 (1.09; 1.70) | 7.56E-03 | 1.28 (1.10; 1.49) | 1.42E-03 |
| PC aa C36:0 | 0.96 (0.84; 1.10) | 5.97E-01 | 0.89 (0.77; 1.03) | 1.05E-01 | 1.08 (0.90; 1.29) | 4.15E-01 |
| PC aa C36:1 | 1.35 (1.20; 1.52)* | 1.04E-06 | 1.29 (0.93; 1.78) | 1.26E-01 | 1.30 (1.15; 1.46) | 4.09E-05 |
| PC aa C36:2 | 1.26 (1.11; 1.43)* | 5.55E-04 | 1.14 (0.88; 1.47) | 3.27E-01 | 1.23 (1.05; 1.43) | 9.10E-03 |
| PC aa C36:3 | 1.21 (1.06; 1.37)* | 3.77E-03 | 1.11 (0.86; 1.42) | 4.22E-01 | 1.16 (0.97; 1.38) | 9.69E-02 |
| PC aa C36:4 | 1.09 (0.96; 1.25) | 1.82E-01 | 1.00 (0.81; 1.24) | 9.71E-01 | 1.07 (0.90; 1.26) | 4.52E-01 |
| PC aa C36:5 | 1.15 (1.00; 1.34) | 5.57E-02 | 1.05 (0.83; 1.33) | 6.69E-01 | 1.19 (1.01; 1.40) | 3.98E-02 |
| PC aa C36:6 | 1.24 (1.06; 1.44)* | 6.68E-03 | 1.18 (0.92; 1.52) | 1.95E-01 | 1.18 (0.96; 1.46) | 1.13E-01 |
| PC aa C38:0 | 0.94 (0.81; 1.08) | 3.65E-01 | 0.85 (0.70; 1.03) | 1.02E-01 | 1.02 (0.86; 1.20) | 8.10E-01 |
| PC aa C38:1 | 0.97 (0.85; 1.11) | 6.65E-01 | 1.02 (0.83; 1.24) | 8.73E-01 | 0.96 (0.83; 1.13) | 6.42E-01 |
| PC aa C38:3 | 1.41 (1.26; 1.59)* | 2.05E-08 | 1.43 (1.12; 1.82) | 4.48E-03 | 1.32 (1.14; 1.54) | 4.61E-04 |
| PC aa C38:4 | 1.17 (1.04; 1.32) | 9.40E-03 | 1.04 (0.85; 1.26) | 7.00E-01 | 1.17 (1.01; 1.36) | 4.14E-02 |
| PC aa C38:5 | 1.20 (1.06; 1.36)* | 5.36E-03 | 1.05 (0.84; 1.32) | 6.46E-01 | 1.21 (1.04; 1.42) | 1.74E-02 |
| PC aa C38:6 | 1.03 (0.87; 1.21) | 7.45E-01 | 0.99 (0.78; 1.26) | 9.47E-01 | 0.97 (0.80; 1.17) | 7.26E-01 |
| PC aa C40:1 | 0.94 (0.81; 1.09) | 3.97E-01 | 0.90 (0.72; 1.11) | 3.13E-01 | 0.99 (0.85; 1.16) | 9.42E-01 |
| PC aa C40:2 | 0.99 (0.86; 1.15) | 8.93E-01 | 0.90 (0.72; 1.13) | 3.47E-01 | 1.06 (0.91; 1.23) | 4.46E-01 |
| PC aa C40:3 | 1.02 (0.88; 1.20) | 7.58E-01 | 0.93 (0.74; 1.17) | 5.57E-01 | 1.03 (0.87; 1.23) | 7.12E-01 |
| PC aa C40:4 | 1.23 (1.10; 1.38)* | 3.25E-04 | 1.13 (0.91; 1.40) | 2.62E-01 | 1.19 (1.04; 1.38) | 1.40E-02 |
| PC aa C40:5 | 1.36 (1.21; 1.52)* | 6.78E-07 | 1.30 (1.04; 1.63) | 2.40E-02 | 1.32 (1.14; 1.53) | 3.65E-04 |
| PC aa C40:6 | 1.19 (1.03; 1.38) | 1.73E-02 | 1.14 (0.89; 1.46) | 2.86E-01 | 1.12 (0.95; 1.33) | 1.74E-01 |
| PC aa C42:0 | 0.89 (0.79; 1.02) | 8.86E-02 | 0.80 (0.68; 0.95) | 9.63E-03 | 0.97 (0.83; 1.14) | 7.45E-01 |
| PC aa C42:1 | 0.91 (0.80; 1.04) | 1.61E-01 | 0.78 (0.66; 0.94) | 7.66E-03 | 0.97 (0.84; 1.12) | 6.70E-01 |
| PC aa C42:2 | 0.95 (0.83; 1.09) | 4.44E-01 | 0.87 (0.71; 1.06) | 1.67E-01 | 1.03 (0.87; 1.21) | 7.28E-01 |
| PC aa C42:4 | 0.98 (0.88; 1.10) | 7.89E-01 | 0.82 (0.66; 1.03) | 9.04E-02 | 0.99 (0.87; 1.13) | 8.79E-01 |
| PC aa C42:5 | 1.11 (0.94; 1.31) | 2.18E-01 | 1.06 (0.84; 1.33) | 6.20E-01 | 1.08 (0.88; 1.33) | 4.61E-01 |
| PC aa C42:6 | 1.05 (0.90; 1.24) | 5.22E-01 | 1.01 (0.77; 1.33) | 9.24E-01 | 0.96 (0.80; 1.17) | 6.94E-01 |
| **Acyl-alkyl phosphatidylcholines** |  |  |  |  |  |  |
| PC ae C30:0 | 1.13 (0.99; 1.29) | 7.82E-02 | 1.14 (0.95; 1.37) | 1.68E-01 | 1.06 (0.89; 1.26) | 5.20E-01 |
| PC ae C30:1 | 1.01 (0.91; 1.13) | 8.06E-01 | 1.04 (0.86; 1.26) | 6.86E-01 | 1.00 (0.89; 1.13) | 9.54E-01 |
| PC ae C30:2 | 1.06 (0.93; 1.21) | 4.03E-01 | 1.04 (0.86; 1.24) | 6.94E-01 | 1.05 (0.89; 1.23) | 5.78E-01 |
| PC ae C32:1 | 1.07 (0.93; 1.22) | 3.36E-01 | 0.89 (0.72; 1.08) | 2.36E-01 | 1.14 (0.96; 1.34) | 1.26E-01 |
| PC ae C32:2 | 1.00 (0.87; 1.15) | 9.79E-01 | 0.81 (0.69; 0.95) | 1.10E-02 | 1.10 (0.93; 1.31) | 2.56E-01 |
| PC ae C34:0 | 1.14 (0.99; 1.32) | 6.84E-02 | 1.17 (0.94; 1.47) | 1.63E-01 | 1.03 (0.87; 1.22) | 7.38E-01 |
| PC ae C34:1 | 1.07 (0.94; 1.23) | 3.04E-01 | 0.99 (0.81; 1.21) | 9.25E-01 | 1.07 (0.91; 1.26) | 3.87E-01 |
| PC ae C34:2 | 1.01 (0.87; 1.18) | 8.95E-01 | 0.91 (0.73; 1.13) | 3.85E-01 | 1.05 (0.87; 1.27) | 6.08E-01 |
| PC ae C34:3 | 1.02 (0.88; 1.20) | 7.54E-01 | 0.76 (0.58; 1.00) | 5.24E-02 | 1.16 (1.00; 1.35) | 5.41E-02 |
| PC ae C36:0 | 1.05 (0.90; 1.22) | 5.41E-01 | 0.94 (0.78; 1.14) | 5.43E-01 | 1.12 (0.92; 1.37) | 2.46E-01 |
| PC ae C36:1 | 1.15 (0.99; 1.33) | 6.12E-02 | 1.12 (0.87; 1.45) | 3.69E-01 | 1.04 (0.86; 1.25) | 6.96E-01 |
| PC ae C36:2 | 0.92 (0.79; 1.07) | 2.80E-01 | 0.92 (0.76; 1.11) | 3.91E-01 | 0.86 (0.71; 1.03) | 9.15E-02 |
| PC ae C36:3 | 1.04 (0.89; 1.22) | 5.76E-01 | 0.87 (0.69; 1.11) | 2.68E-01 | 1.11 (0.92; 1.34) | 2.79E-01 |
| PC ae C36:4 | 1.09 (0.93; 1.27) | 2.72E-01 | 0.89 (0.71; 1.11) | 2.94E-01 | 1.15 (0.97; 1.38) | 1.14E-01 |
| PC ae C36:5 | 1.06 (0.92; 1.23) | 4.09E-01 | 0.80 (0.66; 0.96) | 1.79E-02 | 1.21 (1.05; 1.39) | 9.83E-03 |
| PC ae C38:0 | 1.08 (0.91; 1.28) | 3.70E-01 | 0.96 (0.74; 1.25) | 7.85E-01 | 1.05 (0.83; 1.32) | 6.95E-01 |
| PC ae C38:1 | 0.94 (0.82; 1.07) | 3.40E-01 | 0.97 (0.83; 1.14) | 7.28E-01 | 0.91 (0.76; 1.10) | 3.35E-01 |
| PC ae C38:2 | 1.00 (0.87; 1.16) | 9.60E-01 | 0.97 (0.81; 1.17) | 7.58E-01 | 0.97 (0.80; 1.17) | 7.38E-01 |
| PC ae C38:3 | 1.08 (0.94; 1.24) | 2.87E-01 | 1.03 (0.80; 1.31) | 8.34E-01 | 0.97 (0.80; 1.17) | 7.45E-01 |
| PC ae C38:4 | 1.00 (0.89; 1.13) | 9.64E-01 | 0.91 (0.72; 1.14) | 3.95E-01 | 0.97 (0.85; 1.11) | 6.65E-01 |
| PC ae C38:5 | 1.00 (0.87; 1.15) | 9.63E-01 | 0.80 (0.66; 0.98) | 3.31E-02 | 1.09 (0.94; 1.27) | 2.53E-01 |
| PC ae C38:6 | 1.03 (0.89; 1.21) | 6.67E-01 | 0.89 (0.73; 1.09) | 2.57E-01 | 1.15 (0.97; 1.38) | 1.05E-01 |
| PC ae C40:0 | 0.90 (0.78; 1.04) | 1.57E-01 | 0.77 (0.61; 0.95) | 1.80E-02 | 0.88 (0.73; 1.06) | 1.81E-01 |
| PC ae C40:1 | 1.03 (0.89; 1.20) | 6.79E-01 | 0.89 (0.73; 1.09) | 2.72E-01 | 0.99 (0.82; 1.19) | 8.77E-01 |
| PC ae C40:2 | 0.99 (0.86; 1.13) | 8.38E-01 | 0.97 (0.81; 1.15) | 6.88E-01 | 0.90 (0.75; 1.07) | 2.37E-01 |
| PC ae C40:3 | 0.96 (0.83; 1.11) | 5.80E-01 | 0.87 (0.71; 1.07) | 1.89E-01 | 0.93 (0.80; 1.10) | 4.09E-01 |
| PC ae C40:4 | 0.99 (0.88; 1.11) | 8.86E-01 | 0.88 (0.71; 1.09) | 2.25E-01 | 1.00 (0.88; 1.13) | 9.45E-01 |
| PC ae C40:5 | 0.98 (0.86; 1.11) | 7.18E-01 | 0.80 (0.67; 0.95) | 1.34E-02 | 1.01 (0.87; 1.16) | 9.17E-01 |
| PC ae C40:6 | 0.93 (0.79; 1.08) | 3.18E-01 | 0.88 (0.70; 1.10) | 2.55E-01 | 0.89 (0.74; 1.06) | 1.95E-01 |
| PC ae C42:0 | 0.87 (0.76; 0.98) | 2.59E-02 | 0.83 (0.66; 1.05) | 1.16E-01 | 0.85 (0.75; 0.98) | 2.46E-02 |
| PC ae C42:1 | 1.04 (0.92; 1.16) | 5.34E-01 | 0.92 (0.75; 1.13) | 4.28E-01 | 1.00 (0.87; 1.16) | 9.54E-01 |
| PC ae C42:2 | 1.05 (0.92; 1.20) | 4.33E-01 | 0.90 (0.72; 1.12) | 3.26E-01 | 1.04 (0.90; 1.21) | 5.75E-01 |
| PC ae C42:3 | 0.91 (0.80; 1.02) | 1.07E-01 | 0.83 (0.69; 1.01) | 6.25E-02 | 0.92 (0.80; 1.07) | 2.90E-01 |
| PC ae C42:4 | 0.96 (0.85; 1.08) | 4.60E-01 | 0.89 (0.73; 1.09) | 2.60E-01 | 1.00 (0.86; 1.15) | 9.55E-01 |
| PC ae C42:5 | 0.93 (0.81; 1.05) | 2.39E-01 | 0.75 (0.63; 0.90) | 2.37E-03 | 1.02 (0.89; 1.17) | 7.42E-01 |
| PC ae C44:3 | 1.06 (0.93; 1.20) | 3.84E-01 | 1.00 (0.80; 1.24) | 9.81E-01 | 1.04 (0.91; 1.20) | 5.34E-01 |
| PC ae C44:4 | 1.02 (0.91; 1.15) | 7.12E-01 | 0.90 (0.73; 1.10) | 2.90E-01 | 1.12 (0.99; 1.26) | 7.00E-02 |
| PC ae C44:5 | 0.96 (0.85; 1.10) | 5.88E-01 | 0.83 (0.65; 1.06) | 1.31E-01 | 1.07 (0.94; 1.20) | 3.04E-01 |
| PC ae C44:6 | 0.89 (0.78; 1.01) | 7.85E-02 | 0.83 (0.65; 1.05) | 1.22E-01 | 0.98 (0.83; 1.14) | 7.64E-01 |
| **Sphingomyelins** |  |  |  |  |  |  |
| SM (OH) C14:1 | 1.00 (0.85; 1.17) | 9.89E-01 | 0.91 (0.74; 1.13) | 4.02E-01 | 0.97 (0.78; 1.19) | 7.47E-01 |
| SM (OH) C16:1 | 0.94 (0.81; 1.08) | 3.56E-01 | 0.88 (0.72; 1.06) | 1.76E-01 | 0.90 (0.75; 1.07) | 2.26E-01 |
| SM (OH) C22:1 | 1.19 (1.04; 1.37) | 1.04E-02 | 1.09 (0.88; 1.35) | 4.04E-01 | 1.17 (0.99; 1.39) | 7.25E-02 |
| SM (OH) C22:2 | 1.02 (0.87; 1.19) | 7.98E-01 | 1.00 (0.79; 1.27) | 9.81E-01 | 0.94 (0.79; 1.13) | 5.22E-01 |
| SM (OH) C24:1 | 1.12 (0.98; 1.27) | 8.72E-02 | 1.02 (0.83; 1.25) | 8.32E-01 | 1.08 (0.93; 1.27) | 3.07E-01 |
| SM C16:0 | 0.96 (0.85; 1.09) | 5.39E-01 | 0.76 (0.64; 0.91) | 3.66E-03 | 1.04 (0.90; 1.21) | 5.64E-01 |
| SM C16:1 | 1.01 (0.87; 1.17) | 9.21E-01 | 0.91 (0.72; 1.15) | 4.27E-01 | 1.03 (0.88; 1.21) | 7.12E-01 |
| SM C18:0 | 1.04 (0.92; 1.18) | 5.22E-01 | 0.96 (0.80; 1.16) | 6.98E-01 | 1.00 (0.88; 1.15) | 9.54E-01 |
| SM C18:1 | 0.95 (0.83; 1.09) | 4.53E-01 | 0.95 (0.78; 1.16) | 6.08E-01 | 0.90 (0.77; 1.05) | 1.82E-01 |
| SM C20:2 | 0.86 (0.74; 1.00) | 4.34E-02 | 0.99 (0.80; 1.23) | 9.60E-01 | 0.79 (0.66; 0.94) | 9.17E-03 |
| SM C22:3 | 0.84 (0.75; 0.94)* | 2.36E-03 | 0.93 (0.74; 1.18) | 5.61E-01 | 0.85 (0.76; 0.97) | 1.20E-02 |
| SM C24:0 | 1.17 (1.04; 1.32) | 1.07E-02 | 1.00 (0.83; 1.21) | 9.73E-01 | 1.23 (1.05; 1.44) | 1.02E-02 |
| SM C24:1 | 0.98 (0.86; 1.10) | 6.92E-01 | 0.89 (0.74; 1.07) | 2.18E-01 | 1.01 (0.87; 1.16) | 9.31E-01 |
| SM C26:0 | 1.02 (0.90; 1.15) | 7.81E-01 | 0.99 (0.77; 1.26) | 9.05E-01 | 0.96 (0.85; 1.08) | 4.71E-01 |
| SM C26:1 | 0.99 (0.88; 1.13) | 9.36E-01 | 0.94 (0.78; 1.15) | 5.58E-01 | 0.98 (0.85; 1.13) | 7.52E-01 |
| **Amino acids** |  |  |  |  |  |  |
| Arginine | 1.02 (0.89; 1.15) | 8.10E-01 | 0.91 (0.79; 1.05) | 2.03E-01 | 0.99 (0.85; 1.16) | 9.37E-01 |
| Glutamine | 1.03 (0.90; 1.18) | 6.70E-01 | 1.05 (0.84; 1.31) | 6.54E-01 | 0.94 (0.81; 1.10) | 4.31E-01 |
| Glycine | 0.99 (0.87; 1.14) | 9.20E-01 | 1.21 (0.86; 1.69) | 2.70E-01 | 0.91 (0.80; 1.04) | 1.71E-01 |
| Histidine | 1.03 (0.88; 1.19) | 7.36E-01 | 1.24 (0.98; 1.57) | 7.56E-02 | 0.90 (0.77; 1.05) | 1.69E-01 |
| Methionine | 1.14 (0.98; 1.33) | 9.13E-02 | 1.21 (1.01; 1.45) | 4.23E-02 | 1.01 (0.82; 1.26) | 9.03E-01 |
| Ornithine | 1.06 (0.93; 1.22) | 3.82E-01 | 1.05 (0.89; 1.25) | 5.52E-01 | 0.98 (0.81; 1.18) | 8.07E-01 |
| Phenylalanine | 1.15 (1.00; 1.32) | 4.65E-02 | 1.18 (0.96; 1.45) | 1.18E-01 | 1.06 (0.87; 1.28) | 5.83E-01 |
| Proline | 1.04 (0.93; 1.17) | 4.50E-01 | 1.06 (0.99; 1.14) | 7.89E-02 | 1.01 (0.82; 1.24) | 9.41E-01 |
| Serine | 0.89 (0.78; 1.01) | 7.29E-02 | 0.97 (0.73; 1.28) | 8.11E-01 | 0.86 (0.76; 0.98) | 1.89E-02 |
| Threonine | 0.87 (0.77; 0.98) | 2.70E-02 | 0.83 (0.66; 1.03) | 8.53E-02 | 0.87 (0.76; 1.00) | 4.86E-02 |
| Tryptophan | 1.20 (1.07; 1.35)* | 2.29E-03 | 1.28 (1.05; 1.55) | 1.59E-02 | 1.05 (0.90; 1.23) | 5.06E-01 |
| Tyrosine | 1.33 (1.10; 1.60)* | 3.87E-03 | 1.53 (1.26; 1.86) | 3.97E-05 | 1.16 (0.91; 1.48) | 2.28E-01 |
| Valine | 1.06 (0.90; 1.24) | 4.96E-01 | 1.15 (0.89; 1.47) | 2.78E-01 | 0.96 (0.81; 1.15) | 6.77E-01 |
| (Iso)Leucine | 1.16 (0.98; 1.38) | 8.10E-02 | 1.18 (0.95; 1.45) | 1.26E-01 | 1.07 (0.82; 1.40) | 6.05E-01 |
| **Hexoses** |  |  |  |  |  |  |
| Hexoses | 1.16 (1.00; 1.34) | 5.67E-02 | 1.18 (0.98; 1.42) | 8.01E-02 | 1.14 (0.94; 1.39) | 1.82E-01 |
| **Aggregate measures** |  |  |  |  |  |  |
| (C2 + C3) / C0 | 0.85 (0.71; 1.00) | 5.27E-02 | 0.97 (0.67; 1.41) | 8.84E-01 | 0.84 (0.71; 0.99) | 4.14E-02 |
| Aromatic amino acids (AAA) | 1.29 (1.11; 1.50)* | 1.21E-03 | 1.41 (1.16; 1.72) | 7.52E-04 | 1.13 (0.91; 1.40) | 2.58E-01 |
| C2 / C0 | 0.85 (0.71; 1.00) | 5.38E-02 | 0.97 (0.66; 1.41) | 8.59E-01 | 0.84 (0.71; 0.99) | 4.35E-02 |
| (C16 + C18) / C0 | 0.97 (0.85; 1.12) | 7.09E-01 | 0.97 (0.81; 1.17) | 7.63E-01 | 1.02 (0.84; 1.23) | 8.55E-01 |
| MUFA(PC) | 1.27 (1.13; 1.44)* | 1.06E-04 | 1.20 (0.94; 1.53) | 1.44E-01 | 1.22 (1.05; 1.42) | 9.05E-03 |
| MUFA(PC) / SFA(PC) | 1.19 (1.05; 1.36) | 8.68E-03 | 1.17 (0.99; 1.37) | 5.82E-02 | 1.16 (0.97; 1.38) | 1.01E-01 |
| Ornithine / Arginine | 1.03 (0.91; 1.18) | 6.28E-01 | 1.08 (0.94; 1.23) | 2.63E-01 | 0.97 (0.81; 1.18) | 7.76E-01 |
| PUFA(PC) | 1.24 (1.08; 1.42)* | 2.01E-03 | 1.11 (0.86; 1.43) | 4.36E-01 | 1.19 (1.01; 1.42) | 4.02E-02 |
| PUFA(PC) / MUFA(PC) | 0.89 (0.78; 1.01) | 6.98E-02 | 0.89 (0.74; 1.06) | 1.92E-01 | 0.89 (0.76; 1.04) | 1.42E-01 |
| PUFA(PC) / SFA(PC) | 1.08 (0.95; 1.22) | 2.28E-01 | 1.02 (0.89; 1.17) | 7.68E-01 | 1.10 (0.91; 1.32) | 3.16E-01 |
| SFA(PC) | 1.14 (0.99; 1.32) | 7.54E-02 | 1.04 (0.85; 1.26) | 7.11E-01 | 1.15 (0.95; 1.40) | 1.44E-01 |
| Total PC + Total SM | 1.23 (1.07; 1.40)* | 2.84E-03 | 1.08 (0.84; 1.39) | 5.44E-01 | 1.19 (1.02; 1.40) | 3.25E-02 |
| Total AC / C0 | 0.83 (0.70; 0.98) | 3.03E-02 | 0.94 (0.63; 1.40) | 7.47E-01 | 0.84 (0.71; 0.99) | 4.16E-02 |
| Total AC-DC / Total AC | 1.02 (0.87; 1.20) | 7.66E-01 | 1.09 (0.88; 1.34) | 4.31E-01 | 0.97 (0.80; 1.18) | 7.92E-01 |
| Total AC-OH / Total AC | 1.08 (0.94; 1.24) | 2.82E-01 | 1.12 (0.90; 1.39) | 3.23E-01 | 1.04 (0.88; 1.22) | 6.69E-01 |
| Total lysoPC | 0.97 (0.85; 1.11) | 6.51E-01 | 0.89 (0.75; 1.07) | 2.09E-01 | 0.96 (0.81; 1.14) | 6.43E-01 |
| Total lysoPC / Total PC | 0.81 (0.70; 0.95) | 7.94E-03 | 0.79 (0.60; 1.03) | 8.36E-02 | 0.87 (0.72; 1.04) | 1.24E-01 |
| Total PC | 1.26 (1.10; 1.43)* | 7.36E-04 | 1.14 (0.88; 1.48) | 3.32E-01 | 1.21 (1.03; 1.43) | 2.24E-02 |
| Total diacyl PC | 1.27 (1.12; 1.45)* | 3.21E-04 | 1.17 (0.90; 1.52) | 2.34E-01 | 1.22 (1.04; 1.43) | 1.82E-02 |
| Total acyl-alkyl PC | 1.03 (0.90; 1.18) | 6.60E-01 | 0.83 (0.70; 0.98) | 2.55E-02 | 1.09 (0.92; 1.28) | 3.14E-01 |
| Total SM | 1.01 (0.89; 1.15) | 8.34E-01 | 0.86 (0.72; 1.02) | 8.10E-02 | 1.05 (0.90; 1.21) | 5.59E-01 |
| Total SM / (Total SM + Total PC) | 0.80 (0.71; 0.89)* | 1.42E-04 | 0.76 (0.63; 0.92) | 5.12E-03 | 0.87 (0.74; 1.02) | 8.81E-02 |
| Total SM / Total PC | 0.80 (0.71; 0.89)* | 1.66E-04 | 0.76 (0.63; 0.92) | 5.68E-03 | 0.87 (0.74; 1.03) | 9.48E-02 |
| Total SM-non OH | 1.00 (0.88; 1.13) | 9.98E-01 | 0.84 (0.71; 1.01) | 6.05E-02 | 1.04 (0.91; 1.21) | 5.47E-01 |
| Total SM-OH | 1.08 (0.93; 1.26) | 2.81E-01 | 1.00 (0.81; 1.24) | 9.86E-01 | 1.04 (0.87; 1.25) | 6.71E-01 |
| Total SM-OH / Total SM-non OH | 1.12 (0.96; 1.31) | 1.46E-01 | 1.20 (0.94; 1.53) | 1.51E-01 | 1.01 (0.84; 1.22) | 9.27E-01 |
| Tyrosine / Phenylalanine | 1.30 (1.11; 1.52)* | 1.35E-03 | 1.54 (1.24; 1.92) | 1.93E-04 | 1.15 (0.96; 1.39) | 1.27E-01 |

**Model 3 (adjusted for age, sex, total body fat, waist circumference and fasting serum concentrations of triglycerides, HDL cholesterol and total cholesterol)**

|  | **Visceral adipose tissue** | | | | | |
| --- | --- | --- | --- | --- | --- | --- |
|  | Total |  | Men |  | Women |  |
|  | (N = 174) |  | (N = 84) |  | (N = 90) |  |
|  | Estimate (95% CI) | P-value | Estimate (95% CI) | P-value | Estimate (95% CI) | P-value |
| **Acylcarnitines** |  |  |  |  |  |  |
| C0 | -6.2 (-12.2; -0.2) | 4.20E-02 | -6.1 (-14.9; 2.8) | 1.75E-01 | -4.6 (-11.3; 2.1) | 1.79E-01 |
| C2 | -0.3 (-5.8; 5.1) | 9.08E-01 | -2.6 (-17.6; 12.4) | 7.34E-01 | -2.2 (-6.7; 2.2) | 3.24E-01 |
| C3 | -6.4 (-12.2; -0.7) | 2.92E-02 | -5.9 (-12.5; 0.8) | 8.14E-02 | -3.3 (-11.1; 4.5) | 4.03E-01 |
| C3-DC (C4-OH) | 1.9 (-3.9; 7.7) | 5.12E-01 | 6.2 (-5.2; 17.7) | 2.81E-01 | -0.9 (-5.0; 3.3) | 6.87E-01 |
| C3-OH | 1.5 (-3.9; 7.0) | 5.78E-01 | 3.0 (-4.6; 10.6) | 4.35E-01 | -0.7 (-5.8; 4.4) | 7.89E-01 |
| C3:1 | -3.6 (-9.0; 1.9) | 1.95E-01 | -8.0 (-20.8; 4.8) | 2.15E-01 | 0.3 (-4.5; 5.2) | 8.93E-01 |
| C4 | -4.4 (-8.7; -0.2) | 3.87E-02 | -2.3 (-6.2; 1.5) | 2.35E-01 | -5.1 (-13.6; 3.4) | 2.34E-01 |
| C4:1 | -3.8 (-9.7; 2.1) | 2.09E-01 | -7.6 (-14.0; -1.2) | 2.12E-02 | 2.1 (-4.4; 8.6) | 5.30E-01 |
| C5 | -1.1 (-7.9; 5.7) | 7.47E-01 | -3.8 (-12.8; 5.2) | 4.04E-01 | 6.2 (-2.4; 14.9) | 1.56E-01 |
| C5-DC (C6-OH) | 1.9 (-4.8; 8.6) | 5.77E-01 | -2.1 (-9.1; 4.9) | 5.47E-01 | 3.6 (-3.6; 10.7) | 3.21E-01 |
| C5-M-DC | -10.1 (-16.8; -3.3) | 3.78E-03 | -9.4 (-21.1; 2.2) | 1.11E-01 | -5.9 (-11.6; -0.3) | 3.97E-02 |
| C5-OH (C3-DC-M) | -0.7 (-6.8; 5.4) | 8.27E-01 | 2.9 (-6.7; 12.6) | 5.44E-01 | -1.4 (-6.7; 3.9) | 6.06E-01 |
| C5:1 | -3.9 (-9.2; 1.4) | 1.49E-01 | -4.9 (-11.6; 1.7) | 1.45E-01 | 0.2 (-5.6; 6.1) | 9.34E-01 |
| C5:1-DC | -4.5 (-10.1; 1.0) | 1.11E-01 | -7.7 (-17.5; 2.2) | 1.27E-01 | -3.0 (-7.8; 1.9) | 2.28E-01 |
| C6 (C4:1-DC) | -3.1 (-8.7; 2.4) | 2.61E-01 | -6.7 (-16.3; 2.9) | 1.71E-01 | -1.1 (-6.4; 4.3) | 6.92E-01 |
| C6:1 | -2.8 (-8.7; 3.2) | 3.57E-01 | -2.7 (-10.4; 5.1) | 4.95E-01 | 1.5 (-4.8; 7.8) | 6.34E-01 |
| C7-DC | 1.4 (-4.3; 7.2) | 6.24E-01 | -5.2 (-12.1; 1.6) | 1.33E-01 | 2.8 (-5.0; 10.6) | 4.76E-01 |
| C8 | -4.3 (-10.4; 1.8) | 1.68E-01 | -7.1 (-14.2; 0.0) | 4.86E-02 | 0.5 (-8.9; 10.0) | 9.10E-01 |
| C8:1 | 7.2 (0.5; 13.9) | 3.49E-02 | 6.3 (-6.8; 19.3) | 3.42E-01 | 5.4 (0.3; 10.5) | 3.74E-02 |
| C9 | -2.1 (-9.1; 4.9) | 5.59E-01 | -16.2 (-26.5; -6.0) | 2.23E-03 | 8.0 (2.3; 13.7) | 6.63E-03 |
| C10 | -3.9 (-9.7; 1.9) | 1.85E-01 | -7.0 (-13.4; -0.7) | 3.12E-02 | 0.6 (-8.2; 9.4) | 8.93E-01 |
| C10:1 | -4.3 (-11.0; 2.3) | 1.98E-01 | -10.1 (-17.9; -2.3) | 1.20E-02 | 3.6 (-4.1; 11.2) | 3.54E-01 |
| C10:2 | 1.7 (-5.4; 8.8) | 6.29E-01 | -5.0 (-14.2; 4.2) | 2.86E-01 | 6.1 (-0.2; 12.4) | 5.95E-02 |
| C12 | -4.1 (-9.9; 1.7) | 1.66E-01 | -10.3 (-18.3; -2.3) | 1.19E-02 | 1.0 (-4.6; 6.5) | 7.33E-01 |
| C12-DC | -2.1 (-7.8; 3.7) | 4.75E-01 | -8.5 (-22.7; 5.6) | 2.34E-01 | 0.0 (-4.2; 4.2) | 9.92E-01 |
| C12:1 | -2.3 (-7.6; 3.1) | 3.98E-01 | -9.9 (-21.1; 1.2) | 8.03E-02 | -0.8 (-5.0; 3.5) | 7.19E-01 |
| C14 | -4.2 (-11.4; 3.0) | 2.51E-01 | -13.7 (-24.4; -3.0) | 1.26E-02 | 6.1 (-1.7; 13.8) | 1.25E-01 |
| C14:1 | -1.4 (-6.3; 3.4) | 5.63E-01 | -7.9 (-17.2; 1.4) | 9.46E-02 | 1.1 (-4.2; 6.4) | 6.73E-01 |
| C14:1-OH | -2.7 (-9.3; 3.9) | 4.20E-01 | -12.4 (-24.2; -0.5) | 4.08E-02 | 1.2 (-4.6; 7.1) | 6.75E-01 |
| C14:2 | -5.7 (-11.5; 0.1) | 5.42E-02 | -10.6 (-20.1; -1.0) | 3.09E-02 | -1.3 (-6.0; 3.5) | 5.99E-01 |
| C14:2-OH | -3.0 (-8.7; 2.7) | 3.01E-01 | -8.0 (-16.6; 0.7) | 7.06E-02 | 4.1 (-1.6; 9.8) | 1.59E-01 |
| C16 | -1.8 (-8.6; 5.0) | 6.04E-01 | -6.7 (-17.3; 4.0) | 2.15E-01 | 2.6 (-4.4; 9.6) | 4.63E-01 |
| C16-OH | 4.4 (-0.7; 9.5) | 9.04E-02 | 5.1 (-3.5; 13.8) | 2.43E-01 | 4.0 (-1.6; 9.5) | 1.63E-01 |
| C16:1 | 0.1 (-4.9; 5.2) | 9.67E-01 | -4.8 (-13.3; 3.6) | 2.57E-01 | 1.0 (-4.3; 6.2) | 7.19E-01 |
| C16:1-OH | 2.6 (-4.2; 9.3) | 4.51E-01 | -5.5 (-17.4; 6.5) | 3.63E-01 | 5.7 (0.6; 10.8) | 2.85E-02 |
| C16:2 | -2.8 (-9.1; 3.5) | 3.85E-01 | -8.4 (-18.7; 2.0) | 1.11E-01 | 1.8 (-4.3; 7.9) | 5.64E-01 |
| C16:2-OH | -1.8 (-8.8; 5.1) | 6.04E-01 | -14.7 (-26.9; -2.6) | 1.82E-02 | 3.9 (-1.4; 9.2) | 1.48E-01 |
| C18 | -5.8 (-13.8; 2.2) | 1.54E-01 | -11.1 (-24.4; 2.2) | 1.00E-01 | 1.2 (-6.1; 8.6) | 7.39E-01 |
| C18:1 | 2.5 (-2.5; 7.5) | 3.20E-01 | 2.1 (-5.4; 9.5) | 5.85E-01 | 1.3 (-4.1; 6.8) | 6.29E-01 |
| C18:1-OH | 5.3 (-0.1; 10.8) | 5.63E-02 | 1.0 (-16.8; 18.9) | 9.09E-01 | 3.5 (-0.7; 7.6) | 9.95E-02 |
| C18:2 | -2.8 (-8.9; 3.2) | 3.57E-01 | -9.6 (-18.6; -0.6) | 3.76E-02 | 1.4 (-5.4; 8.2) | 6.82E-01 |
| **Lysophosphatidylcholines** |  |  |  |  |  |  |
| Lyso PC a C6:0 | 1.9 (-3.8; 7.5) | 5.16E-01 | -1.1 (-11.7; 9.4) | 8.34E-01 | 0.7 (-4.1; 5.5) | 7.80E-01 |
| Lyso PC a C14:0 | -2.3 (-8.6; 3.9) | 4.62E-01 | -1.9 (-11.4; 7.6) | 6.90E-01 | 0.4 (-6.0; 6.8) | 9.04E-01 |
| Lyso PC a C16:0 | -7.9 (-15.0; -0.7) | 3.07E-02 | -11.3 (-23.3; 0.6) | 6.31E-02 | -4.3 (-10.2; 1.6) | 1.48E-01 |
| Lyso PC a C16:1 | -2.0 (-7.6; 3.6) | 4.81E-01 | -1.4 (-10.6; 7.8) | 7.58E-01 | -2.9 (-8.6; 2.7) | 3.07E-01 |
| Lyso PC a C17:0 | -10.6 (-18.6; -2.5) | 1.01E-02 | -14.3 (-26.4; -2.2) | 2.12E-02 | -6.2 (-12.0; -0.3) | 4.04E-02 |
| Lyso PC a C18:0 | -8.8 (-16.7; -0.9) | 2.90E-02 | -20.1 (-35.3; -5.0) | 9.90E-03 | -1.4 (-6.6; 3.7) | 5.79E-01 |
| Lyso PC a C18:1 | -6.1 (-12.7; 0.5) | 6.92E-02 | -10.8 (-26.7; 5.1) | 1.79E-01 | -3.1 (-8.8; 2.6) | 2.88E-01 |
| Lyso PC a C18:2 | -11.6 (-19.6; -3.5) | 5.01E-03 | -18.3 (-31.1; -5.5) | 5.57E-03 | -2.1 (-9.5; 5.3) | 5.82E-01 |
| Lyso PC a C20:3 | -6.3 (-13.1; 0.4) | 6.54E-02 | -7.6 (-16.8; 1.7) | 1.08E-01 | -1.1 (-7.9; 5.7) | 7.48E-01 |
| Lyso PC a C20:4 | -1.6 (-7.3; 4.1) | 5.77E-01 | -0.2 (-8.4; 8.1) | 9.71E-01 | 0.9 (-5.1; 7.0) | 7.61E-01 |
| Lyso PC a C24:0 | -0.6 (-6.8; 5.6) | 8.47E-01 | -9.5 (-20.3; 1.4) | 8.68E-02 | 2.9 (-2.0; 7.7) | 2.45E-01 |
| Lyso PC a C26:0 | -2.8 (-8.3; 2.7) | 3.15E-01 | -10.7 (-22.0; 0.6) | 6.22E-02 | 0.3 (-5.3; 5.8) | 9.18E-01 |
| Lyso PC a C26:1 | -2.0 (-7.5; 3.6) | 4.83E-01 | -8.4 (-19.8; 3.0) | 1.45E-01 | 0.6 (-4.6; 5.8) | 8.22E-01 |
| Lyso PC a C28:0 | -2.3 (-8.1; 3.5) | 4.33E-01 | -13.7 (-25.7; -1.8) | 2.51E-02 | 2.4 (-2.9; 7.7) | 3.68E-01 |
| Lyso PC a C28:1 | -3.8 (-9.3; 1.8) | 1.83E-01 | -16.9 (-29.8; -4.1) | 1.05E-02 | 0.9 (-4.5; 6.3) | 7.34E-01 |
| **Diacyl phosphatidylcholines** |  |  |  |  |  |  |
| PC aa C24:0 | -0.4 (-5.7; 4.9) | 8.76E-01 | -10.3 (-20.7; 0.0) | 5.09E-02 | 3.0 (-1.5; 7.4) | 1.90E-01 |
| PC aa C26:0 | -1.9 (-7.4; 3.6) | 4.97E-01 | -7.9 (-18.6; 2.8) | 1.47E-01 | 0.1 (-5.5; 5.7) | 9.70E-01 |
| PC aa C28:1 | -0.9 (-8.6; 6.7) | 8.10E-01 | -4.8 (-19.5; 10.0) | 5.23E-01 | 4.1 (-3.1; 11.3) | 2.60E-01 |
| PC aa C30:0 | -4.3 (-12.1; 3.4) | 2.74E-01 | -12.0 (-26.2; 2.3) | 9.83E-02 | 2.1 (-4.6; 8.8) | 5.41E-01 |
| PC aa C30:2 | -6.6 (-12.3; -0.9) | 2.33E-02 | -11.8 (-22.1; -1.5) | 2.58E-02 | -3.3 (-8.3; 1.7) | 1.97E-01 |
| PC aa C32:0 | -1.1 (-8.2; 6.1) | 7.71E-01 | -4.9 (-14.6; 4.7) | 3.13E-01 | 2.4 (-5.7; 10.5) | 5.54E-01 |
| PC aa C32:1 | 1.9 (-5.8; 9.7) | 6.26E-01 | 5.2 (-6.9; 17.4) | 3.94E-01 | 0.6 (-6.3; 7.6) | 8.54E-01 |
| PC aa C32:2 | -1.0 (-7.0; 5.0) | 7.50E-01 | -7.3 (-18.1; 3.5) | 1.83E-01 | 4.5 (-0.6; 9.6) | 8.33E-02 |
| PC aa C32:3 | -5.7 (-14.7; 3.3) | 2.11E-01 | -16.9 (-31.8; -1.9) | 2.75E-02 | 4.2 (-1.4; 9.8) | 1.44E-01 |
| PC aa C34:1 | 4.6 (-4.8; 14.1) | 3.36E-01 | 13.5 (-1.8; 28.8) | 8.39E-02 | 2.7 (-6.9; 12.4) | 5.73E-01 |
| PC aa C34:2 | 0.4 (-7.4; 8.2) | 9.11E-01 | -6.6 (-18.2; 5.1) | 2.64E-01 | 5.7 (-2.4; 13.7) | 1.67E-01 |
| PC aa C34:3 | 2.6 (-6.2; 11.3) | 5.65E-01 | 1.2 (-13.2; 15.5) | 8.70E-01 | 6.9 (-1.0; 14.9) | 8.73E-02 |
| PC aa C34:4 | 5.1 (-1.5; 11.6) | 1.28E-01 | 5.4 (-4.9; 15.6) | 3.00E-01 | 7.9 (2.8; 13.1) | 2.96E-03 |
| PC aa C36:0 | -1.2 (-9.1; 6.7) | 7.72E-01 | -6.3 (-15.5; 3.0) | 1.81E-01 | 8.5 (1.8; 15.1) | 1.35E-02 |
| PC aa C36:1 | -1.8 (-11.2; 7.6) | 7.04E-01 | -6.8 (-22.3; 8.6) | 3.80E-01 | 4.5 (-3.5; 12.5) | 2.65E-01 |
| PC aa C36:2 | -3.0 (-12.1; 6.1) | 5.15E-01 | -13.0 (-26.5; 0.5) | 5.80E-02 | 6.5 (-1.4; 14.3) | 1.08E-01 |
| PC aa C36:3 | -0.8 (-8.9; 7.2) | 8.43E-01 | -3.2 (-14.3; 7.9) | 5.64E-01 | 4.8 (-4.5; 14.0) | 3.08E-01 |
| PC aa C36:4 | 8.2 (1.5; 14.9) | 1.71E-02 | 11.8 (0.5; 23.2) | 4.13E-02 | 7.8 (0.4; 15.1) | 3.87E-02 |
| PC aa C36:5 | 4.1 (-2.1; 10.2) | 1.93E-01 | 0.2 (-8.5; 8.8) | 9.70E-01 | 6.6 (1.0; 12.1) | 2.04E-02 |
| PC aa C36:6 | 1.9 (-5.0; 8.9) | 5.84E-01 | -5.0 (-17.8; 7.7) | 4.33E-01 | 6.7 (1.7; 11.7) | 9.06E-03 |
| PC aa C38:0 | -2.0 (-9.7; 5.6) | 6.04E-01 | -8.4 (-18.1; 1.3) | 9.01E-02 | 5.7 (-0.8; 12.2) | 8.72E-02 |
| PC aa C38:1 | -1.2 (-7.3; 4.9) | 6.99E-01 | -3.6 (-12.6; 5.5) | 4.34E-01 | 2.2 (-2.7; 7.0) | 3.73E-01 |
| PC aa C38:3 | -1.5 (-11.0; 7.9) | 7.47E-01 | -5.8 (-18.4; 6.7) | 3.58E-01 | 5.1 (-3.3; 13.5) | 2.30E-01 |
| PC aa C38:4 | 7.0 (-0.8; 14.8) | 7.67E-02 | 8.8 (-3.7; 21.4) | 1.67E-01 | 8.9 (2.6; 15.1) | 5.90E-03 |
| PC aa C38:5 | 6.6 (-0.2; 13.3) | 5.66E-02 | 4.9 (-5.5; 15.2) | 3.50E-01 | 8.9 (1.6; 16.1) | 1.77E-02 |
| PC aa C38:6 | 2.9 (-4.1; 9.9) | 4.09E-01 | -3.1 (-13.5; 7.3) | 5.58E-01 | 7.2 (1.5; 12.8) | 1.32E-02 |
| PC aa C40:1 | -6.8 (-14.0; 0.4) | 6.51E-02 | -14.2 (-23.7; -4.7) | 3.84E-03 | 2.5 (-3.6; 8.7) | 4.14E-01 |
| PC aa C40:2 | -4.5 (-11.9; 3.0) | 2.37E-01 | -12.8 (-23.7; -1.9) | 2.19E-02 | 4.1 (-1.2; 9.4) | 1.31E-01 |
| PC aa C40:3 | -6.4 (-14.5; 1.8) | 1.24E-01 | -13.5 (-24.0; -3.0) | 1.25E-02 | 1.7 (-6.0; 9.4) | 6.66E-01 |
| PC aa C40:4 | 1.3 (-6.1; 8.8) | 7.23E-01 | 1.4 (-9.8; 12.6) | 8.04E-01 | 3.4 (-3.4; 10.2) | 3.17E-01 |
| PC aa C40:5 | 2.4 (-5.4; 10.1) | 5.48E-01 | -1.8 (-13.4; 9.8) | 7.59E-01 | 6.5 (-0.6; 13.5) | 7.24E-02 |
| PC aa C40:6 | 1.3 (-7.0; 9.6) | 7.54E-01 | -6.7 (-18.2; 4.9) | 2.55E-01 | 8.8 (2.6; 15.1) | 5.81E-03 |
| PC aa C42:0 | -6.2 (-12.8; 0.5) | 7.02E-02 | -12.2 (-23.4; -1.0) | 3.36E-02 | -0.6 (-5.8; 4.6) | 8.14E-01 |
| PC aa C42:1 | -4.1 (-9.8; 1.7) | 1.68E-01 | -8.3 (-19.7; 3.1) | 1.49E-01 | -1.4 (-6.6; 3.8) | 5.89E-01 |
| PC aa C42:2 | -4.0 (-10.9; 2.9) | 2.49E-01 | -6.9 (-16.2; 2.4) | 1.45E-01 | 1.9 (-4.4; 8.1) | 5.53E-01 |
| PC aa C42:4 | -3.2 (-10.4; 4.1) | 3.93E-01 | -9.7 (-26.2; 6.7) | 2.43E-01 | 0.3 (-4.6; 5.2) | 8.90E-01 |
| PC aa C42:5 | 0.7 (-6.7; 8.0) | 8.60E-01 | -4.7 (-13.9; 4.6) | 3.20E-01 | 4.7 (-2.0; 11.3) | 1.65E-01 |
| PC aa C42:6 | -1.7 (-8.5; 5.1) | 6.27E-01 | -8.9 (-18.1; 0.4) | 5.93E-02 | 0.9 (-5.1; 7.0) | 7.62E-01 |
| **Acyl-alkyl phosphatidylcholines** |  |  |  |  |  |  |
| PC ae C30:0 | -8.3 (-16.4; -0.2) | 4.43E-02 | -23.0 (-35.9; -10.2) | 6.06E-04 | 2.8 (-3.2; 8.8) | 3.59E-01 |
| PC ae C30:1 | -5.3 (-12.6; 2.1) | 1.57E-01 | -17.0 (-31.4; -2.6) | 2.13E-02 | 1.0 (-4.3; 6.2) | 7.09E-01 |
| PC ae C30:2 | -2.8 (-9.3; 3.8) | 4.09E-01 | -13.6 (-25.6; -1.6) | 2.64E-02 | 4.9 (-1.9; 11.7) | 1.56E-01 |
| PC ae C32:1 | -6.4 (-14.3; 1.6) | 1.17E-01 | -18.6 (-31.6; -5.6) | 5.67E-03 | 3.3 (-4.1; 10.6) | 3.79E-01 |
| PC ae C32:2 | -6.5 (-14.7; 1.7) | 1.19E-01 | -22.4 (-38.2; -6.7) | 5.85E-03 | 3.9 (-3.6; 11.4) | 3.03E-01 |
| PC ae C34:0 | -10.0 (-18.7; -1.3) | 2.41E-02 | -18.6 (-32.4; -4.7) | 9.28E-03 | -1.2 (-9.0; 6.6) | 7.58E-01 |
| PC ae C34:1 | -7.6 (-16.0; 0.8) | 7.51E-02 | -15.9 (-30.5; -1.3) | 3.32E-02 | -0.1 (-7.8; 7.6) | 9.75E-01 |
| PC ae C34:2 | -8.6 (-19.0; 1.9) | 1.07E-01 | -22.7 (-37.9; -7.5) | 3.97E-03 | 3.4 (-5.5; 12.4) | 4.49E-01 |
| PC ae C34:3 | -5.1 (-13.6; 3.3) | 2.34E-01 | -19.2 (-41.9; 3.5) | 9.68E-02 | 1.2 (-3.6; 6.0) | 6.15E-01 |
| PC ae C36:0 | -0.4 (-10.1; 9.2) | 9.28E-01 | -1.4 (-12.6; 9.8) | 8.08E-01 | 2.5 (-7.6; 12.6) | 6.25E-01 |
| PC ae C36:1 | -3.4 (-11.6; 4.9) | 4.24E-01 | -5.8 (-20.8; 9.3) | 4.47E-01 | 0.9 (-6.8; 8.7) | 8.12E-01 |
| PC ae C36:2 | -9.0 (-17.6; -0.3) | 4.18E-02 | -15.7 (-28.5; -2.9) | 1.71E-02 | -1.5 (-8.6; 5.6) | 6.76E-01 |
| PC ae C36:3 | -8.9 (-18.4; 0.6) | 6.52E-02 | -20.4 (-34.8; -5.9) | 6.19E-03 | 1.8 (-6.5; 10.0) | 6.71E-01 |
| PC ae C36:4 | -0.7 (-7.7; 6.2) | 8.33E-01 | -4.1 (-14.3; 6.0) | 4.21E-01 | 5.1 (-1.7; 12.0) | 1.41E-01 |
| PC ae C36:5 | 2.3 (-4.8; 9.4) | 5.28E-01 | -0.4 (-12.9; 12.1) | 9.47E-01 | 6.2 (0.6; 11.7) | 2.93E-02 |
| PC ae C38:0 | 1.4 (-5.8; 8.7) | 7.00E-01 | -7.9 (-21.6; 5.8) | 2.52E-01 | 6.9 (1.9; 11.9) | 7.61E-03 |
| PC ae C38:1 | -2.9 (-9.2; 3.4) | 3.59E-01 | -9.5 (-20.6; 1.6) | 9.27E-02 | 1.4 (-4.8; 7.6) | 6.55E-01 |
| PC ae C38:2 | -12.0 (-20.1; -3.8) | 4.26E-03 | -22.2 (-36.8; -7.6) | 3.31E-03 | -2.6 (-9.4; 4.2) | 4.43E-01 |
| PC ae C38:3 | -7.7 (-16.4; 1.0) | 8.31E-02 | -13.1 (-25.6; -0.7) | 3.92E-02 | -1.6 (-8.5; 5.2) | 6.37E-01 |
| PC ae C38:4 | -1.7 (-8.9; 5.5) | 6.36E-01 | -2.6 (-13.5; 8.3) | 6.38E-01 | 1.2 (-5.0; 7.5) | 7.01E-01 |
| PC ae C38:5 | 1.0 (-5.5; 7.5) | 7.59E-01 | -0.8 (-10.9; 9.3) | 8.78E-01 | 4.2 (-2.0; 10.3) | 1.83E-01 |
| PC ae C38:6 | 0.3 (-7.6; 8.2) | 9.35E-01 | -5.3 (-15.0; 4.4) | 2.79E-01 | 9.3 (2.6; 16.0) | 7.32E-03 |
| PC ae C40:0 | 0.8 (-5.8; 7.5) | 8.02E-01 | -2.4 (-16.5; 11.6) | 7.30E-01 | 1.2 (-5.0; 7.4) | 6.96E-01 |
| PC ae C40:1 | -1.3 (-10.2; 7.6) | 7.69E-01 | -13.6 (-31.9; 4.7) | 1.43E-01 | 6.5 (1.2; 11.7) | 1.65E-02 |
| PC ae C40:2 | -2.9 (-10.7; 5.0) | 4.75E-01 | -10.1 (-25.3; 5.0) | 1.88E-01 | 1.9 (-4.9; 8.7) | 5.76E-01 |
| PC ae C40:3 | -10.0 (-18.6; -1.4) | 2.27E-02 | -23.5 (-39.3; -7.6) | 4.31E-03 | -2.2 (-9.7; 5.2) | 5.54E-01 |
| PC ae C40:4 | -6.4 (-12.8; 0.0) | 5.04E-02 | -11.3 (-21.9; -0.7) | 3.76E-02 | -1.7 (-6.9; 3.6) | 5.36E-01 |
| PC ae C40:5 | -3.5 (-9.9; 2.9) | 2.86E-01 | -6.9 (-18.4; 4.7) | 2.41E-01 | 0.1 (-5.5; 5.7) | 9.74E-01 |
| PC ae C40:6 | -3.4 (-11.1; 4.3) | 3.81E-01 | -11.1 (-23.4; 1.2) | 7.56E-02 | 2.4 (-3.5; 8.4) | 4.19E-01 |
| PC ae C42:0 | -2.1 (-8.5; 4.3) | 5.12E-01 | -7.1 (-17.2; 3.0) | 1.66E-01 | -0.7 (-7.0; 5.5) | 8.15E-01 |
| PC ae C42:1 | -3.7 (-10.0; 2.6) | 2.46E-01 | -11.8 (-26.1; 2.4) | 1.02E-01 | 1.4 (-3.6; 6.5) | 5.75E-01 |
| PC ae C42:2 | -10.7 (-20.0; -1.4) | 2.50E-02 | -20.8 (-35.9; -5.7) | 7.49E-03 | -0.9 (-7.9; 6.0) | 7.88E-01 |
| PC ae C42:3 | -5.7 (-14.1; 2.6) | 1.78E-01 | -12.9 (-26.6; 0.8) | 6.43E-02 | 1.8 (-4.5; 8.1) | 5.76E-01 |
| PC ae C42:4 | -9.9 (-16.8; -3.0) | 5.23E-03 | -15.7 (-26.3; -5.2) | 4.02E-03 | -3.5 (-9.8; 2.9) | 2.78E-01 |
| PC ae C42:5 | -5.8 (-11.9; 0.2) | 5.94E-02 | -9.2 (-20.9; 2.4) | 1.18E-01 | -1.9 (-6.8; 3.0) | 4.44E-01 |
| PC ae C44:3 | -4.4 (-12.2; 3.4) | 2.65E-01 | -15.7 (-29.6; -1.8) | 2.76E-02 | 2.8 (-2.9; 8.6) | 3.28E-01 |
| PC ae C44:4 | -6.7 (-12.8; -0.5) | 3.30E-02 | -12.4 (-20.9; -4.0) | 4.56E-03 | -2.0 (-7.2; 3.2) | 4.46E-01 |
| PC ae C44:5 | -3.0 (-9.1; 3.2) | 3.41E-01 | -3.2 (-12.2; 5.8) | 4.76E-01 | 0.0 (-5.2; 5.3) | 9.87E-01 |
| PC ae C44:6 | -6.8 (-13.3; -0.4) | 3.76E-02 | -10.9 (-20.8; -0.9) | 3.33E-02 | -2.2 (-7.2; 2.8) | 3.82E-01 |
| **Sphingomyelins** |  |  |  |  |  |  |
| SM (OH) C14:1 | -8.0 (-16.3; 0.4) | 6.17E-02 | -14.5 (-27.3; -1.8) | 2.63E-02 | 0.7 (-6.4; 7.9) | 8.38E-01 |
| SM (OH) C16:1 | -8.3 (-17.5; 0.8) | 7.47E-02 | -20.5 (-34.4; -6.6) | 4.28E-03 | 1.2 (-5.4; 7.8) | 7.22E-01 |
| SM (OH) C22:1 | -5.2 (-15.2; 4.8) | 3.06E-01 | -10.0 (-24.9; 4.9) | 1.86E-01 | 2.5 (-5.6; 10.6) | 5.39E-01 |
| SM (OH) C22:2 | -8.1 (-18.1; 1.8) | 1.10E-01 | -14.1 (-31.0; 2.8) | 9.96E-02 | -1.2 (-10.2; 7.9) | 7.98E-01 |
| SM (OH) C24:1 | -4.5 (-12.1; 3.1) | 2.46E-01 | -7.2 (-24.0; 9.5) | 3.92E-01 | -0.7 (-6.6; 5.2) | 8.13E-01 |
| SM C16:0 | -13.4 (-23.6; -3.3) | 9.71E-03 | -25.4 (-41.5; -9.2) | 2.42E-03 | -1.9 (-10.3; 6.5) | 6.59E-01 |
| SM C16:1 | -5.9 (-15.1; 3.3) | 2.04E-01 | -10.8 (-27.3; 5.7) | 1.96E-01 | 0.3 (-8.8; 9.3) | 9.53E-01 |
| SM C18:0 | -3.9 (-12.1; 4.4) | 3.59E-01 | -14.5 (-29.8; 0.8) | 6.33E-02 | 3.0 (-3.2; 9.3) | 3.39E-01 |
| SM C18:1 | -4.9 (-13.3; 3.6) | 2.56E-01 | -14.4 (-29.2; 0.4) | 5.57E-02 | 2.2 (-4.5; 8.9) | 5.19E-01 |
| SM C20:2 | -2.3 (-10.4; 5.8) | 5.74E-01 | -11.8 (-31.5; 7.9) | 2.38E-01 | 0.7 (-5.3; 6.7) | 8.23E-01 |
| SM C22:3 | -4.1 (-10.4; 2.3) | 2.06E-01 | -12.3 (-26.6; 2.1) | 9.41E-02 | -2.0 (-6.5; 2.5) | 3.70E-01 |
| SM C24:0 | -3.9 (-12.3; 4.5) | 3.59E-01 | -4.0 (-15.5; 7.5) | 4.94E-01 | -0.8 (-7.7; 6.1) | 8.11E-01 |
| SM C24:1 | -3.3 (-10.4; 3.8) | 3.63E-01 | -3.3 (-15.1; 8.6) | 5.84E-01 | -2.3 (-9.2; 4.6) | 5.08E-01 |
| SM C26:0 | -0.5 (-6.7; 5.7) | 8.76E-01 | 0.1 (-16.1; 16.4) | 9.86E-01 | -0.4 (-6.6; 5.7) | 8.85E-01 |
| SM C26:1 | -6.2 (-13.1; 0.7) | 7.96E-02 | -11.1 (-22.4; 0.1) | 5.20E-02 | 0.5 (-5.4; 6.3) | 8.74E-01 |
| **Amino acids** |  |  |  |  |  |  |
| Arginine | -3.9 (-12.1; 4.3) | 3.48E-01 | -5.0 (-17.4; 7.3) | 4.20E-01 | -2.7 (-9.1; 3.8) | 4.13E-01 |
| Glutamine | -3.9 (-11.5; 3.6) | 3.04E-01 | -11.5 (-26.6; 3.5) | 1.32E-01 | -1.4 (-7.3; 4.5) | 6.45E-01 |
| Glycine | -6.0 (-12.7; 0.7) | 7.82E-02 | -20.5 (-40.5; -0.5) | 4.51E-02 | -4.3 (-9.1; 0.5) | 7.56E-02 |
| Histidine | 1.3 (-4.6; 7.1) | 6.70E-01 | -5.7 (-18.3; 7.0) | 3.77E-01 | 2.0 (-2.1; 6.2) | 3.25E-01 |
| Methionine | -0.3 (-6.1; 5.5) | 9.14E-01 | -3.0 (-10.3; 4.3) | 4.13E-01 | 3.9 (-1.5; 9.3) | 1.53E-01 |
| Ornithine | -3.7 (-11.2; 3.9) | 3.42E-01 | -4.9 (-18.8; 8.9) | 4.80E-01 | -2.5 (-7.4; 2.5) | 3.26E-01 |
| Phenylalanine | 5.7 (0.9; 10.5) | 1.95E-02 | 4.7 (-3.0; 12.3) | 2.27E-01 | 6.2 (1.0; 11.4) | 1.93E-02 |
| Proline | -5.7 (-10.0; -1.5) | 8.39E-03 | -4.2 (-10.3; 1.8) | 1.66E-01 | -2.5 (-10.7; 5.8) | 5.55E-01 |
| Serine | -2.9 (-9.5; 3.8) | 3.97E-01 | -2.5 (-17.7; 12.8) | 7.48E-01 | -1.8 (-6.6; 3.1) | 4.70E-01 |
| Threonine | -2.6 (-8.8; 3.6) | 4.07E-01 | -6.6 (-19.4; 6.2) | 3.06E-01 | -0.3 (-5.6; 5.0) | 9.16E-01 |
| Tryptophan | 2.6 (-3.2; 8.4) | 3.80E-01 | 2.2 (-7.2; 11.6) | 6.39E-01 | 3.6 (-1.9; 9.1) | 1.99E-01 |
| Tyrosine | 5.2 (0.0; 10.5) | 5.18E-02 | 3.4 (-4.4; 11.1) | 3.89E-01 | 6.0 (1.3; 10.7) | 1.36E-02 |
| Valine | 0.9 (-5.6; 7.5) | 7.80E-01 | -1.0 (-12.1; 10.1) | 8.56E-01 | 4.4 (-1.6; 10.4) | 1.48E-01 |
| (Iso)Leucine | 1.4 (-6.7; 9.6) | 7.28E-01 | -0.9 (-11.2; 9.5) | 8.65E-01 | 8.4 (0.7; 16.1) | 3.21E-02 |
| **Hexoses** |  |  |  |  |  |  |
| Hexoses | 5.4 (-0.6; 11.4) | 7.69E-02 | 3.2 (-4.3; 10.8) | 3.98E-01 | 9.6 (4.7; 14.5) | 1.85E-04 |
| **Aggregate measures** |  |  |  |  |  |  |
| (C2 + C3) / C0 | 3.5 (-2.3; 9.2) | 2.36E-01 | 3.7 (-8.5; 15.9) | 5.50E-01 | -0.2 (-4.9; 4.5) | 9.43E-01 |
| Aromatic amino acids (AAA) | 5.1 (-0.3; 10.4) | 6.56E-02 | 3.7 (-4.4; 11.8) | 3.68E-01 | 6.3 (1.2; 11.3) | 1.51E-02 |
| C2 / C0 | 3.5 (-2.2; 9.3) | 2.28E-01 | 3.9 (-8.3; 16.1) | 5.25E-01 | -0.2 (-4.9; 4.5) | 9.37E-01 |
| (C16 + C18) / C0 | 2.0 (-3.4; 7.5) | 4.63E-01 | -3.7 (-13.8; 6.5) | 4.72E-01 | 4.2 (-2.1; 10.6) | 1.91E-01 |
| MUFA(PC) | 2.8 (-6.3; 11.8) | 5.49E-01 | 7.8 (-6.8; 22.3) | 2.91E-01 | 3.5 (-6.4; 13.4) | 4.84E-01 |
| MUFA(PC) / SFA(PC) | 5.9 (-1.6; 13.5) | 1.23E-01 | 20.5 (6.3; 34.7) | 5.16E-03 | -1.3 (-6.7; 4.1) | 6.39E-01 |
| Ornithine / Arginine | 0.0 (-7.9; 8.0) | 9.94E-01 | -0.6 (-13.7; 12.5) | 9.25E-01 | 0.4 (-5.4; 6.1) | 9.04E-01 |
| PUFA(PC) | 3.9 (-5.5; 13.3) | 4.16E-01 | -4.1 (-16.7; 8.4) | 5.15E-01 | 12.3 (4.5; 20.2) | 2.49E-03 |
| PUFA(PC) / MUFA(PC) | 0.6 (-6.6; 7.8) | 8.69E-01 | -9.4 (-22.6; 3.7) | 1.58E-01 | 5.1 (-0.6; 10.9) | 7.94E-02 |
| PUFA(PC) / SFA(PC) | 5.5 (-0.2; 11.2) | 5.72E-02 | 6.8 (-2.4; 16.0) | 1.44E-01 | 4.7 (-1.7; 11.1) | 1.46E-01 |
| SFA(PC) | -3.7 (-13.4; 5.9) | 4.43E-01 | -11.7 (-24.9; 1.4) | 7.99E-02 | 6.6 (-2.4; 15.7) | 1.50E-01 |
| Total PC + Total SM | 1.1 (-8.9; 11.1) | 8.29E-01 | -6.7 (-19.5; 6.1) | 3.00E-01 | 10.2 (1.3; 19.1) | 2.45E-02 |
| Total AC / C0 | 3.3 (-2.1; 8.8) | 2.26E-01 | 0.7 (-10.9; 12.2) | 9.07E-01 | 0.5 (-4.5; 5.5) | 8.52E-01 |
| Total AC-DC / Total AC | -0.2 (-6.3; 5.8) | 9.38E-01 | 3.1 (-10.5; 16.6) | 6.53E-01 | -0.1 (-6.2; 5.9) | 9.68E-01 |
| Total AC-OH / Total AC | 3.6 (-2.3; 9.5) | 2.26E-01 | 11.5 (-3.4; 26.5) | 1.29E-01 | 2.4 (-2.6; 7.3) | 3.48E-01 |
| Total lysoPC | -9.4 (-16.8; -2.0) | 1.30E-02 | -16.1 (-29.3; -2.9) | 1.74E-02 | -3.0 (-9.1; 3.0) | 3.19E-01 |
| Total lysoPC / Total PC | -14.1 (-21.7; -6.6)* | 3.05E-04 | -20.4 (-37.2; -3.5) | 1.82E-02 | -8.0 (-13.1; -2.9) | 2.37E-03 |
| Total PC | 4.0 (-5.5; 13.4) | 4.12E-01 | -2.5 (-14.5; 9.5) | 6.80E-01 | 11.8 (3.1; 20.6) | 8.82E-03 |
| Total diacyl PC | 5.2 (-3.9; 14.4) | 2.60E-01 | -0.2 (-12.1; 11.7) | 9.71E-01 | 12.3 (3.5; 21.1) | 6.50E-03 |
| Total acyl-alkyl PC | -5.9 (-15.8; 4.0) | 2.43E-01 | -14.6 (-29.3; 0.1) | 5.20E-02 | 4.2 (-4.1; 12.5) | 3.18E-01 |
| Total SM | -9.6 (-19.7; 0.4) | 5.96E-02 | -19.4 (-35.6; -3.3) | 1.88E-02 | -0.8 (-9.3; 7.8) | 8.60E-01 |
| Total SM / (Total SM + Total PC) | -13.5 (-20.3; -6.8)* | 1.05E-04 | -19.0 (-31.4; -6.6) | 3.03E-03 | -9.4 (-15.3; -3.5) | 2.10E-03 |
| Total SM / Total PC | -13.5 (-20.1; -6.8)* | 1.02E-04 | -19.0 (-31.4; -6.7) | 2.87E-03 | -9.3 (-15.2; -3.5) | 2.16E-03 |
| Total SM-non OH | -9.1 (-18.6; 0.3) | 5.73E-02 | -18.3 (-33.6; -3.1) | 1.92E-02 | -1.0 (-9.1; 7.1) | 8.06E-01 |
| Total SM-OH | -8.5 (-19.1; 2.2) | 1.19E-01 | -16.8 (-34.1; 0.5) | 5.66E-02 | 0.9 (-7.8; 9.7) | 8.31E-01 |
| Total SM-OH / Total SM-non OH | -0.6 (-7.0; 5.8) | 8.56E-01 | 0.1 (-9.6; 9.8) | 9.86E-01 | 2.5 (-1.9; 6.9) | 2.54E-01 |
| Tyrosine / Phenylalanine | 1.6 (-3.8; 7.1) | 5.58E-01 | -1.7 (-12.2; 8.8) | 7.53E-01 | 2.3 (-2.8; 7.4) | 3.68E-01 |
|  | **Hepatic triglyceride content** | | | | | |
|  | Total |  | Men |  | Women |  |
|  | (N = 174) |  | (N = 84) |  | (N = 90) |  |
|  | Estimate (95% CI) | P-value | Estimate (95% CI) | P-value | Estimate (95% CI) | P-value |
| **Acylcarnitines** |  |  |  |  |  |  |
| C0 | 1.09 (0.95; 1.25) | 2.03E-01 | 1.03 (0.89; 1.19) | 6.72E-01 | 1.13 (0.92; 1.37) | 2.42E-01 |
| C2 | 1.04 (0.90; 1.20) | 6.15E-01 | 1.10 (0.92; 1.33) | 2.92E-01 | 1.00 (0.84; 1.17) | 9.57E-01 |
| C3 | 1.03 (0.89; 1.21) | 6.70E-01 | 1.04 (0.87; 1.24) | 6.96E-01 | 1.03 (0.80; 1.33) | 8.26E-01 |
| C3-DC (C4-OH) | 0.98 (0.87; 1.10) | 6.85E-01 | 1.00 (0.84; 1.19) | 9.70E-01 | 0.94 (0.82; 1.09) | 4.12E-01 |
| C3-OH | 0.98 (0.87; 1.12) | 7.92E-01 | 1.03 (0.89; 1.19) | 7.12E-01 | 0.95 (0.81; 1.12) | 5.74E-01 |
| C3:1 | 0.98 (0.87; 1.10) | 7.43E-01 | 0.99 (0.80; 1.22) | 8.93E-01 | 0.98 (0.85; 1.13) | 7.71E-01 |
| C4 | 1.09 (0.97; 1.23) | 1.45E-01 | 1.14 (1.05; 1.24) | 1.45E-03 | 1.00 (0.75; 1.34) | 9.96E-01 |
| C4:1 | 1.05 (0.92; 1.20) | 4.99E-01 | 1.02 (0.85; 1.22) | 8.26E-01 | 1.09 (0.94; 1.26) | 2.45E-01 |
| C5 | 1.04 (0.91; 1.18) | 5.99E-01 | 1.02 (0.86; 1.21) | 7.88E-01 | 1.04 (0.87; 1.24) | 6.50E-01 |
| C5-DC (C6-OH) | 1.07 (0.95; 1.22) | 2.55E-01 | 1.03 (0.87; 1.22) | 7.28E-01 | 1.07 (0.91; 1.24) | 4.11E-01 |
| C5-M-DC | 0.87 (0.77; 0.99) | 3.26E-02 | 1.04 (0.90; 1.21) | 5.94E-01 | 0.78 (0.68; 0.90) | 1.08E-03 |
| C5-OH (C3-DC-M) | 1.14 (1.01; 1.30) | 4.04E-02 | 1.15 (0.98; 1.36) | 9.38E-02 | 1.11 (0.94; 1.31) | 2.10E-01 |
| C5:1 | 0.94 (0.83; 1.07) | 3.82E-01 | 1.03 (0.86; 1.25) | 7.31E-01 | 0.90 (0.77; 1.05) | 1.80E-01 |
| C5:1-DC | 1.02 (0.89; 1.16) | 8.03E-01 | 0.88 (0.79; 0.98) | 2.21E-02 | 1.14 (0.94; 1.39) | 1.69E-01 |
| C6 (C4:1-DC) | 1.02 (0.89; 1.17) | 7.28E-01 | 0.94 (0.85; 1.05) | 2.71E-01 | 1.13 (0.89; 1.44) | 3.22E-01 |
| C6:1 | 1.12 (0.99; 1.26) | 7.02E-02 | 1.02 (0.88; 1.19) | 7.86E-01 | 1.16 (0.99; 1.36) | 6.15E-02 |
| C7-DC | 1.01 (0.88; 1.16) | 8.92E-01 | 1.11 (0.92; 1.35) | 2.77E-01 | 0.95 (0.80; 1.13) | 5.88E-01 |
| C8 | 0.98 (0.89; 1.08) | 6.73E-01 | 0.91 (0.85; 0.98) | 1.00E-02 | 1.17 (0.85; 1.63) | 3.32E-01 |
| C8:1 | 1.15 (1.03; 1.28) | 1.40E-02 | 1.13 (0.95; 1.34) | 1.60E-01 | 1.16 (1.04; 1.30) | 7.93E-03 |
| C9 | 1.09 (0.98; 1.22) | 1.07E-01 | 1.07 (0.91; 1.26) | 4.06E-01 | 1.05 (0.91; 1.20) | 5.29E-01 |
| C10 | 0.97 (0.87; 1.08) | 5.46E-01 | 0.91 (0.85; 0.99) | 1.98E-02 | 1.10 (0.80; 1.53) | 5.43E-01 |
| C10:1 | 1.02 (0.89; 1.16) | 7.63E-01 | 0.93 (0.82; 1.06) | 2.84E-01 | 1.13 (0.92; 1.39) | 2.40E-01 |
| C10:2 | 1.08 (0.94; 1.25) | 2.64E-01 | 0.97 (0.83; 1.14) | 7.31E-01 | 1.10 (0.93; 1.30) | 2.71E-01 |
| C12 | 0.98 (0.84; 1.15) | 8.00E-01 | 0.93 (0.81; 1.07) | 2.86E-01 | 1.15 (0.86; 1.55) | 3.41E-01 |
| C12-DC | 0.94 (0.83; 1.07) | 3.53E-01 | 1.04 (0.82; 1.32) | 7.40E-01 | 0.91 (0.79; 1.06) | 2.43E-01 |
| C12:1 | 0.93 (0.78; 1.11) | 4.12E-01 | 0.88 (0.74; 1.04) | 1.23E-01 | 1.02 (0.78; 1.33) | 9.06E-01 |
| C14 | 1.02 (0.88; 1.19) | 7.59E-01 | 0.99 (0.84; 1.15) | 8.59E-01 | 1.23 (0.96; 1.57) | 9.42E-02 |
| C14:1 | 0.90 (0.77; 1.04) | 1.49E-01 | 0.91 (0.72; 1.15) | 4.28E-01 | 0.99 (0.78; 1.24) | 9.01E-01 |
| C14:1-OH | 0.96 (0.85; 1.09) | 5.23E-01 | 0.99 (0.85; 1.16) | 9.08E-01 | 0.95 (0.79; 1.14) | 5.59E-01 |
| C14:2 | 0.89 (0.77; 1.02) | 9.42E-02 | 0.92 (0.75; 1.13) | 4.39E-01 | 0.93 (0.75; 1.16) | 5.17E-01 |
| C14:2-OH | 1.01 (0.91; 1.13) | 8.41E-01 | 1.00 (0.86; 1.16) | 9.98E-01 | 1.02 (0.90; 1.16) | 7.12E-01 |
| C16 | 1.10 (0.96; 1.27) | 1.73E-01 | 1.03 (0.89; 1.19) | 6.88E-01 | 1.19 (1.01; 1.40) | 3.81E-02 |
| C16-OH | 0.96 (0.83; 1.11) | 5.72E-01 | 1.11 (0.88; 1.40) | 3.93E-01 | 0.87 (0.75; 1.02) | 9.60E-02 |
| C16:1 | 0.90 (0.78; 1.04) | 1.69E-01 | 0.88 (0.75; 1.03) | 1.21E-01 | 0.98 (0.77; 1.23) | 8.34E-01 |
| C16:1-OH | 1.07 (0.95; 1.20) | 2.68E-01 | 1.07 (0.89; 1.29) | 4.87E-01 | 1.08 (0.95; 1.23) | 2.14E-01 |
| C16:2 | 0.94 (0.80; 1.09) | 4.11E-01 | 0.91 (0.74; 1.12) | 3.81E-01 | 1.01 (0.85; 1.22) | 8.72E-01 |
| C16:2-OH | 1.06 (0.93; 1.21) | 3.59E-01 | 0.87 (0.74; 1.03) | 1.03E-01 | 1.11 (0.95; 1.28) | 1.84E-01 |
| C18 | 1.06 (0.93; 1.20) | 4.09E-01 | 1.03 (0.91; 1.17) | 6.17E-01 | 1.08 (0.90; 1.30) | 3.83E-01 |
| C18:1 | 0.96 (0.83; 1.11) | 5.95E-01 | 0.90 (0.76; 1.07) | 2.43E-01 | 1.04 (0.86; 1.26) | 6.67E-01 |
| C18:1-OH | 1.02 (0.94; 1.11) | 5.86E-01 | 0.80 (0.63; 1.03) | 7.96E-02 | 1.05 (0.96; 1.15) | 2.61E-01 |
| C18:2 | 1.00 (0.87; 1.15) | 9.96E-01 | 0.99 (0.82; 1.19) | 8.97E-01 | 1.05 (0.89; 1.24) | 5.81E-01 |
| **Lysophosphatidylcholines** |  |  |  |  |  |  |
| Lyso PC a C6:0 | 1.05 (0.92; 1.20) | 4.60E-01 | 0.91 (0.75; 1.10) | 3.10E-01 | 1.14 (0.97; 1.34) | 1.15E-01 |
| Lyso PC a C14:0 | 1.19 (1.08; 1.32)* | 7.79E-04 | 1.23 (1.09; 1.39) | 8.94E-04 | 1.17 (1.01; 1.36) | 3.78E-02 |
| Lyso PC a C16:0 | 0.95 (0.83; 1.09) | 4.45E-01 | 1.04 (0.87; 1.23) | 6.79E-01 | 0.89 (0.75; 1.06) | 1.85E-01 |
| Lyso PC a C16:1 | 1.08 (0.96; 1.21) | 1.91E-01 | 1.14 (0.97; 1.34) | 1.21E-01 | 1.05 (0.91; 1.22) | 4.61E-01 |
| Lyso PC a C17:0 | 0.90 (0.78; 1.03) | 1.37E-01 | 0.97 (0.79; 1.18) | 7.26E-01 | 0.83 (0.69; 0.99) | 4.41E-02 |
| Lyso PC a C18:0 | 1.04 (0.91; 1.19) | 5.38E-01 | 1.02 (0.81; 1.29) | 8.40E-01 | 1.03 (0.89; 1.19) | 6.75E-01 |
| Lyso PC a C18:1 | 0.92 (0.80; 1.06) | 2.58E-01 | 0.87 (0.69; 1.10) | 2.39E-01 | 0.96 (0.80; 1.14) | 6.36E-01 |
| Lyso PC a C18:2 | 0.96 (0.83; 1.10) | 5.17E-01 | 0.98 (0.81; 1.19) | 8.61E-01 | 0.97 (0.78; 1.21) | 7.89E-01 |
| Lyso PC a C20:3 | 1.03 (0.90; 1.17) | 6.82E-01 | 0.97 (0.84; 1.13) | 7.33E-01 | 1.11 (0.92; 1.34) | 2.88E-01 |
| Lyso PC a C20:4 | 0.93 (0.81; 1.07) | 3.21E-01 | 0.88 (0.76; 1.03) | 1.14E-01 | 0.99 (0.80; 1.23) | 9.50E-01 |
| Lyso PC a C24:0 | 0.96 (0.86; 1.08) | 5.22E-01 | 0.95 (0.79; 1.14) | 5.90E-01 | 0.98 (0.87; 1.12) | 7.95E-01 |
| Lyso PC a C26:0 | 0.96 (0.86; 1.08) | 4.83E-01 | 0.99 (0.83; 1.18) | 9.29E-01 | 0.97 (0.85; 1.11) | 6.16E-01 |
| Lyso PC a C26:1 | 0.95 (0.85; 1.06) | 3.45E-01 | 0.99 (0.81; 1.21) | 9.34E-01 | 0.95 (0.84; 1.07) | 3.75E-01 |
| Lyso PC a C28:0 | 1.00 (0.89; 1.12) | 9.99E-01 | 1.08 (0.92; 1.27) | 3.54E-01 | 0.99 (0.87; 1.14) | 9.05E-01 |
| Lyso PC a C28:1 | 0.97 (0.86; 1.09) | 6.34E-01 | 1.07 (0.86; 1.33) | 5.53E-01 | 0.94 (0.82; 1.08) | 4.01E-01 |
| **Diacyl phosphatidylcholines** |  |  |  |  |  |  |
| PC aa C24:0 | 0.99 (0.88; 1.11) | 8.43E-01 | 1.03 (0.87; 1.23) | 7.05E-01 | 0.99 (0.87; 1.13) | 9.10E-01 |
| PC aa C26:0 | 0.97 (0.86; 1.10) | 6.50E-01 | 1.00 (0.84; 1.18) | 9.67E-01 | 0.97 (0.84; 1.12) | 6.85E-01 |
| PC aa C28:1 | 1.15 (0.98; 1.36) | 9.24E-02 | 1.11 (0.87; 1.41) | 3.89E-01 | 1.06 (0.85; 1.34) | 5.89E-01 |
| PC aa C30:0 | 1.16 (1.01; 1.32) | 2.94E-02 | 1.26 (1.07; 1.50) | 7.82E-03 | 1.09 (0.91; 1.31) | 3.53E-01 |
| PC aa C30:2 | 1.00 (0.88; 1.12) | 9.37E-01 | 1.02 (0.88; 1.18) | 7.73E-01 | 1.00 (0.84; 1.19) | 9.84E-01 |
| PC aa C32:0 | 1.00 (0.88; 1.14) | 9.93E-01 | 1.00 (0.84; 1.20) | 9.57E-01 | 0.97 (0.81; 1.16) | 7.50E-01 |
| PC aa C32:1 | 1.31 (1.14; 1.51)* | 2.77E-04 | 1.37 (1.11; 1.70) | 3.98E-03 | 1.26 (1.04; 1.52) | 1.88E-02 |
| PC aa C32:2 | 1.19 (1.03; 1.36) | 1.76E-02 | 1.26 (1.06; 1.52) | 1.18E-02 | 1.16 (0.98; 1.36) | 7.87E-02 |
| PC aa C32:3 | 1.10 (0.90; 1.33) | 3.61E-01 | 1.18 (0.98; 1.43) | 8.75E-02 | 1.09 (0.84; 1.43) | 5.07E-01 |
| PC aa C34:1 | 1.07 (0.89; 1.27) | 4.70E-01 | 1.16 (0.85; 1.57) | 3.40E-01 | 1.02 (0.81; 1.28) | 8.82E-01 |
| PC aa C34:2 | 1.03 (0.87; 1.22) | 7.14E-01 | 1.07 (0.79; 1.45) | 6.41E-01 | 0.98 (0.81; 1.18) | 8.15E-01 |
| PC aa C34:3 | 1.13 (0.94; 1.37) | 1.92E-01 | 1.20 (0.96; 1.50) | 1.07E-01 | 1.21 (0.94; 1.56) | 1.30E-01 |
| PC aa C34:4 | 1.22 (1.04; 1.44) | 1.60E-02 | 1.22 (1.00; 1.50) | 5.07E-02 | 1.22 (1.01; 1.47) | 3.84E-02 |
| PC aa C36:0 | 0.98 (0.88; 1.09) | 6.83E-01 | 0.93 (0.84; 1.03) | 1.58E-01 | 1.03 (0.87; 1.23) | 7.26E-01 |
| PC aa C36:1 | 1.30 (1.13; 1.50)* | 3.77E-04 | 1.19 (0.90; 1.58) | 2.15E-01 | 1.31 (1.11; 1.55) | 1.59E-03 |
| PC aa C36:2 | 1.16 (0.99; 1.35) | 5.99E-02 | 1.07 (0.83; 1.38) | 6.07E-01 | 1.17 (0.98; 1.39) | 8.45E-02 |
| PC aa C36:3 | 1.01 (0.84; 1.20) | 9.51E-01 | 1.01 (0.81; 1.25) | 9.32E-01 | 1.01 (0.80; 1.28) | 9.12E-01 |
| PC aa C36:4 | 0.96 (0.82; 1.11) | 5.69E-01 | 0.96 (0.78; 1.18) | 7.04E-01 | 0.96 (0.80; 1.17) | 7.12E-01 |
| PC aa C36:5 | 1.05 (0.92; 1.19) | 4.86E-01 | 0.96 (0.84; 1.11) | 5.83E-01 | 1.10 (0.92; 1.32) | 2.96E-01 |
| PC aa C36:6 | 1.13 (0.99; 1.29) | 6.71E-02 | 1.05 (0.89; 1.24) | 5.35E-01 | 1.14 (0.94; 1.36) | 1.73E-01 |
| PC aa C38:0 | 0.95 (0.85; 1.08) | 4.39E-01 | 0.89 (0.79; 1.01) | 8.03E-02 | 0.97 (0.82; 1.15) | 7.52E-01 |
| PC aa C38:1 | 0.92 (0.82; 1.04) | 1.77E-01 | 0.97 (0.86; 1.10) | 6.66E-01 | 0.91 (0.77; 1.06) | 2.13E-01 |
| PC aa C38:3 | 1.31 (1.11; 1.55) | 1.68E-03 | 1.21 (0.96; 1.53) | 1.07E-01 | 1.31 (1.06; 1.62) | 1.28E-02 |
| PC aa C38:4 | 1.08 (0.93; 1.26) | 3.08E-01 | 0.97 (0.80; 1.16) | 7.17E-01 | 1.13 (0.93; 1.37) | 2.28E-01 |
| PC aa C38:5 | 1.06 (0.92; 1.22) | 4.16E-01 | 0.95 (0.80; 1.12) | 5.31E-01 | 1.11 (0.90; 1.37) | 3.03E-01 |
| PC aa C38:6 | 1.00 (0.89; 1.13) | 9.86E-01 | 0.97 (0.85; 1.11) | 6.90E-01 | 0.97 (0.82; 1.15) | 7.55E-01 |
| PC aa C40:1 | 0.94 (0.84; 1.06) | 3.42E-01 | 0.90 (0.78; 1.03) | 1.15E-01 | 0.99 (0.85; 1.16) | 9.08E-01 |
| PC aa C40:2 | 0.97 (0.86; 1.09) | 5.98E-01 | 0.89 (0.77; 1.04) | 1.44E-01 | 1.03 (0.88; 1.20) | 7.35E-01 |
| PC aa C40:3 | 0.96 (0.85; 1.09) | 5.16E-01 | 0.93 (0.81; 1.06) | 2.88E-01 | 0.92 (0.77; 1.10) | 3.53E-01 |
| PC aa C40:4 | 1.13 (0.98; 1.30) | 8.50E-02 | 1.06 (0.88; 1.28) | 5.48E-01 | 1.14 (0.94; 1.37) | 1.76E-01 |
| PC aa C40:5 | 1.27 (1.11; 1.44)* | 4.64E-04 | 1.14 (0.95; 1.35) | 1.57E-01 | 1.28 (1.07; 1.53) | 7.50E-03 |
| PC aa C40:6 | 1.13 (1.02; 1.26) | 2.58E-02 | 1.03 (0.90; 1.18) | 6.71E-01 | 1.16 (0.98; 1.37) | 9.22E-02 |
| PC aa C42:0 | 0.93 (0.82; 1.05) | 2.41E-01 | 0.87 (0.73; 1.03) | 9.97E-02 | 0.96 (0.82; 1.12) | 5.99E-01 |
| PC aa C42:1 | 0.94 (0.84; 1.06) | 3.28E-01 | 0.84 (0.72; 0.99) | 3.87E-02 | 0.98 (0.85; 1.13) | 7.99E-01 |
| PC aa C42:2 | 0.96 (0.85; 1.08) | 4.74E-01 | 0.88 (0.77; 1.02) | 8.29E-02 | 1.02 (0.88; 1.18) | 8.26E-01 |
| PC aa C42:4 | 0.90 (0.80; 1.03) | 1.16E-01 | 0.83 (0.66; 1.05) | 1.12E-01 | 0.92 (0.79; 1.08) | 2.91E-01 |
| PC aa C42:5 | 1.01 (0.88; 1.15) | 9.20E-01 | 0.97 (0.85; 1.11) | 6.76E-01 | 1.00 (0.81; 1.25) | 9.67E-01 |
| PC aa C42:6 | 0.98 (0.86; 1.11) | 7.13E-01 | 0.93 (0.80; 1.08) | 3.41E-01 | 0.92 (0.76; 1.10) | 3.47E-01 |
| **Acyl-alkyl phosphatidylcholines** |  |  |  |  |  |  |
| PC ae C30:0 | 1.01 (0.89; 1.15) | 8.30E-01 | 1.07 (0.89; 1.29) | 4.56E-01 | 0.96 (0.82; 1.12) | 5.86E-01 |
| PC ae C30:1 | 0.99 (0.88; 1.11) | 8.26E-01 | 0.96 (0.79; 1.16) | 6.64E-01 | 0.98 (0.86; 1.13) | 8.09E-01 |
| PC ae C30:2 | 1.00 (0.88; 1.14) | 9.79E-01 | 0.99 (0.80; 1.23) | 9.50E-01 | 1.01 (0.85; 1.20) | 9.22E-01 |
| PC ae C32:1 | 1.00 (0.86; 1.16) | 9.89E-01 | 0.96 (0.78; 1.19) | 7.27E-01 | 1.00 (0.81; 1.24) | 9.71E-01 |
| PC ae C32:2 | 0.96 (0.82; 1.12) | 6.11E-01 | 0.87 (0.71; 1.06) | 1.58E-01 | 0.98 (0.79; 1.23) | 8.90E-01 |
| PC ae C34:0 | 1.01 (0.87; 1.17) | 8.98E-01 | 1.09 (0.92; 1.30) | 3.28E-01 | 0.90 (0.75; 1.09) | 2.73E-01 |
| PC ae C34:1 | 0.91 (0.77; 1.06) | 2.28E-01 | 1.00 (0.80; 1.25) | 9.79E-01 | 0.87 (0.71; 1.08) | 2.05E-01 |
| PC ae C34:2 | 0.96 (0.82; 1.12) | 6.22E-01 | 0.99 (0.80; 1.21) | 8.91E-01 | 0.94 (0.78; 1.15) | 5.49E-01 |
| PC ae C34:3 | 1.08 (0.91; 1.27) | 3.84E-01 | 0.99 (0.77; 1.28) | 9.42E-01 | 1.12 (0.93; 1.35) | 2.34E-01 |
| PC ae C36:0 | 0.96 (0.83; 1.11) | 5.82E-01 | 0.97 (0.83; 1.13) | 6.83E-01 | 0.95 (0.75; 1.19) | 6.34E-01 |
| PC ae C36:1 | 0.96 (0.81; 1.13) | 5.83E-01 | 1.09 (0.87; 1.37) | 4.27E-01 | 0.85 (0.70; 1.02) | 7.95E-02 |
| PC ae C36:2 | 0.84 (0.73; 0.96) | 1.15E-02 | 0.94 (0.77; 1.15) | 5.52E-01 | 0.76 (0.65; 0.90) | 1.96E-03 |
| PC ae C36:3 | 0.98 (0.83; 1.15) | 7.76E-01 | 0.95 (0.79; 1.16) | 6.37E-01 | 0.98 (0.79; 1.21) | 8.17E-01 |
| PC ae C36:4 | 1.01 (0.85; 1.20) | 8.74E-01 | 0.94 (0.78; 1.12) | 4.59E-01 | 1.03 (0.81; 1.32) | 7.80E-01 |
| PC ae C36:5 | 1.02 (0.86; 1.20) | 8.17E-01 | 0.87 (0.76; 1.00) | 4.59E-02 | 1.13 (0.92; 1.39) | 2.39E-01 |
| PC ae C38:0 | 1.03 (0.89; 1.19) | 6.90E-01 | 0.92 (0.78; 1.09) | 3.24E-01 | 1.02 (0.84; 1.24) | 8.44E-01 |
| PC ae C38:1 | 0.94 (0.83; 1.05) | 2.70E-01 | 0.96 (0.82; 1.13) | 6.42E-01 | 0.92 (0.78; 1.07) | 2.77E-01 |
| PC ae C38:2 | 0.91 (0.78; 1.06) | 2.35E-01 | 0.94 (0.76; 1.17) | 5.96E-01 | 0.87 (0.72; 1.05) | 1.47E-01 |
| PC ae C38:3 | 0.91 (0.77; 1.08) | 2.72E-01 | 0.94 (0.76; 1.17) | 6.00E-01 | 0.84 (0.68; 1.04) | 1.11E-01 |
| PC ae C38:4 | 0.93 (0.81; 1.06) | 2.65E-01 | 0.93 (0.77; 1.13) | 4.59E-01 | 0.90 (0.76; 1.06) | 1.95E-01 |
| PC ae C38:5 | 0.93 (0.80; 1.08) | 3.59E-01 | 0.87 (0.73; 1.02) | 8.43E-02 | 0.96 (0.78; 1.18) | 6.79E-01 |
| PC ae C38:6 | 0.99 (0.88; 1.12) | 9.21E-01 | 0.91 (0.81; 1.01) | 8.83E-02 | 1.04 (0.87; 1.26) | 6.44E-01 |
| PC ae C40:0 | 0.89 (0.79; 1.02) | 9.39E-02 | 0.79 (0.66; 0.94) | 9.50E-03 | 0.89 (0.75; 1.07) | 2.09E-01 |
| PC ae C40:1 | 0.96 (0.82; 1.12) | 6.11E-01 | 0.86 (0.71; 1.03) | 9.55E-02 | 0.92 (0.75; 1.14) | 4.55E-01 |
| PC ae C40:2 | 0.94 (0.81; 1.08) | 3.80E-01 | 1.01 (0.83; 1.22) | 9.24E-01 | 0.83 (0.69; 0.98) | 2.95E-02 |
| PC ae C40:3 | 0.91 (0.78; 1.07) | 2.62E-01 | 0.97 (0.76; 1.25) | 8.39E-01 | 0.85 (0.72; 1.01) | 6.17E-02 |
| PC ae C40:4 | 0.92 (0.80; 1.06) | 2.68E-01 | 0.93 (0.74; 1.16) | 5.06E-01 | 0.91 (0.77; 1.08) | 2.81E-01 |
| PC ae C40:5 | 0.91 (0.79; 1.04) | 1.67E-01 | 0.83 (0.70; 0.98) | 2.87E-02 | 0.91 (0.77; 1.09) | 3.12E-01 |
| PC ae C40:6 | 0.93 (0.82; 1.06) | 2.84E-01 | 0.91 (0.76; 1.08) | 2.56E-01 | 0.90 (0.75; 1.07) | 2.32E-01 |
| PC ae C42:0 | 0.86 (0.78; 0.95) | 2.71E-03 | 0.81 (0.70; 0.94) | 6.17E-03 | 0.88 (0.78; 0.99) | 3.05E-02 |
| PC ae C42:1 | 0.97 (0.85; 1.10) | 6.11E-01 | 0.91 (0.74; 1.10) | 3.22E-01 | 0.96 (0.83; 1.13) | 6.46E-01 |
| PC ae C42:2 | 0.96 (0.82; 1.13) | 6.36E-01 | 0.88 (0.72; 1.07) | 1.94E-01 | 0.93 (0.76; 1.15) | 5.10E-01 |
| PC ae C42:3 | 0.91 (0.80; 1.04) | 1.63E-01 | 0.88 (0.73; 1.07) | 2.06E-01 | 0.88 (0.75; 1.03) | 1.12E-01 |
| PC ae C42:4 | 0.93 (0.81; 1.07) | 3.19E-01 | 0.94 (0.76; 1.18) | 6.03E-01 | 0.92 (0.77; 1.10) | 3.79E-01 |
| PC ae C42:5 | 0.90 (0.78; 1.03) | 1.22E-01 | 0.82 (0.68; 0.99) | 4.43E-02 | 0.94 (0.80; 1.11) | 4.56E-01 |
| PC ae C44:3 | 1.05 (0.92; 1.18) | 4.75E-01 | 0.99 (0.80; 1.24) | 9.43E-01 | 1.05 (0.92; 1.19) | 4.92E-01 |
| PC ae C44:4 | 0.99 (0.87; 1.13) | 8.88E-01 | 0.97 (0.78; 1.20) | 7.78E-01 | 1.04 (0.90; 1.19) | 6.08E-01 |
| PC ae C44:5 | 0.94 (0.81; 1.09) | 4.32E-01 | 0.89 (0.70; 1.14) | 3.57E-01 | 0.99 (0.84; 1.15) | 8.65E-01 |
| PC ae C44:6 | 0.93 (0.82; 1.07) | 3.03E-01 | 0.90 (0.71; 1.15) | 3.94E-01 | 0.97 (0.84; 1.11) | 6.53E-01 |
| **Sphingomyelins** |  |  |  |  |  |  |
| SM (OH) C14:1 | 0.94 (0.79; 1.10) | 4.23E-01 | 0.96 (0.77; 1.21) | 7.39E-01 | 0.88 (0.70; 1.10) | 2.65E-01 |
| SM (OH) C16:1 | 0.93 (0.80; 1.09) | 3.62E-01 | 0.96 (0.78; 1.19) | 7.14E-01 | 0.87 (0.70; 1.08) | 2.00E-01 |
| SM (OH) C22:1 | 1.16 (0.98; 1.38) | 8.16E-02 | 1.17 (0.92; 1.49) | 1.87E-01 | 1.13 (0.91; 1.41) | 2.50E-01 |
| SM (OH) C22:2 | 0.96 (0.82; 1.13) | 6.44E-01 | 1.16 (0.93; 1.45) | 1.77E-01 | 0.82 (0.67; 0.99) | 4.34E-02 |
| SM (OH) C24:1 | 1.11 (0.95; 1.30) | 1.95E-01 | 1.08 (0.87; 1.35) | 4.83E-01 | 1.04 (0.88; 1.24) | 6.38E-01 |
| SM C16:0 | 0.94 (0.81; 1.09) | 4.04E-01 | 0.91 (0.73; 1.13) | 3.81E-01 | 0.97 (0.79; 1.19) | 7.53E-01 |
| SM C16:1 | 0.95 (0.82; 1.10) | 4.86E-01 | 1.05 (0.83; 1.32) | 6.87E-01 | 0.92 (0.78; 1.09) | 3.29E-01 |
| SM C18:0 | 1.06 (0.94; 1.21) | 3.38E-01 | 1.09 (0.89; 1.32) | 3.96E-01 | 1.01 (0.87; 1.17) | 9.03E-01 |
| SM C18:1 | 0.96 (0.84; 1.09) | 5.29E-01 | 1.06 (0.89; 1.27) | 5.00E-01 | 0.89 (0.75; 1.06) | 1.73E-01 |
| SM C20:2 | 0.92 (0.80; 1.07) | 2.79E-01 | 1.25 (1.03; 1.52) | 2.69E-02 | 0.81 (0.67; 0.99) | 3.65E-02 |
| SM C22:3 | 0.91 (0.82; 1.02) | 9.93E-02 | 1.03 (0.85; 1.25) | 7.78E-01 | 0.90 (0.79; 1.02) | 1.07E-01 |
| SM C24:0 | 1.11 (0.96; 1.28) | 1.41E-01 | 1.04 (0.86; 1.27) | 6.80E-01 | 1.18 (0.97; 1.43) | 9.12E-02 |
| SM C24:1 | 0.95 (0.83; 1.08) | 4.28E-01 | 1.02 (0.84; 1.25) | 8.19E-01 | 0.92 (0.78; 1.07) | 2.76E-01 |
| SM C26:0 | 1.00 (0.88; 1.13) | 9.62E-01 | 1.17 (0.95; 1.44) | 1.29E-01 | 0.91 (0.79; 1.05) | 2.00E-01 |
| SM C26:1 | 0.98 (0.87; 1.11) | 7.64E-01 | 1.00 (0.87; 1.16) | 9.69E-01 | 0.92 (0.80; 1.06) | 2.38E-01 |
| **Amino acids** |  |  |  |  |  |  |
| Arginine | 1.02 (0.91; 1.14) | 7.18E-01 | 0.94 (0.83; 1.07) | 3.66E-01 | 1.05 (0.89; 1.23) | 5.84E-01 |
| Glutamine | 1.06 (0.94; 1.20) | 3.10E-01 | 1.16 (0.99; 1.36) | 7.16E-02 | 1.00 (0.87; 1.15) | 9.91E-01 |
| Glycine | 1.02 (0.91; 1.14) | 7.12E-01 | 1.37 (0.98; 1.93) | 6.67E-02 | 0.98 (0.87; 1.10) | 7.01E-01 |
| Histidine | 1.03 (0.90; 1.17) | 6.83E-01 | 1.28 (1.06; 1.53) | 9.51E-03 | 0.91 (0.80; 1.04) | 1.54E-01 |
| Methionine | 1.11 (0.96; 1.28) | 1.68E-01 | 1.23 (1.06; 1.44) | 7.95E-03 | 1.01 (0.83; 1.23) | 9.41E-01 |
| Ornithine | 1.05 (0.92; 1.19) | 4.76E-01 | 1.08 (0.91; 1.28) | 4.02E-01 | 0.94 (0.79; 1.12) | 4.68E-01 |
| Phenylalanine | 1.11 (0.98; 1.26) | 9.68E-02 | 1.12 (0.97; 1.29) | 1.31E-01 | 1.05 (0.88; 1.25) | 5.74E-01 |
| Proline | 1.00 (0.92; 1.09) | 9.66E-01 | 1.05 (0.94; 1.16) | 3.88E-01 | 1.01 (0.83; 1.25) | 8.90E-01 |
| Serine | 0.96 (0.85; 1.08) | 4.73E-01 | 1.23 (0.93; 1.63) | 1.51E-01 | 0.91 (0.80; 1.03) | 1.34E-01 |
| Threonine | 0.89 (0.79; 1.00) | 5.31E-02 | 0.95 (0.81; 1.12) | 5.49E-01 | 0.87 (0.76; 1.00) | 4.52E-02 |
| Tryptophan | 1.16 (1.04; 1.29) | 7.46E-03 | 1.16 (1.01; 1.33) | 3.62E-02 | 1.09 (0.94; 1.27) | 2.51E-01 |
| Tyrosine | 1.28 (1.08; 1.52) | 5.61E-03 | 1.43 (1.26; 1.62) | 4.34E-07 | 1.16 (0.92; 1.45) | 2.04E-01 |
| Valine | 1.02 (0.90; 1.17) | 7.19E-01 | 1.07 (0.89; 1.29) | 4.83E-01 | 0.96 (0.82; 1.13) | 6.31E-01 |
| (Iso)Leucine | 1.07 (0.92; 1.24) | 3.57E-01 | 1.05 (0.92; 1.21) | 4.55E-01 | 1.02 (0.77; 1.34) | 8.90E-01 |
| **Hexoses** |  |  |  |  |  |  |
| Hexoses | 1.11 (0.97; 1.28) | 1.31E-01 | 1.08 (0.93; 1.26) | 2.86E-01 | 1.13 (0.94; 1.36) | 1.85E-01 |
| **Aggregate measures** |  |  |  |  |  |  |
| (C2 + C3) / C0 | 0.96 (0.81; 1.13) | 6.08E-01 | 1.09 (0.87; 1.38) | 4.45E-01 | 0.90 (0.75; 1.07) | 2.36E-01 |
| Aromatic amino acids (AAA) | 1.24 (1.08; 1.41) | 2.15E-03 | 1.29 (1.13; 1.48) | 3.91E-04 | 1.14 (0.94; 1.37) | 1.82E-01 |
| C2 / C0 | 0.96 (0.81; 1.14) | 6.28E-01 | 1.09 (0.86; 1.38) | 4.64E-01 | 0.90 (0.75; 1.08) | 2.51E-01 |
| (C16 + C18) / C0 | 0.96 (0.84; 1.11) | 6.06E-01 | 1.00 (0.85; 1.17) | 9.68E-01 | 0.98 (0.81; 1.19) | 8.58E-01 |
| MUFA(PC) | 1.15 (0.96; 1.38) | 1.30E-01 | 1.21 (0.89; 1.65) | 2.14E-01 | 1.10 (0.87; 1.40) | 4.06E-01 |
| MUFA(PC) / SFA(PC) | 1.08 (0.92; 1.25) | 3.41E-01 | 1.17 (0.99; 1.38) | 7.37E-02 | 1.05 (0.86; 1.29) | 6.08E-01 |
| Ornithine / Arginine | 1.03 (0.93; 1.14) | 5.97E-01 | 1.08 (0.96; 1.21) | 1.99E-01 | 0.90 (0.76; 1.07) | 2.49E-01 |
| PUFA(PC) | 1.09 (0.91; 1.31) | 3.24E-01 | 1.00 (0.78; 1.28) | 9.85E-01 | 1.09 (0.87; 1.37) | 4.34E-01 |
| PUFA(PC) / MUFA(PC) | 0.98 (0.85; 1.12) | 7.34E-01 | 0.88 (0.71; 1.08) | 2.24E-01 | 1.00 (0.84; 1.19) | 9.96E-01 |
| PUFA(PC) / SFA(PC) | 1.04 (0.93; 1.16) | 4.65E-01 | 1.03 (0.91; 1.16) | 6.73E-01 | 1.08 (0.89; 1.32) | 4.24E-01 |
| SFA(PC) | 1.02 (0.89; 1.17) | 8.00E-01 | 0.99 (0.84; 1.17) | 9.32E-01 | 0.99 (0.81; 1.21) | 9.51E-01 |
| Total PC + Total SM | 1.09 (0.91; 1.31) | 3.29E-01 | 1.04 (0.79; 1.36) | 7.86E-01 | 1.08 (0.86; 1.35) | 4.95E-01 |
| Total AC / C0 | 0.93 (0.79; 1.11) | 4.36E-01 | 1.06 (0.82; 1.37) | 6.67E-01 | 0.89 (0.74; 1.07) | 2.04E-01 |
| Total AC-DC / Total AC | 0.94 (0.81; 1.09) | 4.29E-01 | 0.99 (0.82; 1.18) | 8.89E-01 | 0.92 (0.77; 1.10) | 3.55E-01 |
| Total AC-OH / Total AC | 0.97 (0.85; 1.11) | 6.52E-01 | 0.94 (0.79; 1.13) | 5.11E-01 | 0.96 (0.83; 1.12) | 6.36E-01 |
| Total lysoPC | 0.96 (0.84; 1.11) | 5.87E-01 | 1.00 (0.83; 1.22) | 9.64E-01 | 0.95 (0.79; 1.14) | 5.66E-01 |
| Total lysoPC / Total PC | 0.92 (0.79; 1.08) | 3.21E-01 | 0.97 (0.74; 1.27) | 8.16E-01 | 0.94 (0.78; 1.14) | 5.34E-01 |
| Total PC | 1.12 (0.93; 1.34) | 2.32E-01 | 1.04 (0.79; 1.36) | 7.69E-01 | 1.11 (0.88; 1.40) | 3.88E-01 |
| Total diacyl PC | 1.14 (0.95; 1.37) | 1.45E-01 | 1.07 (0.81; 1.40) | 6.24E-01 | 1.13 (0.90; 1.42) | 2.96E-01 |
| Total acyl-alkyl PC | 0.92 (0.78; 1.09) | 3.45E-01 | 0.88 (0.72; 1.07) | 1.91E-01 | 0.91 (0.72; 1.15) | 4.22E-01 |
| Total SM | 0.98 (0.83; 1.15) | 7.95E-01 | 1.02 (0.80; 1.29) | 8.92E-01 | 0.96 (0.79; 1.16) | 6.53E-01 |
| Total SM / (Total SM + Total PC) | 0.89 (0.77; 1.03) | 1.23E-01 | 0.95 (0.78; 1.17) | 6.56E-01 | 0.91 (0.75; 1.11) | 3.60E-01 |
| Total SM / Total PC | 0.89 (0.77; 1.03) | 1.26E-01 | 0.95 (0.78; 1.18) | 6.58E-01 | 0.92 (0.75; 1.12) | 3.82E-01 |
| Total SM-non OH | 0.97 (0.83; 1.13) | 7.04E-01 | 1.00 (0.79; 1.26) | 9.91E-01 | 0.96 (0.80; 1.16) | 6.81E-01 |
| Total SM-OH | 1.04 (0.87; 1.23) | 6.97E-01 | 1.13 (0.87; 1.45) | 3.63E-01 | 0.94 (0.75; 1.17) | 5.84E-01 |
| Total SM-OH / Total SM-non OH | 1.06 (0.92; 1.21) | 4.23E-01 | 1.10 (0.88; 1.39) | 4.01E-01 | 0.99 (0.85; 1.15) | 8.83E-01 |
| Tyrosine / Phenylalanine | 1.27 (1.10; 1.47) | 1.49E-03 | 1.43 (1.21; 1.69) | 5.70E-05 | 1.15 (0.96; 1.38) | 1.21E-01 |
